# Implications of Pyrolytic Gas Dynamic Evolution on Dissolved Black Carbon Formed During Production of Biochar from Nitrogen-Rich Feedstock

**DOI:** 10.1021/acs.est.4c08231

**Published:** 2025-01-13

**Authors:** Xiaoxiao Zhang, Zibo Xu, Yuqing Sun, Sanjay K. Mohanty, Hanwu Lei, Eakalak Khan, Daniel C. W. Tsang

**Affiliations:** † Department of Civil and Environmental Engineering, The Hong Kong Polytechnic University, Hung Hom, Kowloon, Hong Kong 999077, China; ‡ Department of Civil and Environmental Engineering, The Hong Kong University of Science and Technology, Clear Water Bay, Hong Kong 999077, China; § School of Agriculture, Sun Yat-sen University, Shenzhen 518107, Guangdong, China; ∥ Civil and Environmental Engineering Department, University of California Los Angeles, Los Angeles, California 90095, United States; ⊥ Department of Biological Systems Engineering, Washington State University, Richland, Washington 99354-1671, United States; # Civil and Environmental Engineering and Construction Department, University of Nevada, Las Vegas, Nevada 89154-4015, United States

**Keywords:** pyrogenic carbon, heterogeneous correlations, molecular diversity, engineered biochar, sustainable waste management, carbon sequestration

## Abstract

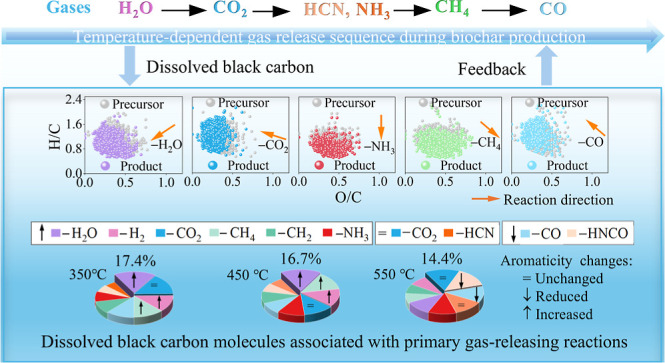

Gases and dissolved black carbon (DBC) formed during pyrolysis of nitrogen-rich feedstock would affect atmospheric and aquatic environments. Yet, the mechanisms driving biomass gas evolution and DBC formation are poorly understood. Using thermogravimetric-Fourier transform infrared spectrometry and two-dimensional correlation spectroscopy, we correlated the temperature-dependent primary noncondensable gas release sequence (H_2_O → CO_2_ → HCN, NH_3_ → CH_4_ → CO) with specific defunctionalization stages in the order: dehydration, decarboxylation, denitrogenation, demethylation, and decarbonylation. Our results revealed that proteins in feedstock mainly contributed to gas releases, and low-volatile pyrolytic products contributed to DBC. Combining mass difference analysis with Fourier transform ion cyclotron resonance mass spectrometry, we showed that 44–60% of DBC molecular compositions were correlated with primary gas-releasing reactions. Dehydration (−H_2_O), with lower reaction energy barrier, contributed to DBC formation most at 350 and 450 °C, whereas decarboxylation (−CO_2_) and deamidization (−HCNO) prevailed in contributing to DBC formation at 550 °C. The aromaticity changes of DBC products formed via gas emissions were deduced. Compared to their precursors, dehydration increased DBC aromaticity, while deamidization reduced the aromaticity of DBC products. These insights on pyrolytic byproducts help predict and tune DBC properties via changing gas formed during biochar production, minimizing their negative environmental impacts.

## Abstract 2

Temperature-dependent primary gas releases drive approximately 50% of dissolved black carbon formation during nitrogen-rich biomass pyrolysis, offering crucial information for predicting and mitigating their environmental impacts.

## 1 Introduction

Anaerobic digestion is a preferred technology to recycle organic wastes, such as food waste (∼1.3 billion tons per year globally), into renewable energy.^1^ However, this process leaves behind considerable nitrogen-rich digestate, which is difficult to treat or reuse.^2,3^ As suggested by recent studies, pyrolysis can transform the nitrogen-rich feedstock such as food waste digestate (FWD) into different value-added products for environmental and engineering applications.^4,5^ These products include gas (noncondensable), tar (condensable volatiles), and char residue. Gas and tar can be used as fuels or chemicals,^6,7^ whereas char can be used for carbon sequestration and soil fertility enhancement.^8,9^ However, the interactions of pyrolysis gas and tar with carbonized biomass would unavoidably result in the formation of mobile organic compounds, such as dissolved black carbon (DBC) and dissolved black nitrogen. DBC is released into water environments during biochar applications, with many unintended consequences and potential environmental concerns.^10,11^ Thus, it is important to examine the mechanisms behind the formation of DBC as a consequence of three-phase interactions among gas, tar, and char during pyrolysis so that the production process can be adequately designed to deliver desirable characteristics of DBC, minimizing environmental impacts. The DBC properties depend on the specific feedstock and pyrolysis conditions.^12,13^ For instance, increasing temperature can transform nitrogen precursors in rice straw from labile N-chains to aromatic structures.^14^ However, critical factors driving these processes remain unclear.

The interactions among gas, tar, and char during pyrolysis are complex and usually temperature-dependent.^14,15^ Higher pyrolysis temperatures decrease tar and char yields while increase gas production,^15^ which can be attributed to the transformation of tar and char into gas through cracking and sequential/parallel defunctionalization processes.^16^ The evolution of volatile products and residual char can be analyzed using online thermogravimetric analysis and Fourier transform infrared spectrometry-mass spectrometry (TG-FTIR-MS) coupled with two-dimensional correlation spectroscopy (2D-COS) analysis.^17^ Notably, the volatile products identified include both small noncondensable stable gases (e.g., H_2_O, CO_2_) and volatile organic compounds (VOCs).^17,18^ Information on stable gases (e.g., H_2_O, CO_2_) can provide insights into the defunctionalization processes (e.g., dehydration, decarboxylation). VOCs and residual functional groups in char produced during the pyrolysis of coal and biomass fuels have been recently identified using online TG-FTIR and 2D-COS analysis.^19^ The result revealed that the temperature-dependent variations of functional groups in VOCs and char and the formation of noncondensable small gases occurred synchronously. A holistic analysis of their evolutions could provide new insights into the DBC formation mechanism and a rational design of biomass pyrolysis processes.

It has been suggested that DBC is released from char via a surface wash-off process.^20^ Compounds rich in oxygen- and nitrogen-containing groups (e.g., hydroxyl (−OH), carboxyl (−COOH), carbonyl (−C=O), amine (−NH_2_), amide (−CONH_2_), nitro (−NO_2_)) exhibit a low volatility and high solubility by facilitating hydrogen bonding and electrostatic interactions with water molecules, rendering them more likely to form DBC molecules during leaching.^20,21^ The aromatic structures and reactive functional groups of DBC molecules serve many functions in water environments.^22,23^ For example, aromatic compounds with more hydroxyl and sulfhydryl groups on their side chains show higher electron donating capacity because these functional groups can act as electron donors. Electron donating capacity manifested a positive correlation with chlorine demand and disinfection byproduct formation.^24^ In addition, DBC, with high contents of polycyclic aromatic hydrocarbons, would bind strongly with organic/inorganic contaminants, influencing their environmental risks.^23^ As DBC accumulates in natural waters due to the increasing popularity of biochar application,^10,25^ understanding its molecular diversity and formation mechanisms is crucial for predicting their interactions with coexisting pollutants^10,26^ and its impact on drinking water systems.^27,28^

Analogous to functional groups in biochar, the evolution of functional groups in DBC molecules involves complex temperature-dependent defunctionalization reactions, accompanied by gas releases. Understanding defunctionalization processes is essential to predict and control the DBC formation.^29^ The DBC molecules, rich in functional groups and having *m*/*z* close to those of their precursors,^26^ may form through primary or secondary defunctionalization processes, releasing one or two gaseous molecules. Mass difference analysis is conducive to illuminating the reaction mechanisms of complex matrix by considering specific functional loss and/or reagent addition between precursor-product pairs identified by the Fourier transform ion cyclotron resonance-mass spectrometry (FTICR-MS).^30,31^ Currently, mass difference analysis has increasingly been applied for diverse purposes including formula assignment,^32^ untargeted metabolite profiling,^33^ and chemical reaction pathway identification.^34^ However, it is rarely applied in determining the DBC formation mechanisms. Recent studies identified Maillard reaction products during organic burning by using the mass difference analysis, focusing on amino acid condensation and dehydration reactions.^31,35^ However, to the best of our knowledge, no research has elucidated these primary defunctionalization processes linking gas releases with DBC formation.

Our study aims to (1) identify the temperature-dependent dynamic evolution of gaseous compounds during FWD pyrolysis using a 2D-COS analysis of TG-FTIR-MS data, (2) determine the molecular features of compounds released from FWD pyrolysis and the DBC samples leached from FWD-derived biochar at specific temperatures, (3) reveal the connections between the pyrolytic gases and DBC molecules at the molecular level via a mass difference analysis, and (4) elaborate the impact of gas-releasing reactions on DBC composition and aromaticity. The linkages among gas evolutions, biochar properties, and DBC patterns could shed light on how DBC molecules are formed, so that their quality can be tuned for different environmental applications and undesirable impact minimization.

## 2 Materials and Methods

### 2.1 Source of N-Rich Biomass, Production of Biochar and Dissolved Black Carbon

Food waste digestate, collected from O•PARK1 in Hong Kong, was chosen for its high nitrogen content.^20,36^ The FWD was pretreated, and its components (hemicellulose, cellulose, lignin, and extractives), element composition, and solubility were analyzed to understand their pyrolytic behaviors (Note S1.1, Supporting Information). To examine how pyrolysis temperature affects the biochar and DBC properties, we produced biochar from FWD at 7 temperatures ranging from 250 to 850 °C in 100 °C increments. The pyrolysis was conducted in a tubular furnace, heating FWD at a rate of 10 °C·min^–1^ under a nitrogen flow of 300 mL·min^–1^ and held at the target temperature for 2 h. The resulting biochar was cooled to room temperature and labeled according to their pyrolysis temperatures (BC250 to BC850). DBC was collected by leaching biochar in a pH 5 solution for 2 days according to a previous study,^20^ followed by filtration through a 0.45 μm pore-size glass microfiber filter. The pH was adjusted initially with 60:40 (w/w) H_2_SO_4_/HNO_3_ and ranged from 7.1 to 8.1 for the 48 h DBC samples. The DBC solutions and the biochar after leaching (BCA) on membranes were collected and labeled according to their pyrolysis temperatures (DBC250 to DBC850 and BCA250 to BCA850).

### 2.2 Thermogravimetric, Gaussian Model, and Thermodynamic Analyses of FWD

Thermogravimetric analyses were conducted to examine the thermal degradation behavior of FWD. Approximately 8.6 ± 0.2 mg of pretreated FWD was heated from room temperature to 900 °C at different heating rates (10, 20, 30, 40, 50 °C·min^–1^) using a thermal analyzer (PerkinElmer, TGA8000) under a nitrogen atmosphere at 60 mL·min^–1^. The derivative thermogravimetric (DTG) curves were analyzed with a Gaussian model to quantify the contributions of different components to the overall weight loss (Note S1.2).^17^ The pyrolytic mechanism of FWD was elucidated by calculating thermodynamic parameters, including the apparent activation energy (*E*_α_), pre-exponential factor (*A*), changes in enthalpy (Δ*H*), Gibb’s free energy (Δ*G*), and entropy (Δ*S*), using the Flynn–Wall–Ozawa free model^37^
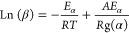
1where α, β, and *R* are the conversion rate, constant heating rate (K·s^–1^), ideal gas constant (*R* = 8.3145 J·mol^–1^·K^–1^), respectively. Detailed calculation processes are provided in Notes S1.3.

### 2.3 Characterization of Gaseous Compounds During FWD Pyrolysis

Gaseous compounds released during FWD pyrolysis were analyzed using TG–FTIR–MS. A heating rate of 10 °C·min^–1^ was adopted for the TG analysis (PerkinElmer TGA8000), similar to the heating rate of biochar production. Upon purging with nitrogen gas at 60 mL·min^–1^, the evolved gases were detected with an FTIR spectrometer (PerkinElmer Frontier) at 4000–600 cm^–1^ with 4 cm^–1^ resolution. The transfer-line was kept at 220 °C to minimize condensation. A semiquantitative method was used to determine the changes of cumulative content percentages of the gaseous mixtures at specific temperatures (or temperature ranges) by integrating the FTIR absorbance spectra (Note S1.4). An offline gas chromatography/mass spectrometer (GC–MS, PerkinElmer Clarus SQ8) was used to identify structural information on volatile organic compounds (VOCs) at key DTG/FTIR peak temperatures (350, 450, and 550 °C) by referencing spectra from the Wiley/NIST database. The saturation mass concentration (*C*_0_ value, μg·m^–3^) of VOCs was calculated to reflect volatility.^38^ The average carbon oxidation state () of VOCs was calculated to reflect their oxidation dynamics following .^39^ Additional details on the TG-FTIR-MS analysis, FTIR spectral integral methods, and VOC parameter calculations are provided in Note S1.4.

### 2.4 FTIR Testing of Biochar and Dissolved Black Carbon, and 2D-COS Analyses

The FTIR spectra of biochar, BCAs, and DBCs enriched through freeze-drying were recorded to determine their functional groups (Note S1.5).^21^ To study the temperature-dependent sequential responses of gaseous compounds, biochar functional groups, and DBC functional groups, generalized 2D-COS analyses were conducted for their FTIR data, respectively.^40^ Hetero 2D-FTIR-COS analyses were employed to understand the evolved relationships between gases and biochar functional groups. DBC was produced from biochar via surface wash-off processes,^20^ suggesting a connection in the temperature-dependent responses of functional groups between biochar and DBC. Therefore, hetero 2D-FTIR-COS analyses were performed between DBCs and BCAs to identify which functional groups were actively involved in the leaching processes.^26^ Synchronous and asynchronous maps were analyzed to interpret the synergistic relationships and sequences of spectral changes based on Noda’s rules.^41,42^ Detailed theory and interpretations of 2D-COS maps are provided in Note S1.6. Briefly, the overall change at variable *v*1 precedes variable *v*2 if the peak region/signals in synchronous/asynchronous maps align but reverse with different signs. If the synchronous signal is zero, the relationship becomes indeterminate.^26^

### 2.5 Extraction and Molecular Identification of Dissolved Black Carbon

**Figure 1:**
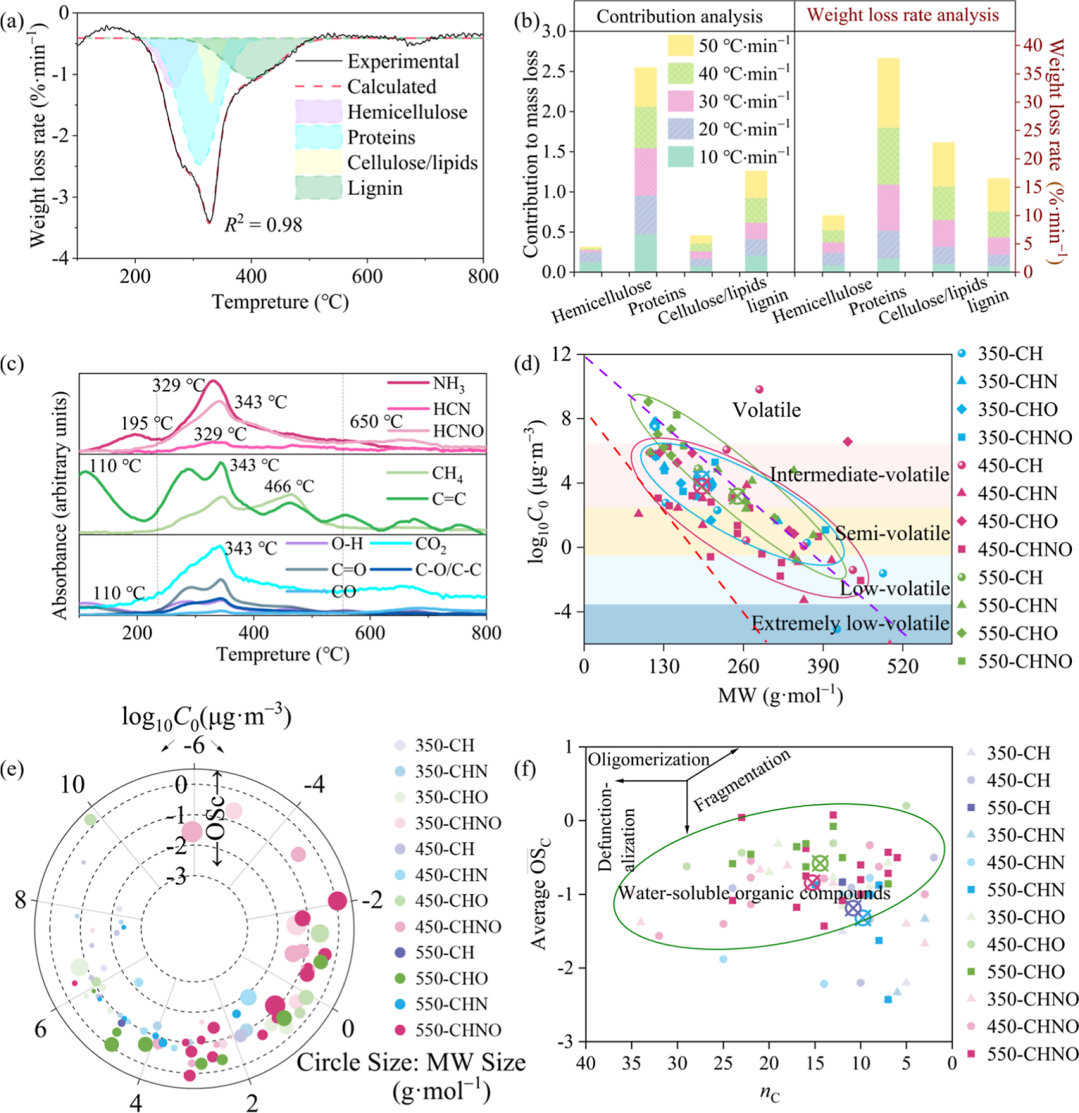
(a) Optimized decomposition process of components in FWD at 10 °C min^–1^ and (b) optimized decomposition parameters at five heating rates; (c) FTIR spectral profiles of gaseous products; (d) Log_10_*C*_0_ vs MW, (e) *C*_0_–MW, and (f)  vs carbon number (*n*_C_)^39^ for VOCs identified at 350, 450, and 550 °C. Upper (purple) and lower (red) boundaries in Log_10_*C*_0_ vs MW plot represent linear alkanes and sugar alcohols, respectively.^38^ Circular crossing markers represent the average values of parameters at (d) varying temperatures and (f) varying compound classes.

Significant decomposition and pyrolytic product releases of FWD occurred from 200 to 550 °C (Figure 1a–c). Thus, DBC350, DBC450, and DBC550 were solid-phase extracted and infused into a 15 T FTICR-MS (solariX, Bruker, USA) in both negative and positive electrospray ionization (−ESI and +ESI) modes for molecular identification. Dissolved FWD (DFWD) was collected following similar procedures with DBC and solid-phase extracted for FTICR-MS analysis as comparison. Molecular formulas were assigned to the peaks with signal-to-noise ratio >4 and mass error <1 ppm using the criteria of ^12^C_1–60_, ^13^C_0–1_, ^1^H_1–120_, ^16^O_1–50_, ^14^N_0–5_, ^32^S_0–2_, ^34^P_0–2_ (−ESI) and ^12^C_1–60_, ^13^C_0–1_, ^1^H_1–120_, ^16^O_1–50_, ^14^N_0–5_, ^32^S_0–2_, ^34^P_0–2_, ^23^Na_0–1_ (+ESI) in the Bruker Daltonics software (v4.2).^43,44^ A careful refinement was conducted (Note S1.7) to minimize incorrect formula assignments due to expanded elemental combinations. A procedural blank using PPL-extracted Milli-Q water was analyzed to assess contamination, and peaks from this blank were excluded from formula assignments. The identified formulas were categorized using the van Krevelen diagram based on their O/C and H/C ratios.^45^ Molecular characteristics of DFWD and DBC were described using the intensity-weighted parameters, i.e., intensity-weighted molecular weight (MW_w_), intensity-weighted double-bond equivalents (DBE_w_), intensity-weighted elemental ratios (H/C_w_, O/C_w_, and N/C_w_), intensity-weighted modified aromaticity index (AI_mod,w_).^44,46^ The *C*_0_ (μg·m^–3^) values of the DBC molecules were calculated to reveal their volatility.^21^ Details on the solid-phase extraction procedures, FTICR-MS parameter settings, and FTICR-MS data analysis methods are provided in Note S1.7. The possible transformation reactions from the precursors in DFWD to DBC molecules were traced by the mass difference analysis, which is detailed in Section 3.5.

## 3 Results and Discussion

### 3.1 Pyrolysis Characteristics of FWD Components and Releases of Gases

Organic components in FWD dominated the weight loss observed in TG and DTG curves (Table S1). Gaussian deconvolutions of DTG curves (*R*^2^ = 0.98–0.99) were used to fit contributions of different components based on reported DTG peak temperature ranges (Figures 1a and S1, Table S2).^17^ The fitted DTG peak temperatures for lignin (401.62–419.36 °C) were higher than those for cellulose/lipids (330.00–351.73 °C), proteins (310.89–318.91 °C) and hemicellulose (265.00–283.78 °C). This indicated the resistance of lignin to thermal decomposition due to abundant aromatic rings with strong cross-linked properties.^47−49^ Contribution analysis revealed proteins as the largest contributor to decomposition (47%), followed by lignin (21%), hemicellulose (13%), and cellulose/lipids (8%) (Figure 1b and Table S2). This distribution correlated with the highest weight percentage of extractives and the lowest cellulose in FWD (Table S1), indicating that the extractives mainly contained proteins. At the peak temperature, proteins also exhibited the highest weight loss rate (2.46%·min^–1^), followed by cellulose/lipids (1.44%·min^–1^), hemicellulose (1.22%·min^–1^), and lignin (1.06%·min^–1^) (Figure 1b and Table S2). This indicated that proteins dominated the rapid release of gases.^50^ To further understand FWD pyrolytic mechanisms, thermodynamic parameters were calculated (Figure S2 and Table S3). Both *E*_α_ and *A* escalated with increasing α, consistent with the thermodynamic characteristics of FWD reported in other studies.^15,51^ The increase in *E*_α_ indicated the elevated energy barriers for chemical reactions (e.g., gasification) to occur with increasing pyrolysis temperatures. This was attributed to the predominance of lignin, hemicellulose, and proteins in FWD, which elevated *E*_α_ with increasing α while cellulose reduced it.^52^ The Δ*H* values were lower than *E*_α_ by 3.79–5.19 kJ·mol^–1^, indicating easier formation of activated complexes for reactant-to-product conversion.^17^ Positive Δ*G* values indicated energy–intensive reactions during FWD pyrolysis.^53^ Negative Δ*S* values at α ≤ 0.2 implied a reduction in the system disorder at the initial stage,^54^ while positive Δ*S* values at α > 0.2 indicated that the system was far from thermal equilibrium.^54^ Further details regarding thermodynamic analysis of FWD are provided in Note S2.1.

Pyrolytic gases during FWD pyrolysis, including both noncondensable stable gases and VOCs, were identified based on reported FTIR absorbance wavenumbers (Figure 1c, Table S4 and Note S2.2). The absorbance of CO_2_ predominated across the pyrolysis temperatures (Figure S3), with a DTG peak at 343 °C (Figure 1c). Based on the Gaussian deconvolution results, CO_2_/HCNO peaks at 343 °C and NH_3_/HCN peaks at 329 °C can primarily be linked to the decomposition of proteins,^55^ which is consistent with the highest contribution to mass loss in the DTG curve and the fast weight loss rate of proteins. Peaks at 285 and 343 °C, associated with C=O and C–O, mainly originated from the decomposition of hemicellulose, cellulose, and lipids.^47,48,52^ Benzene stretching C=C peaks at 285 °C, 343 °C, and 463 °C, along with CH_4_ emissions at 343 and 466 °C, indicated lignin cracking.^48^ Overall, the percentages of cumulative absorbance for VOCs (O–H, C–O/C–C, C=O, C=C) decreased with increasing pyrolysis temperature, while the percentages of that for noncondensable gases (i.e., CO_2_, CO, CH_4_, HCN, and HCNO) increased at higher temperatures (Figure S3). This probably represented a reduction in VOCs concentration, aligning with the findings of the GC–MS analysis (Figure S4). This implied that the release of noncondensable stable gases at higher pyrolysis temperatures partially resulted from the decomposition of VOCs.^15,51^

### 3.2 Evolutions in Structures and Volatility of Volatile Organic Compounds

The VOCs with a low volatility may overlap with the DBC molecules and a structural illumination of VOCs could provide insights on the sources of DBC molecules. The releases of VOCs intensified within 200–550 °C (Figure 1c). A detailed offline TG-MS analysis of VOCs at 350, 450, and 550 °C revealed their comprehensive structural characteristics (Figure S4). Molecular formulas of the identified VOCs were categorized into four categories based on the presence of nitrogen and oxygen atoms (Tables S5–S7). The CHN and CHNO compounds encompassed amine/amide-N, heterocyclic-N, and cyano-N species, originating from processes such as direct cracking of peptides/heterocyclic nitrogen compounds, amino acid cyclization, and Maillard reaction.^56^ The CHO molecules comprised notable aromatic compounds and their oxygenated alkylated derivatives. The number and intensity of VOCs decreased significantly at 550 °C, consistent with previous study.^15^ This reduction was mainly attributed to a decrease in low-molecular-weight compounds (C1 to C12, acquisition time <11 min) rather than oxidized polycyclic compounds (acquisition time >11 min). Notably, two aromatic products, p-Cresol (C_7_H_8_O) and 6-methyl-1*H*-indole (C_9_H_9_N), were detected across three temperatures. The relative intensity percentage of p-Cresol, an –OCH_3_ rearrangement product of lignin,^57^ decreased from approximately 12% at 350 and 450 °C to about 3% at 550 °C, indicating that higher pyrolysis temperatures reduced –OCH_3_ rearrangement reactions. The intensity of 6-methyl-1*H*-indole, potentially generated from decomposition of proteins,^58^ remained consistent at approximately 4%. This suggests such N-heterocyclic structures endured pyrolysis destruction within 350–550 °C, aligning with observations from algae pyrolysis between 400–600 °C.^58^

The volatility distribution of VOCs covered the log_10_*C*_0_ range of approximately −6 to 12 μg·m^–3^, with most classified as intermediate-volatile compounds (Figure 1d), similar to wood and corncob pyrolysis products.^59^ The presence of nitrogen and oxygen in VOCs lowered their volatility but increased their molecular weight (MW), aligning their distribution closer to the sugar alcohol line (Figure S5).^38^ At 550 °C, VOCs exhibited lower average volatility and higher MW (Figure 1d), consistent with its higher percentages of CHNO and CHO compounds (Figure S6). In general, all identified VOCs were primarily reduced with  ranging from −3 to 0.2 (Figure 1e), comparable to VOCs of semibituminous coal pyrolysis.^19^ The CHNO and CHO compounds with relatively high MW and  located in the log_10_*C*_0_ range of −6 to 2, while VOCs with lower MW and  showed a higher log_10_*C*_0_ of 4–10 (Figure 1e). These results indicated that high MW CHNO and CHO compounds showed a lower volatility. In the  number space, CHO and CHNO compounds mainly fell in the region representing water-soluble organic compounds (Figure 1f). Specifically, volatile CHNO and CHO compounds such as C_21_H_24_N_4_O_5_/C_13_H_15_NO_2_/C_19_H_18_O_6_ at 350 °C (Table S5), C_22_H_27_N_3_O/C_3_H_7_NO_2_/C_14_H_19_NO_4_ at 450 °C (Table S6), and C_12_H_17_NO_2_/C_10_H_12_N_2_O/C_17_H_12_O_3_ at 550 °C (Table S7) could be traced in DBC350, DBC450, and DBC550, respectively, accounting for about 10% of the total VOC intensity at each pyrolysis temperature. It indicated that VOCs with a low volatility exhibited a high potential in forming DBC.

### 3.3 Gas Release Sequences and Evolved Correlation with Functional Groups

**Figure 2:**
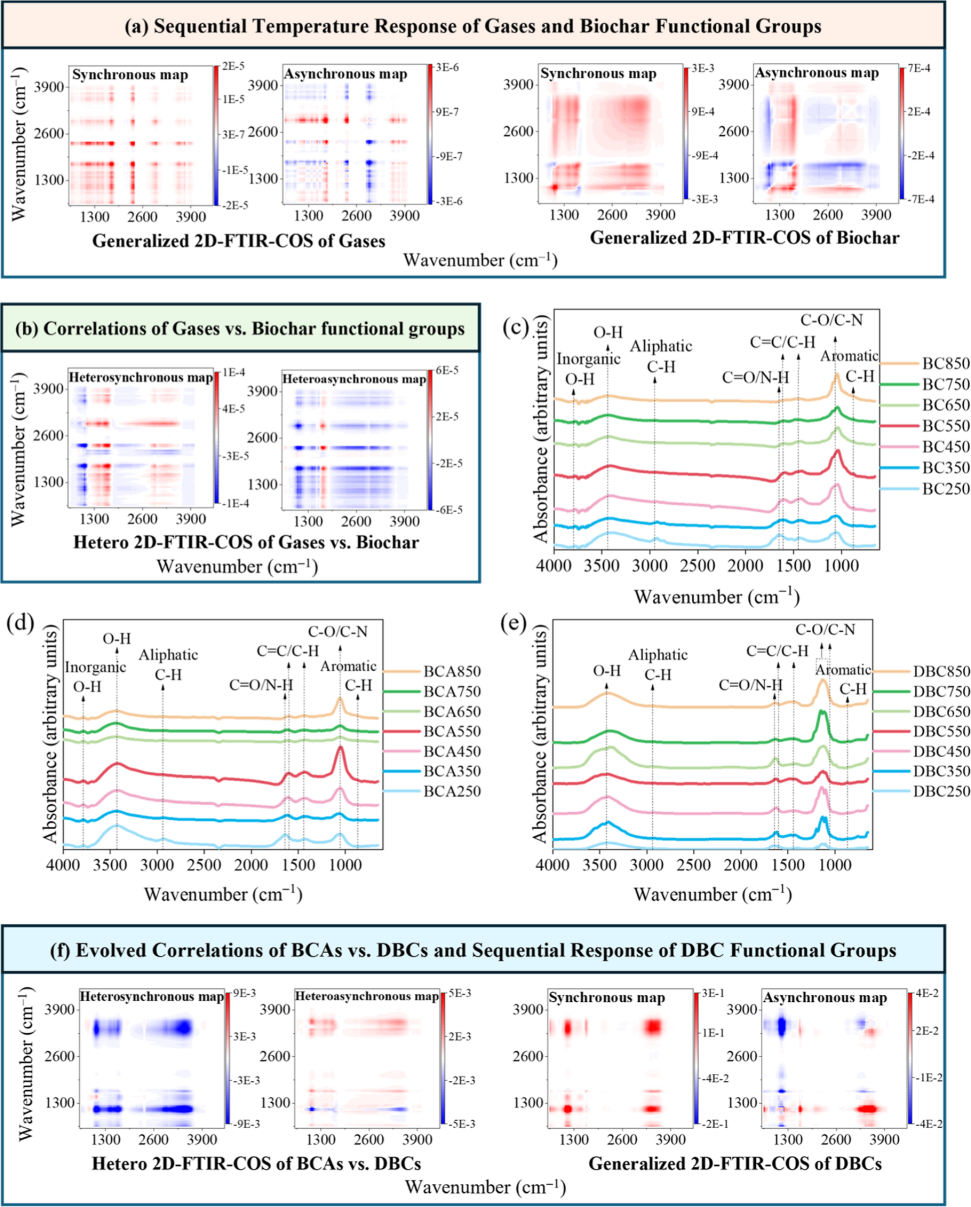
Synchronous and asynchronous maps obtained from (a) generalized 2D-FTIR-COS for gases and biochar functional groups, respectively, (b) hetero-2D-FTIR-COS of gases vs biochar functional groups; FTIR spectra of (c) biochar, (d) BCAs, (e) DBCs; (f) hetero-2D-FTIR-COS for functional groups of BCAs vs DBCs, and generalized 2D-FTIR-COS of DBC functional groups; red and blue colors for auto/cross-peaks in the maps represent the positive and negative correlations; higher intensities of the peaks/regions indicate higher relationships.

Overlaps of gas releases in the temperature profiles made it difficult to discern sequences of gasification reactions. Hence, 2D-TG–FTIR-COS was applied to explore the sequential temperature responses of evolved gases (Figure 2a).^19^ Positive signals dominated the synchronous map of gases, indicating that the spectral changes of most gases proceeded in the same direction as the pyrolysis temperature increased.^41^ The center intensity of the synchronous region at 2336/2336 (*v*1/*v*2) cm^–1^ was notably higher than other centers, indicating that CO_2_ was most susceptible to pyrolysis temperature, consistent with its significant release during FWD pyrolysis. In the asynchronous map of gases, complex positive and negative signs indicated significant heterogeneity in gas evolution. Table S8 summarizes the central regions of interest within the synchronous and asynchronous maps. While some minor noise was observed and excluded from this table, the main trends and correlations identified in the analysis remained consistent and unaffected. According to Noda’s rules, the temperature-dependent sequential changes of gases followed: H_2_O/phenols > ketones/aldehydes/carboxylic acids > ethers > aromatics > CO_2_ > HCN, NH_3_ > CH_4_ > CO (Table S8). Considering the evolved correlations between gases and functional groups (Figure 2b), FWD defunctionalization may undergo stages of dehydration, decarboxylation, deamination/dehydrocyanation, and decarbonylation. Overall, dehydration (−H_2_O) may exhibit the lowest energy barriers for gasification reactions during FWD pyrolysis, while the release of CO requires higher energy input.^47^ The VOC releases, related to devolatilization and/or fragmentation of macromolecular components,^19^ occurred at earlier pyrolysis stages than most noncondensable gases.

To better understand the defunctionalization processes, a generalized 2D-FTIR-COS analysis of biochar functional groups was conducted (Tables S9 and S10). The temperature-dependent evolution of biochar functional groups followed the sequence of aldehyde/ketone/carboxylic groups → amide groups → alcoholic/ether/amine groups, hydrocarbon and aromatic structures → methyl/phenolic –OH groups and heterocyclic nitrogen structures → condensed structures. This elucidated those increasing temperatures resulted in the degradation of functional groups in biochar, forming more condensed structures. A hetero 2D-FTIR-COS analysis was further performed to reveal synergistic correlations of evolved gases vs biochar functional groups, (Figure 2b and Table S11). The sequential temperature responses of gases and biochar functional groups followed: alcoholic/aliphatic ether/amine/amide/ketone/aldehyde/carboxylic groups, and unsaturated structures (biochar) → all gases except CO → Aromatic amine/methyl groups, aromatic/heterocyclic nitrogen structures, and phenolic –OH (biochar). This indicated that more oxygen and nitrogen-rich functional groups were involved in releasing gases, forming hydrocarbon/heterocyclic/aromatic structures driven by high temperatures. Additionally, a sequence of gaseous CH_4_ → unsaturated structures (biochar) → gaseous CO indicated that the release of CH_4_ might form unsaturated structures in biochar, while decompositions of unsaturated structures may cause the release of CO.^15^

Notably, the FTIR spectra of biochar, BCAs, and DBCs showed similar qualitative features, with strong and broad bands at 1100–1800 and 3000–3600 cm^–1^, representing carboxylic, ether, amine, amide, and alcoholic/phenolic functional groups (Figure 2c–e and Note S2.3). This is expected because DBC molecules, taking up <1 wt % of the carbon mass of bulk biochar, were released from char via a surface wash-off process.^20^ The equilibrium of adsorption and desorption during the leaching process of DBC molecules partially explains their qualitative overlap in functional groups with BCAs, despite observed differences in relative intensities. A more detailed spectral analysis revealed that DBC contained a higher proportion of oxygenated and nitrogenous ester/amine functional groups (1250–1150 cm^–1^), distinguishing it from biochar and BCAs (Note S2.3). To reveal the interactions between functional groups in biochar and DBC molecules, a hetero 2D-FTIR-COS analysis of BCAs and DBCs was performed (Figure 2f and Table S12). Negative signals in heterosynchronous maps of BCAs and DBCs indicated that the decreases in BCA functional groups corresponded to the increases in DBC functional groups.^26^ Higher heterosynchronous intensity in certain functional groups signified a stronger leaching ability. These negative signs intensified in regions related to alcoholic/primary amine/phenolic groups, indicating their significant contributions to DBC molecules during the leaching process. Moreover, the nearly opposite heteroasynchronous signals indicated that the decreases in BCA functional groups generally occurred before the corresponding increases in DBC functional groups, showing the dynamic leaching process of compounds from biochar transitioning to DBC.^26^ To further illuminate which functional groups were actively involved in the leaching process and their temperature-dependent changes, a generalized 2D-FTIR-COS analysis of DBC functional groups was performed. The positive synchronous autopeaks indicated that alcoholic/ether/heterocyclic nitrogen/phenolic groups were actively involved in the leaching process and exhibited the same directional change with increasing pyrolysis temperature (Figure 2f). Based on the summarized region centers (Table S13), the temperature-dependent evolutions of DBC functional groups followed: amide groups → phenolic groups → heterocyclic nitrogen structures → acidic/amine/alcoholic groups → ether groups. This demonstrated that the N-rich amide groups in DBC evolved earlier than O-rich functional groups.

### 3.4 Formation and Molecular Features of Dissolved Black Carbon

**Figure 3:**
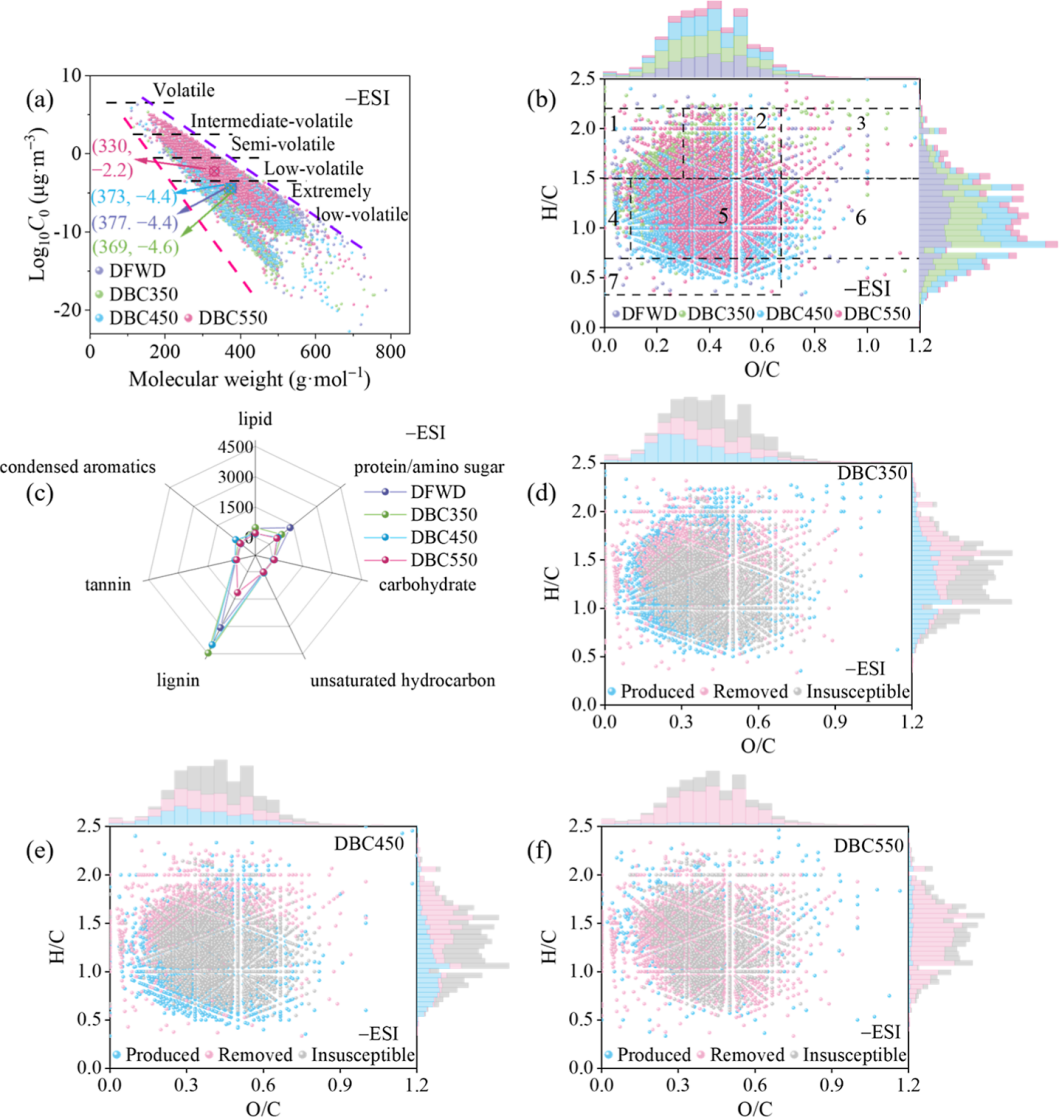
(a) Molecular corridors of log_10_*C*_0_ vs molecular weights for DBC molecules identified under −ESI. Circular crossing markers represent the average values of corridors. Dotted lines represent alkanes C_*n*_H_2*n*+2_ (upper purple line) and sugar alcohols C_*n*_H_2*n*+2_O_*n*_ (lower pink line); (b) van Krevelen diagrams of DFWD and DBC molecules identified under −ESI and (c) the numbers of molecules in different compound classes; (d–f) van Krevelen diagrams of “produced”, “removed” and “insusceptible” formulas identified under −ESI. Regions in the van Krevelen diagram were divided into (1) lipid-like, (2) protein/amino sugar-like, (3) carbohydrate-like, (4) unsaturated hydrocarbon-like, (5) lignin-like, (6) tannin-like and (7) condensed aromatic molecules.

A detailed identification of DBC molecular formulas was performed to scrutinize their temperature-dependent formation mechanisms. The DBC molecules identified under ± ESI modes were below intermediate-volatile (Figures 3a and S7a), and their volatility decreased with increasing MW, O/C ratios, and CHO/CHNO percentages (Figures S8 and S9), aligning with the characteristics of biomass pyrolytic smoke.^60^ The number of formulas assigned to DFWD, DBC350, DBC450, and DBC550 under ± ESI modes were 5548–5805, 6131–6531, 5282–5893, and 2005–4162, respectively. Higher pyrolysis temperatures resulted in reduced molecular diversity, consistent with the reduced VOCs at 550 °C. Unlike VOCs, DBC molecules mainly exhibited low and extremely low volatility, and their volatility increased with higher pyrolysis temperature, attributing to decreasing CHNO/CHNOS percentages (Figure S10).^15^ This suggested that VOCs identified via TG-MS and DBC molecules decoded via FTICR-MS provided complementary bio-oil compound data, with only partial overlap in CHNO information (Tables S5–S7).

Compared to DFWD, DBC350 and DBC450 had fewer labile protein-like compounds but more aromatic lignin-like compounds (Figures 3b,c and S7b,c), consistent with their elevated AI_mod,w_ and DBE_w_ (Table S15). In contrast, DBC550 showed reduction in both protein/lipid-like and lignin-like compounds (Figures 3b,c and S7b,c), with AI_mod,w_ similar to DFWD (Table S15). Compared to DFWD, DBC350 predominated high nitrogen (N4/N5) compounds, while DBC450 and DBC550 favored low nitrogen (N2–N3) and nitrogen-free (N0) compounds (Figure S11). The elevated high-N (N5) DBC350 molecules were mainly oxidized protein-like (−ESI) and low oxygen lignin-like (+ESI) compounds (Figure S11), possibly resulting from the reactions of O-rich acidic groups with gaseous NH_3_/NH_2_* (−ESI) and peptide cyclization (+ESI).^50^ DBC450 had more low-N lignin-like, unsaturated, and condensed compounds (Figure S11), possibly linked to heterocyclic nitrogen formation, as indicated by the significant release of 6-methyl-1*H*-Indole at 350 and 450 °C. DBC550 contained more labile N0 compounds with basic groups than DBC350 and DBC450 (+ESI, Figure S11). Overall, temperature-dependent variations of DBC molecules align with the sequential temperature response of DBC functional groups: Amides (high N molecules) → phenolic and heterocyclic nitrogen structures (highly aromatic molecules) → aliphatic ethers (basic and liable N0 molecules).

Venn analysis categorized formulas in DBC and DFWD samples as “removed”, “produced”, and “insusceptible”. The “produced” molecules, found only in DBC samples, showed clear compositional differences (Figures 3d–f and S7d–f). The “produced” compounds in DBC350 and DBC450 under ±ESI modes were mainly N-containing lignin-like, unsaturated, and condensed compounds with low oxygen content (Figures S12–S15), resulting in elevated AI_mod,w_ and DBE_w_ but decreased O/C_w_ than the “removed” and “insusceptible” ones (Table S16). The increase in AI_mod,w_ but decrease in H/C_w_ and O/C_w_ of “produced” DBC350 and DBC450 molecules (Table S16) probably stemmed from the cyclization/aromatization of proteins/peptides via dehydration and decarboxylation,^14,46^ consistent with the significant release of H_2_O and CO_2_ observed before 450 °C (Figure 1c). In DBC550, abundant N-free highly oxidized labile compounds (H/C > 1.5) formed (Figures 3f, S7f and S12–S15), possibly due to further cracking of lignin-like compounds as accompanied by significant C=C release (Figure 1c).^59^ Most low-N/O heterocyclic and N0 aromatic lignin-like compounds remained “insusceptible” (shared by DFWD and DBC samples, Figures S12–S15), indicating higher stability due to the strong condensed C=C linkage.^60,61^ Overall, the observed changes in N/C_w_, H/C_w_, and O/C_w_ during the formation of DBC molecules correlated well with complex gas releases.

### 3.5 Linking Generation of Dissolved Black Carbon with Release of Pyrolytic Gases

The TG-FTIR analysis identified HNCO, HCN, NH_3_, CH_4_, CO, CO_2_, and H_2_O as the main gas releases during the biochar formation. Significant H_2_, undetectable via TG-FTIR, was also widely observed during the digestate pyrolysis.^51^ The formation of DBC (derived from leaching of biochar) probably involved gas-releasing reactions similar to those observed during biochar pyrolysis. The high solubility of organic carbon in the FWD indicated that DFWD could serve as a precursor to both gas releases and DBC formation (Table S17).^61^ During the DFWD pyrolysis, both intermolecular and intramolecular processes could contribute to gas releases related to DBC formation.^31^ In Figures 3a and S7a, the average masses of DBC samples differed from DFWD by −47 to −4 Da (−ESI) and −24 to +1 Da (+ESI), primarily attributed to the possible loss of functional groups such as −HNCO (−43 Da) and −H_2_O (−18 Da) during intramolecular primary gas-releasing reactions rather than intermolecular reactions. For example, intramolecular dehydration yields a Δ*m*/*z* value of −18 Da. In contrast, intermolecular dehydration tends to produce a larger positive Δ*m*/*z* value, such as near +300 Da, considering the most intense *m*/*z* (near 318) values of DFWD molecules (Figure S16).

To elucidate the connection between gas released and DBC molecule produced, we conducted a mass difference analysis involving nine possible intramolecular primary gas-releasing reactions that correlated with the DBC formation (Table S18). We constructed an inventory based on the formulas in DFWD after the loss of specific functional groups and structural units (e.g., −HNCO, −HCN, −NH_3_, −CH_4_, −CH_2_, −CO, −CO_2_, −H_2_O, and −H_2_). We then searched the “produced” DBC molecules from this inventory to identify the precursor-product pairs, following the approach of previous studies.^30,62^ Our analysis revealed that 47.5–63.2% (−ESI) and 38.7–64.1% (+ESI) of “produced” DBC formulas could be traced back to their precursors, indicating that the primary defunctionalization significantly contributed to the DBC generation. A detailed analysis for the reactive precursors is provided in Note S2.4.

The above mass difference analysis excluded multistage gas releases in the DBC formation, given the −47 to +1 Da mass differences from its DFWD precursor and the observed solubility of DBC molecules. For instance, the mass difference analysis suggested that C_14_H_20_O_5_S in DFWD was converted to C_13_H_20_O_3_S in DBC via a single-stage CO_2_ release, with no further gas-releasing products detected. This single-step release probably preserved the solubility by preventing further loss of oxygen-containing functional groups. In contrast, multistage gas-releasing reactions would infer highly reactive precursors that are prone to radical formation, facilitating gasification over stable DBC formation. Future studies can explore these multistage reactions and potential intermolecular reactions with small gas molecules for further analysis.

**Figure 4:**
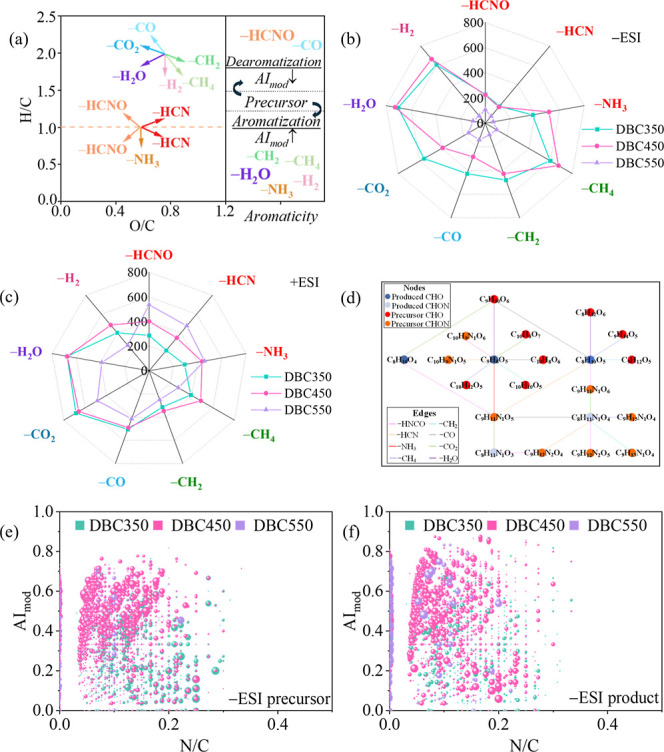
(a) (Left) gas-releasing reaction vectors in the van Krevelen diagram, and the derivation process is shown in Note S2.5; (right) changes of aromaticity via gas-releasing reactions, and the deduction process is demonstrated in Note S2.6; (b,c) numbers of precursor-product pairs based on possible gas-releasing reactions; (d) possible pathways of gas-releasing reactions between the precursors (red dots) and the corresponding products (blue dots) for DBC550 (−ESI); (e,f) AI_mod_ vs N/C diagrams of the precursors and the corresponding products based on 24 possible transformation reactions for the production of DBC molecules (−ESI), with larger dot size representing the larger number of precursors or products involved in the reactions.

To elucidate the impact of primary gas-releasing reactions on the DBC composition and aromaticity, we analyzed the reaction vectors on the van Krevelen diagram and the aromaticity index (AI_mod_) changes from the “precursors” to “products” based on possible gas releases (Figures 4a and S19). Specifically, dehydration and deamination reactions (−H_2_O and −NH_3_) increased formulas in lignin-like and condensed aromatic regions. Decarboxylation and decarbonylation (−CO_2_ and −CO) formed molecules with higher H/C values, while dealkylation and dehydrogenation (−CH_2_, −CH_4_, and −H_2_) produced molecules with higher O/C. Notably, primary releases of H_2_O, NH_3_, CH_2_, CH_4_, or H_2_ all increased the AI_mod_ values of DBC “products” compared to their “precursors”, indicating a rise in aromaticity. Primary releases of CO_2_ or HCN exhibited no change in AI_mod_. Decarbonylation (−CO) and decarboxamidation (−HNCO) reduced AI_mod_ of the “products” compared to the “precursors”. This reduction in aromaticity, such as breakdown of C=C bonds, requires more thermal energy than the aromatization via cracking C–C or C–O bonds, due to the higher dissociation energy of conjugated structures.^63,64^ This aligns with the sequence of “gases except CO → Aromatic groups, unsaturated structures in biochar → CO” (Table S11). This indicates that the gas release sequence helps predict condensation and cracking in both biochar and DBC.

Dehydration (−H_2_O) was the dominant reaction for the “produced” DBC350 and DBC450 molecules under ± ESI modes (Figures 4b,c and S20a), consistent with lower H/C_w_, O/C_w_ and higher AI_mod_ of DBC350 and DBC450 compared to DFWD (Table S15). The dominance of dehydration reactions indicated low energy barriers, aligning with the preferential response of H_2_O as the pyrolysis temperature increased. For DBC550 production, most dehydration products decomposed, the predominant reactions involved were −CO_2_ and −CO under −ESI and −HNCO under +ESI (Figure 4b,c). They did not increase the aromaticity of DBC550 molecules. A more detailed analysis regarding the changes of aromaticity in DBC due to possible gas-releasing reactions is provided in Note S2.6. Additionally, the numbers of −HCNO, −HCN and −NH_3_ increased significantly for DBC550, implying that N-reduction in DBC molecules preferred higher energy input. This was consistent with the sequential temperature responses of gases where the temperature-dependent response of H_2_O preceded over those of NH_3_ and HCN.

In such complex reaction systems, each precursor and product can participate in multiple reactions. For instance, the product C_8_H_10_O_5_ in DBC550 can be derived from six precursors, while the precursor C_9_H_11_N_1_O_5_ can lead to five products (Figure 4d). Under ±ESI modes (Figures 4e,f and S21), the precursors involved in multiple gas-releasing reactions had higher N/C ratios. Conversely, the products involved in multiple reactions had lower N/C ratios. This indicates that nitrogen-rich compounds were involved in the intensified gas-releasing reactions, consistent with the high weight loss rate and contribution portion of proteins (Figure 1). Based on the distributions of precursors and products related to 9 possible transformation reactions, the molecular parameters for DBC350, DBC450, and DBC550 followed the sequence of high N/C and low AI_mod_ → high AI_mod_ → low N/C and low AI_mod_. This echoed the temperature response sequence of amides → phenolic and heterocyclic nitrogen structures → aliphatic ethers. Therefore, the mass difference analysis in this study provided new insights into temperature-dependent formation mechanisms of DBC molecules.

## 4 Environmental Implications

Understanding the mechanism of DBC formation and its association with released gases is critical to predicting interactions with coexisting pollutants and their impact on natural water systems. Our study provides novel insights into the dynamically evolved relationships between gas, biochar and DBC during FWD pyrolysis. Using TG-FTIR-GC/MS data and 2D-COS analysis, we detailed the thermodynamic parameters, product properties and temperature-dependent defunctionalization sequences of FWD. We found that dehydration (−H_2_O) occurred before other defunctionalization reactions, while decarbonylation (−CO) occurred last. As temperature increased, H_2_O and most gases were released before aromatization of biochar, while CO responded after. FTICR-MS, complemented by TG-FTICR-MS, uncovered abundant molecular features of DBC, enhancing our understanding of pyrolysis chemistry and its environmental impacts. Mass difference analysis linked evolved gases with DBC formation, uncovering molecular-level mechanisms and field-relevant implications. Our mathematical derivation of precursor-product molecular relationships showed that the −H_2_O process increased the aromaticity of DBC product, while the −CO and −HCNO processes reduced it. Dehydration (−H_2_O) reactions, with lower energy boundary and priority temperature response, dominated the formation of DBC350 and DBC450 molecules, significantly increasing their aromaticity. At 550 °C, the predominant −CO_2_, −HCNO, and −CO reactions contributed to more labile DBC molecules. Specific DBC compositional changes via primary gas-releasing reactions can be predicted via reaction processing vectors in the van Krevelen diagrams. Our findings imply the predictability and tunability of DBC speciation during biochar fabrication by detecting gas release sequences and modifying pyrolysis conditions (i.e., modifying specific functional groups or adding catalysts to accelerate specific gas emissions).

Insights into the connection between temperature-dependent defunctionalization sequences with DBC formation help in selecting proper pyrolysis conditions to customize or optimize the DBC properties. For instance, denitrogen reactions (i.e., −HCN and −NH_3_), exhibited a delayed temperature-dependent response. The primary release of nitrogen-containing gases for DBC production favored higher energy input and occurred more at 550 °C. Similarly, the primary −CO reactions also preferred higher temperature to produce labile DBC molecules. An elevated pyrolysis temperature to 550 °C is recommended for biochar production from nitrogen-rich feedstock to minimize the release of aromatic/condensed black nitrogen into drinking water sources during scenarios such as rainfall or irrigation. This is because that stable black nitrogen has a high potential to flow into these water sources, serving as precursors to highly toxic nitrogenous disinfection byproducts.^65^ Additionally, wildfires can produce considerable DBC. Our insights into DBC formation mechanisms during biochar production provide references for predicting DBC releases after wildfires via real-time gas evolution monitoring. This study provided limited information on DBC formed via polymerization or macromolecular decomposition. Future research should focus on these aspects using advanced characterization techniques to achieve a more rounded analysis of DBC molecules during biomass pyrolysis. By leveraging these insights, we can improve the predictability and tunability of DBC during biochar production, with significant implications for environmental management and pollution mitigation.

## Supporting Information Available

Analysis models, results, and discussion for the additional important points; FWD compositions and solubility; optimized decomposition parameters, TG/DTG curves, thermodynamic parameters, and TG–FTIR spectra of FWD pyrolysis; volatility distribution, relative intensities of CH, CHN, CHO and CHNO compounds for VOCs; volatility distribution, van Krevelen diagram analysis, relative spectral abundances of CHO, CHOS, CHNO, CHNOS, N- and O-formulas for DFWD and DBC molecules; schemes of possible dehydration reactions; van Krevelen diagrams and molecular parameters of “precursors” related to possible gas-releasing reactions; numbers and number percentages the precursor-product pairs identified under ±ESI; AI_mod_ vs N/C diagrams, van Krevelen diagrams of the precursor-product pairs for DBC molecules identified under +ESI; sequential temperature responses of gases and DBC functional groups, respectively; main functional groups identified in biochar, DBCs, and BCAs; hetero 2D-TG–FTIR/FTIR-COS characteristics and the relationships for sequential temperature responses of gases vs biochar and BCAs vs DBCs, respectively; van Krevelen diagrams of reaction vectors, changes of the AI_mod_, and conditions for an elevated modified aromaticity index from the “precursor” to the “product” (PDF)
