# Supplementary material for: Implications of Pyrolytic Gas Dynamic Evolution on Dissolved Black Carbon Formed During Production of Biochar from Nitrogen-Rich Feedstock
Source: Environ Sci Technol. 2025 Jan 13;59(5):2699–710. doi: 10.1021/acs.est.4c08231 (PMC11823457; doi:10.1021/acs.est.4c08231)
Supplement: Supplementary file 1 — es4c08231_si_001.pdf [file es4c08231_si_001.pdf]

Supporting Information

Implications of Pyrolytic Gas Dynamic Evolution on Dissolved Black Carbon Formed During Production of Biochar from Nitrogen-Rich Feedstock

Xiaoxiao Zhang [a], Zibo Xu [b], Yuqing Sun [c], Sanjay K Mohanty [d], Hanwu Lei [e], Eakalak Khan [f], Daniel C.W. Tsang [b, *]

[a] Department of Civil and Environmental Engineering, The Hong Kong Polytechnic University, Hung Hom, Kowloon, Hong Kong 999077, China

[b] Department of Civil and Environmental Engineering, The Hong Kong University of Science and Technology, Hong Kong 999077, China

[c] School of Agriculture, Sun Yat-sen University, Shenzhen 518107, Guangdong, China

[d] Department of Civil and Environmental Engineering. University of California Los Angeles, California 90095, United States

[e] Department of Biological Systems Engineering, Washington State University, Richland, Washington 99354-1671, United States

[f] Civil and Environmental Engineering and Construction Department, University of Nevada, Las Vegas, Nevada 89154-4015, United States

This *Supporting Information* file has 72 pages including 21 figures and 22 tables.

*Corresponding author: Daniel C.W. Tsang

Email: cedan@ust.hk

Table of Contents

Note S1. Materials and Methods
Note S1.1 Food Waste Digestate Preparation and Component Determination
Note S1.2 Gaussian Model for the Derivative Thermogravimetric Curves
Note S1.3 Pyrolysis Kinetic and Thermodynamic Calculations
Note S1.4 Integral Method and Parameter Calculation of Gaseous Compounds
Note S1.5 FTIR Measurements of Biochar and Dissolved Black Carbon
Note S1.6 Two-Dimensional Correlation Spectroscopy Theory and Interpretations
Note S1.7 Extraction, FTICR-MS Parameter Settings and Data Analyses

Note S2. Results and Discussion
Note S2.1 Thermodynamic Characteristics of Food Waste Digestate Pyrolysis
Note S2.2 Identification of Gaseous Products via TG-FTIR
Note S2.3 Identification of Functional Groups in Biochar, DBCs and BCAs
Note S2.4 Precursors Susceptible to Possible Primary Gas-releasing Reactions
Note S2.5 Gas-Releasing Reaction Directions in van Krevelen Diagrams
Note S2.6 Changes of Aromaticity via Gas-Releasing Reactions

Figure Captions

Figure S1. Optimized decomposition processes for components of FWD pyrolysis at four heating rates based on the Gaussian model.

Figure S2. (a) TG and DTG curves and (b) Arrhenius plots at five heating rates of FWD pyrolysis; plots of (c) $E\alpha$ and R^2, (d) ΔH, (e) ΔG and (f) ΔS values as a function of the conversion rate.

Figure S3. (a) Three-dimensional FTIR, (b) FTIR absorbances at DTG peak temperatures, (c) cumulative FTIR absorbances at different temperature ranges, and (d) cumulative FTIR absorbance at specific temperatures for gaseous products released during FWD pyrolysis.

Figure S4. GC-MS total ion chromatograms at pyrolysis temperatures of (a) 350 ℃, (b) 450 ℃, and (c) 550 ℃. Red "×" marks the column bleeding. The illustration depicts the relative abundances of volatile organic products within different GC-MS acquisition time.

Figure S5. Volatility distribution of volatile organic compounds identified at (a) 350 ℃, (b) 450 ℃, (c) 550 ℃ from FWD pyrolysis and (d) percentages of compounds with varying volatility. Upper (purple) and lower (red) boundaries in Log_{10} C_0 vs MW plot represent linear alkanes and sugar alcohols, respectively.[1] The circular crossing markers show the mean values.

Figure S6. Relative TG-GC-MS intensities of pyrolytic volatile organic "CH", "CHN", "CHO" and "CHNO" compounds released at 350 ℃ (Table S5), 450 ℃ (Table S6) and 550 ℃ (Table S7). The classification of volatile organic compounds was based on the presence of nitrogen and oxygen atoms.

Figure S7. (a) Molecular corridors of $Log_{10}C_0$ vs. molecular weight for DBC molecules identified under +ESI. Circular crossing markers represent the average values of corridors. Dotted lines represent alkanes C_nH_{2n+2} (upper purple line) and sugar alcohols $C_nH_{2n+2}O_n$ (lower pink line); (b) van Krevelen diagrams of DFWD and DBC molecules identified under +ESI and (c) the numbers of molecules in different compound classes; (d)–(f) van Krevelen diagrams of "produced", "removed" and "insusceptible" formulae identified under +ESI. Numbers in different regions of the van Krevelen diagram represent different types of molecules as demonstrated in Note S1.7.

Figure S8. Molecular corridors of $Log_{10}C_0$ vs. molecular weight for DBC formulae identified under –ESI and the signals represent the O/C values for each formula. Circular crossing markers represent the average values of corridors. Dotted lines represent representing alkanes C_nH_{2n+2} (upper purple line) and sugar alcohols $C_nH_{2n+2}O_n$ (lower pink line).[1] Numbers in different regions represent volatility (Note S1.4).

Figure S9. Molecular corridors of $Log_{10}C_0$ vs. molecular weight for DBC formulae identified under +ESI and the signals represent the O/C values for each formula. Circular crossing markers represent the average values of corridors. Dotted lines represent representing alkanes C_nH_{2n+2} (upper purple line) and sugar alcohols $C_nH_{2n+2}O_n$ (lower pink line). Numbers in different regions represent volatility (Note S1.4).

Figure S10. Relative FTICR-MS spectral abundances of CHO, CHOS, CHON, CHONS, and "Others" (mainly P-containing compounds) for DFWD and DBC samples.

Figure S11. (a) Numbers and relative percentages of different types of molecules classified by van Krevelen diagrams, and (b) the relative spectral abundances of N- and O-formulae identified in DFWD, DBC350, DBC450 and DBC550.

Figure S12. Numbers and percentages of different types of molecules classified via van Krevelen diagram for the "produced", "removed" and "insusceptible" molecules categorized under –ESI.

Figure S13. Numbers and percentages of different types of molecules classified via van Krevelen diagram for the "produced", "removed" and "insusceptible" molecules categorized under +ESI.

Figure S14. Changes of the relative spectral abundances of N- and O-formulae identified under −ESI during production of (a) DBC350, (b) DBC450, and (d) DBC550.

Figure S15. Changes of the relative spectral abundances of N- and O-formulae identified under +ESI during production of (a) DBC350, (b) DBC450, and (d) DBC550.

Figure S16. Schemes of possible (a) intramolecular and (b) intermolecular dehydration reactions of DFWD involved in DBC formation; (c) the negative ion mode FTICR-MS spectrum of DFWD.

Figure S17. Van Krevelen diagrams showing the distribution of "precursors" related to possible gas-releasing reactions for (a) DBC350, (b) DBC450, and (c) DBC550 identified under −ESI. Numbers in different regions of the van Krevelen diagram represent different types of molecules as demonstrated in Note S1.7.

Figure S18. Van Krevelen diagrams showing the distribution of "precursors" related to possible gas-releasing reactions for (a) DBC350, (b) DBC450, and (c) DBC550 identified under +ESI. Numbers in different regions of the van Krevelen diagram represent different types of molecules as demonstrated in Note S1.7.

Figure S19. (a) Van Krevelen diagrams showing the distributions and reaction vectors from the "precursor" to the "product" due to possible gas-releasing reactions, and (b) changes of the AI_{mod} from the "precursor" to the "product"; reacion vectors were drawn based on analysis in Note S2.5; changes of AI_{mod} (↓: reduced, = unchanged, ↑: increased) were drawn based on the analysis in Note S2.6.

Figure S20. (a) Numbers and (b)–(d) number percentages the precursor-product pairs for DBC formulae identified under ±ESI based on possible gas-releasing reactions.

Figure S21. (a)–(b) AI_{mod} vs. N/C diagrams of precursor-product pairs for the production of DBC molecules (+ESI) and (c)–(f) van Krevelen diagrams of the precursor-product pairs for DBC molecules identified under ±ESI based on the possible gas-releasing reactions. The larger dot size represents the larger number of precursors/products involved in the reactions. Regions in the van Krevelen diagram were divided based on the Note S1.7.

Table Captions

Table S1. Compositions and their weight percentages of the FWD.
Table S2. Optimized decomposition parameters of FWD pyrolysis at different heating rates.
Table S3. Thermodynamic parameters of the FWD pyrolysis derived from the Flynn-Wall-Ozawa method at different conversion rates under the heating rate of 10 ℃·min^{-1}.
Table S4. Main functional groups of gaseous products identified using TG-FTIR.
Table S5. Main volatile organic compounds identified at pyrolysis temperature of 350 ℃ by GC-MS coupled NIST library (volatile organic compounds shared by 350, 450, and 550 °C, as well as those common to DBC350, DBC450, or DBC550, are highlighted in bold).
Table S6. Main volatile organic compounds identified at pyrolysis temperature of 450 ℃ by GC-MS coupled NIST library (volatile organic compounds shared by 350, 450, and 550 °C, as well as those common to DBC350, DBC450, or DBC550, are highlighted in bold).
Table S7. Main volatile organic compounds identified at pyrolysis temperature of 550 ℃ by GC-MS coupled NIST library (volatile organic compounds shared by 350, 450, and 550 °C, as well as those common to DBC350, DBC450, or DBC550, are highlighted in bold).
Table S8. Generalized 2D-TG-FTIR-COS characteristics and the sequential temperature responses of gaseous products during FWD pyrolysis.
Table S9. Main functional groups identified in FTIR spectra of biochar, DBCs, and BCAs.
Table S10. Generalized 2D-FTIR-COS characteristics and the sequential temperature responses of biochar functional groups.
Table S11. Hetero 2D-TG-FTIR/FTIR-COS characteristics and the relationships between the sequential temperature responses of gases and biochar functional groups.
Table S12. Hetero 2D-TG-FTIR/FTIR-COS characteristics and the relationships between the sequential temperature responses of functional groups in BCAs and DBCs.
Table S13. Generalized 2D-FTIR-COS characteristics and the sequential temperature responses of DBC functional groups.
Table S14. n_C^0 and b values of saturation mass concentration for different compound classes.
Table S15. Intensity-weighted molecular parameters of DFWD and DBC samples.
Table S16. Intensity-weighted molecular parameters of the “produced”, “removed” and “insusceptible” molecules.
Table S17. Ultimate and organic carbon analysis performed on bulk FWD (wt.% dry basis).
Table S18. Summary of the transformation reactions related to possible primary gas releases.
Table S19. Intensity-weighted molecular parameters of “precursors” involved in possible gas-releasing reactions during DBC production (–ESI).
Table S20. Intensity-weighted molecular parameters of “precursors” involved in possible gas-releasing reactions during DBC production (+ESI).
Table S21. Reaction vectors from the “precursor” to the “product” in the van Krevelen diagram based on the possible gas-releasing reactions.
Table S22. Conditions for an elevated modified aromaticity index (AI_{mod}) from the initial “precursor” to the final “product” based on the possible gas-releasing reactions.

References

Note S1. Materials and Methods

Note S1.1 Food Waste Digestate Preparation and Component Determination

The food waste digestate (FWD), collected from O•PARK1 in Hong Kong, was oven-dried at 105 °C until reaching a constant weight to ensure microbial inactivation and moisture removal. The dried FWD was then finely crushed to approximately 1 mm in diameter and sealed for pyrolysis experiments. The components of FWD were analyzed using the van Soest washing method with an ANKOM2200 fiber analyzer.[2] Approximately 1 g of each substrate was boiled in neutral detergents at 100 °C for 2 h. After boiling, the mixture was filtered using a glass microfiber filter (0.45 μm) to collect hemicelluloses, celluloses, lignin, and ash. The hot-soluble components, termed extractives, included proteins, lipids, starches, and sugars. The insoluble mixture was dried to a constant weight and reconstituted in 100 mL of acid detergent, then heated at 100 °C in a water bath for 2 hours for further extraction. Filtration of this new mixture captured the cellulose, lignin, and ash components, while the filtrate was identified as the hemicellulose fraction. Next, 72% (v/v) H_2SO_4 was added to the residue and agitated at 200 rpm for 1 h. Subsequent filtration yielded a mixture of lignin and ash, while the filtrate was identified as the cellulose fraction. The ash content in the residue was quantified using a muffle furnace at 500°C for 5 h. The lignin content in the FWD sample was then determined. This study focused on organic matter proportions, excluding ash content for the assessments.

The FWD elemental analysis (C, H, N, S; wt.% dry basis) was performed using an elemental analyzer (Vario EL cube, Germany) with random duplications. The solubility of organic carbon in FWD was assessed by measuring the total organic carbon (TOC) in bulk FWD and the water-extractable organic carbon (WEOC) in the dissolved food waste digestate (DFWD). Both TOC and WEOC levels were analyzed using a Shimadzu TOC analyzer.

Note S1.2 Gaussian Model for the Derivative Thermogravimetric Curves

A Gaussian model was utilized to identify and quantify the contributions of distinct peaks in the derivative thermogravimetric (DTG) spectra to the overall weight loss during FWD pyrolysis in the thermal gravimetric (TG) analyzer, processed with PeakFit 4.0 Software employing the second derivative fitting algorithm.[3] Assuming that the experimental spectrum $E_{\text{exp}}(x)$ at a spectral variable x is superimposed by individual characteristic peak $S_i(x)$ (i =1, 2, ..., n).[5] The correlation between $E_{\text{exp}}(x)$ and $S_i(x)$ is:

$$E_{\text{exp}}(x) = \sum S_i(x) \quad \text{(S1)}$$

A Gauss-Lorentz amplitude function was employed to simulate each characteristic peak:

$$S_i(x) = a_3 G_i(x) + (1 - a_3) L_i(x) \quad \text{(S2)}$$

The Gauss and Lorentz amplitude expressions are denoted as Gi (x) and Li (x), respectively:

$$G_i(x) = a_{0i} \exp\left[-\frac{(x - a_{1i})^2}{2a_{2i}^2} \right] \quad \text{(S3)}$$

$$L_i(x) = \frac{a_{0i}}{1 + [\frac{(x - a_{1i})}{a_{2i}}]^2} \quad \text{(S4)}$$

From the analysis of DTG curves (Note S2.1), each DTG curve can be fitted by 4 peaks representing decompositions of different components. The weight loss rate (y value) at time of the fitted peak temperature (t_{p}) and the contribution of each partial process to the overall weight loss were modified following a previous study:[3]

$$\text{weight loss rate} = \frac{\mathrm{d}\alpha}{\mathrm{d}t_{\text{p}}} \quad \text{(S5)}$$

$$\text{Contribution to mass loss} = (\alpha_{T_2} - \alpha_{T_1}) \times \frac{A_i}{\sum A_i} = \frac{m_{T_1} - m_{T_2}}{m_0 - m_f} \times \frac{A_i}{\sum A_i} \quad \text{(S6)}$$

where α_{T_1} and α_{T_2} are the conversion rates at T_1 (100 °C) and T_2 (800 °C), respectively. The A_i represents the area of each component with the subscript i numbering components (from 1 to 4). The m_{T_1} , m_{T_2} , m_{T_0} and m_{T_f} represent the actual masses of FWD at 100 °C, 800 °C, initial, and final pyrolysis temperatures, respectively.

Note S1.3 Pyrolysis Kinetic and Thermodynamic Calculations

The apparent activation energy (E_α) at a conversion rate (α) approximates the true activation energy, representing the energy barrier for a chemical reaction.[4] The thermal decomposition of FWD was analyzed using the Arrhenius equation:

$$\mathrm{d}\alpha / \mathrm{d}T = \frac{A}{\beta}\exp(-\frac{E_\alpha}{RT})\,\mathrm{f}(\alpha) \quad \text{(S7)}$$

$$\alpha = \frac{m_0 - m_t}{m_0 - m_f} \quad \text{(S8)}$$

where m_0, m_t and m_f represent the initial, instant, and final masses of the FWD during TG testing, respectively, g; T is the instant temperature, K; A is the apparent pre-exponential factor, s^{-1}; β is the heating rate, $K\cdot s^{-1}$; f(α) is the conversion dependence function; R is the ideal gas constant, 8.314 $J\cdot mol^{-1}\cdot K^{-1}$.

The initial reaction conditions are $\alpha = 0$, $T = T_0$, and the integral form is:

$$\mathrm{g}(\alpha) = \int_0^\alpha \frac{1}{\mathrm{f}(\alpha)}\mathrm{d}\alpha = \frac{A}{\beta}\int_{T_0}^{T}\exp(-\frac{E_\alpha}{RT})\,\mathrm{d}T = \frac{AE_\alpha}{\beta R}\mathrm{p}(\frac{E_\alpha}{RT}) \quad \text{(S9)}$$

$$\mathrm{p}\left(\frac{E_\alpha}{RT}\right) = \frac{\exp(-\frac{E_\alpha}{RT})}{\left(\frac{E_\alpha}{RT}\right)^2}\left(1 + \frac{2!}{-\frac{E_\alpha}{RT}} + \frac{3!}{-\left(\frac{E_\alpha}{RT}\right)^2} + \frac{4!}{-\left(\frac{E_\alpha}{RT}\right)^3} + \dots\right) \quad \text{(S10)}$$

A free model approach, the Flynn-Wall-Ozawa method, is expressed as:[5]

$$\mathrm{Ln}(\beta) = -\frac{E_\alpha}{RT} + \frac{AE_\alpha}{R\mathrm{g}(\alpha)} \quad \text{(S11)}$$

By solving the slope of the linear relationship between ln(β) and 1/T, E_α can be determined.

The pre-exponential factor (A) in the Arrhenius equation can be determined using the DTG peak temperature (T_{peak}):

$$A = \frac{\beta E_\alpha \exp(E_\alpha / RT_{\mathrm{peak}})}{RT_{\mathrm{peak}}^2} \quad \text{(S12)}$$

Thermodynamic parameters including changes in enthalpy (ΔH), Gibb's free energy (ΔG) and entropy (ΔS) can be calculated as:[6]

$$\Delta H = E_\alpha - RT \quad \text{(S13)}$$

$$\Delta G = E_\alpha + RT_{\mathrm{peak}}\ln(\frac{K_B T_{\mathrm{peak}}}{hA}) \quad \text{(S14)}$$

$$\Delta S = \frac{\Delta H - \Delta G}{T_{\mathrm{peak}}} \quad \text{(S15)}$$

where K_B is the Boltzmann constant (1.381×10^{-23} $J\cdot K^{-1}$), h is Plank's constant (6.626×10^{-34} J·s).

Note S1.4 Integral Method and Parameter Calculation of Gaseous Compounds

In Thermogravimetric-Fourier Transform Infrared-Mass Spectrometer (TG-FTIR-MS) analysis, approximately 22 mg FWD was heated. The TG−FTIR-MS experiments were performed twice, eliminating background signals without FWD. Gaseous products were determined by their characteristic FTIR bands (Table S4). The collected FTIR spectra were smoothed/baseline-corrected using OMNIC software before analysis. The area under the FTIR absorbance curve was reported to be directly proportional to the concentration of gas released during pyrolysis.[7] Thus, the cumulative gas emission was measured by integrating the FTIR absorbance (A) over time (t), expressed as the cumulative absorbance $I_{A,t}$:

$$I_{A,t} = \int_0^t A \, \mathrm{d}t \tag{S16}$$

Organic compounds (OCs) at key DTG/FTIR peak temperatures (350, 450, and 550 °C) were swept directly to the GC-MS system for structural identification of prominent ions with m/z = 30−300 using the Automated Mass Spectral Deconvolution and Identification System (AMDIS, USA) with mass spectra libraries from Wiley, NIST/EPA/NIH. Compounds with matching scores above 50% and signal–to–noise ratios higher than 10:1 were considered correctly identified. The saturation mass concentration (C_0, μg·m^{-3}) of volatile OCs can be calculated as:

$$C_0 = \frac{M_0 10^6 \varepsilon_0 p_0}{760RT} \tag{S17}$$

where M_0, ξ_0, p_0, R, and T are the molar mass (g·mol^{-1}) of the species, the species activity coefficient in the condensed phase (assumed to be 1), the subcooled liquid saturation vapor pressure of pure compound at 298 K (mm Hg), the ideal gas constant (8.205×10^{-5} atm·K^{-1}·mol^{-1}·m^3), and temperature (K), respectively. The saturation vapor pressure (p_0) of volatile species identified by GC-MS was estimated by the Estimation Programs Interface Suite software (version 4.1), based on an average of the Antoine and the modified Grain estimates. The C_0 and Log$_{10}$ C_0 reflected the volatility:

1. Volatile OCs ($C_0 > 3 \times 10^6$ μg m^{-3}, Log$_{10}C_0$ > 6.48),
2. Intermediate volatility OCs ($300 < C_0 < 3 \times 10^6$ μg m^{-3}, 2.48 < Log$_{10}C_0$ < 6.48),
3. Semi volatile OCs ($0.3 < C_0 < 300$ μg m^{-3}, –0.52 < Log$_{10}C_0$ < 2.48),
4. Low-volatile OCs ($3 \times 10^{-4} < C_0 < 0.3$ μg·m^{-3}, –3.52 < Log$_{10}C_0$ < –0.52), and
5. Extremely low-volatile OCs ($C_0 < 3 \times 10^{-4}$ μg m^{-3}, Log$_{10}C_0$ < –3.52).

Note S1.5 FTIR Measurements of Biochar and Dissolved Black Carbon

Biochar and biochar after leaching (BCAs) samples, produced at different pyrolysis temperatures, were dried at 105 ℃ overnight to remove moistures. These samples were finely ground with KBr powder (FTIR grade, Sigma Aldrich) for tableting. For DFWD and dissolved black carbon samples (DBCs), 10 mL of each solution was mixed with 100 mg of KBr, freeze-dried, and ground for tableting. All tablets were characterized using a Thermo Nicolet FTIR Nexus spectrometer. The FTIR spectra were recorded with wavenumbers from 4000 to 600 cm^{-1} and a resolution of 4 cm^{-1}. Smoothing and baseline correction were performed for the FTIR data using OMNIC software before further analysis. To minimize noise in FTIR signals, we averaged multiple scans to enhance the signal-to-noise ratio. The analysis was conducted on multiple samples to assess the reproducibility of the results. Consistent 2D-FTIR-COS patterns observed across replicate samples reflected the reliability and reproducibility of the findings.

Note S1.6 Two-Dimensional Correlation Spectroscopy Theory and Interpretations

Since its inception by Noda in 1990,[8] two-dimensional correlation spectroscopy (2D-COS) has been a key chemometric method for analyzing complex spectra.[9] 2D-COS can reveal subtle details within spectral variations that may be missed in one-dimensional spectra. By extending spectra into a second dimension, it enhances spectral resolution, simplifies complex overlaps, and identifies the directions and sequences of latent variations under varying conditions such as temperature, pH, and pressure.[8,10]

Generalized and heterogeneous 2D-COS analysis offers nuanced insights into subtle changes not easily discernible with one-dimensional spectroscopy.[8] The dynamic changes $\tilde{y}(x_i, t_k)$ and spectral variations $y(x_i, t_k)$ in generalized 2D-COS analysis are determined by both the spectral variable (x_i) and an external variable (t_k). The averaged spectra $\bar{y}(x_i)$ as a function of the stationary t variable and $y(x_i, t_k)$ are considered. The synchronous and asynchronous maps are calculated using the following equations:[10]

$$\tilde{y}(x_i,t_k)=\begin{cases} y(x_i,t_k)-\bar{y}(x_i) & for\ t_{min}\le t_k\le t_{\max} \\ 0 & \text{otherwise}\end{cases} \quad \text{(S18)}$$

$$\bar{y}(x_i)=\frac{1}{t_{\max}-t_{\min}}\sum_{t_{\min}}^{t_{\max}} y(x_i,t_k) \quad \text{(S19)}$$

$$\varphi(x_1,x_2)=\frac{1}{t_{\max}-t_{\min}}\sum_{t}^{t_{\max}} \tilde{y}(x_1,t_k)\cdot\tilde{y}(x_2,t_k) \quad \text{(S20)}$$

$$\Psi(x_1,x_2)=\frac{1}{t_{\max}-t_{\min}}\sum_{t_{\min}}^{t_{\max}} \tilde{y}(x_1,t_k)\cdot\sum_{t_{\min}}^{t_{\max}} N_{ik}\tilde{y}(x_2,t_k) \quad \text{(S21)}$$

where $N_{ik}=\begin{cases} 0 & \text{if } i=k \\ \dfrac{1}{\pi(k-i)} & \text{otherwise}\end{cases}$ facilitates the calculation of these maps for data in row j and column k.[8]

Heterosynchronous and heteroasynchronous maps from hetero 2D-COS analysis are obtained by integrating two sets of complementary dynamic spectral signals [$\tilde{A}(x_i, t_k)$ and $\tilde{B}(y_i, t_k)$] as follows:[11]

$$\varphi(x_1,y_2)=\frac{1}{m-1}\sum_{k=1}^{m}\tilde{A}(x_1,t_k)\cdot\tilde{B}(y_2,t_k) \quad \text{(S22)}$$

$$\Psi(x_1,y_2)=\frac{1}{m-1}\sum_{k=1}^{m}\tilde{A}(x_1,t_k)\cdot\sum_{k=1}^{m}N_{ik}\tilde{B}(y_2,t_k) \quad \text{(S23)}$$

Previous studies have established principles for interpreting spectral coordinates, intensities, and correlation peaks or regions in synchronous and asynchronous maps.[8,10,12] Auto-peaks and symmetrical cross-peaks in the generalized synchronous map appear at the main diagonal and off-diagonal positions, respectively. Auto-peaks are sensitive to changes in spectral intensity, while cross-peaks represent coordinated spectral intensity changes between two variables ($v1$ and $v2$). The positive or negative signs of cross-peaks indicate the direction of spectral intensity changes for the variables. Asynchronous maps depict sequential variations induced by perturbations, showing only cross-peaks at off-diagonal positions. Consistent signals between synchronous and asynchronous maps indicate that the predominant change in spectral intensity at the $v1$ variable appears before that at the $v2$ variable along the perturbation variable axis. Differing signs indicate reversed reaction orders. A zero signal in the synchronous map signifies an indeterminate sequential order of intensity variations.

Note S1.7 Extraction, FTICR-MS Parameter Settings and Data Analyses

Solid-phase extractions were conducted for the extractions of DFWD and DBCs by using the Bond Elut PPL absorbents (6 mL, Agilent Technologies, Folsom, CA). Prior to solid-phase extractions, the TOC concentrations of DBCs were determined using a TOC analyzer (Shimadzu Corporation). This study targeted 0.60 mg TOC for the solid-phase extraction to avoid overloading of the absorbent.[13] The pH of DFWD and DBCs were adjusted to 2 with formic acid rather than HCl before solid-phase extraction to avoid the interference of CHOCl on CHOP assignments and enhance the compatibility with subsequent MS determination.[14,15] In the solid-phase extraction process, cartridges were rinsed with two cartridge volumes of methanol followed by two volumes of 0.01 mol·L^{-1} HCl. The acidic DFWD and DBCs (pH = 2) were then passed through the PPL cartridges at controlled flow rates of < 5 mL·min^{-1}. Before eluting the fractions of DFWD and DBCs, two cartridge volumes (12 mL) of 0.01 M HCl flowed through the column to eliminate residual salts. The sorbents were dried under a gentle stream of nitrogen for 5 min, and extracts were eluted with two volumes of methanol. The eluates were concentrated to approximately the final 1 mL using nitrogen gas and stored at –20 ℃ for analysis. Considering that PPL can retain ~40% of TOC,[13] the enriched TOC concentration of extracts were about 0.24 mg·mL^{-1}.

The extracts of DFWD and DBCs were injected into a Bruker SolariX Fourier transform ion cyclotron resonance mass spectrometry (FTICR-MS) instrument with electrospray ionization in negative and positive modes (–ESI and +ESI), respectively. Key parameters included continuous injection at 120 μL·h^{-1}, ±4.0 kV capillary entrance voltage, 0.08 s ion accumulation time, data collection range of *m/z* 100–800, acquisition of 4 million 32-bit data points, and 300-fold time-domain signal averaging to enhance the signal-to-noise ratio. The instrument was calibrated by a 10 mM sodium formate solution before infusion, followed by the internal standard calibration using a known homologous series of natural organic matter, maintaining mass errors of formulae assignments < 1 ppm.[16] To minimize incorrect assignments, criteria such as isotope patterns,[17,18] N-rules,[19] the homologue rule,[20] and the minimum number of heteroatoms rule,[21,22] were considered to select the correct molecular formulae. Additionally, formulae with $0.2 \leq H/C \leq 2.3$ and $0 \leq O/C \leq 1.2$ were retained.[23] An in-house Python program was used to match and filter the peak list of the molecular formulae, following the criteria outlined above and the primary Python code is as follows:

```
import numpy as np
import pandas as pd

def get_ele_composition(massmatch, ecsList, masslist_sample, oeles):
    meas_mass = masslist_sample.loc[:, ['meas.m/z', 'intens', 'S/N']]
    meas_mass['mrn1'] = meas_mass.index
    massmatch = pd.merge(massmatch, meas_mass, on='mrn1', how='left')

    ecsList['mrn2'] = ecsList.index
    massmatch = pd.merge(massmatch, ecsList, on='mrn2', how='left')

    if 'Na_x' in massmatch.columns:
        massmatch = massmatch.rename(columns={'Na_x': 'Na'})
        massmatch = massmatch.drop('Na_y', axis=1)
        massmatch['Na'] = massmatch['Na'].fillna(0)

    massmatch = massmatch[['meas.m/z', 'neu.m/z', 'mrn1', 'theor.m/z', 'mrn2', 'err.ppm',
```

```
                        'intens', 'S/N', 'C', 'H', 'N', 'O', '13C', 'S', 'P', 'Cl', 'Br', 'I', 'Na']]
    massmatch['H'] = massmatch['H'] - np.sum(massmatch[['Cl', 'Br', 'I']], axis=1)
    massmatch['H'] = massmatch['H'].astype('int')
    massmatch[['Cl', 'Br', 'I']] = massmatch[['Cl', 'Br', 'I']].astype('int')

    for i in massmatch.columns:
        if (massmatch[i] == 0).all() and i != 'Na':
            massmatch = massmatch.drop(i, axis=1)

    cols = list(massmatch.columns[8:])
    massmatch['formula'] = ''
    for i in cols:
        massmatch.loc[massmatch[i] > 1, 'formula'] = massmatch['formula'] + i +
massmatch.loc[massmatch[i] > 1, i].astype('str')
        if (massmatch[i] == 1).any():
            massmatch.loc[massmatch[i] == 1, 'formula'] = massmatch['formula'] + i

    massmatch['CH4-O'] = ''
    massmatch['C'] += 1
    massmatch['H'] += 4
    massmatch['O'] -= 1

    for i in cols:
        if i in massmatch.columns:
            massmatch.loc[massmatch[i] > 1, 'CH4-O'] = massmatch['CH4-O'] + i +
massmatch.loc[massmatch[i] > 1, i].astype('str')
            if (massmatch[i] == 1).any():
                massmatch.loc[massmatch[i] == 1, 'CH4-O'] = massmatch['CH4-O'] + i
            if (massmatch[i] < 0).any():
                massmatch.loc[massmatch[i] < 0, 'CH4-O'] = massmatch['CH4-O'] + i +
massmatch.loc[massmatch[i] < 0, i].astype('str')

    massmatch['C'] -= 1
    massmatch['H'] -= 4
    massmatch['O'] += 1

    massmatch = massmatch.drop(['mrn1', 'mrn2'], axis=1)
    col_name = massmatch.columns.tolist()
    col_name.insert(col_name.index('neu.m/z'), 'formula')
    col_name.insert(col_name.index('neu.m/z'), 'CH4-O')
    massmatch = massmatch.reindex(columns=col_name[:-2])

    return massmatch

def isopeaks_screen(data, massmatch, isomasses, atommasses, paras, err_ppm=1, noise_T=0,
get_cal_comps=1):
    if get_cal_comps == 0:
        L = []
        for i in ['C', 'N', 'O', 'S', 'Cl', 'Br']:
            if i not in massmatch.columns:
```

```
            for j in isomasses.index:
                if i in j:
                    L.append(j)
        isomasses = isomasses.drop(L, axis=0)

    if get_cal_comps == 1:
        L = ['13C1', '13C2', '13C3']
        isomasses = isomasses.loc[L, :]

    isoPeaks_mass = pd.DataFrame(columns=isomasses.index)

    massmatch['err.abs'] = np.abs(massmatch['err.ppm'])
    massmatch = massmatch.sort_values(by=['formula', 'err.abs'])
    massmatch = massmatch.drop_duplicates('formula', keep='first')
    massmatch = massmatch.drop('err.abs', axis=1)
    massmatch = massmatch.sort_values(['meas.m/z'])

    peaks = data['meas.m/z'].values
    matched_mass = np.unique(massmatch['meas.m/z'])

    for i in range(matched_mass.shape[0]):
        mz = matched_mass[i]
        if get_cal_comps == 1:
            iso_mass_range = peaks[(peaks > mz + 1.0031) & (peaks <= mz + 1.0035)]
        if get_cal_comps == 0:
            iso_mass_range = peaks[(peaks > mz) & (peaks <= mz + 7)]

        mass_diff = (iso_mass_range - mz).reshape(iso_mass_range.shape[0], 1)
        isomass = isomasses['D-value'].values.reshape(1, isomasses.shape[0])
        isomass = mass_diff - isomass
        err_range = 2 * mz * err_ppm * 10 ** -6
        loc = np.where(abs(isomass) <= err_range)
        isoPeaks_mass.loc[mz, isomasses.index[loc[1]]] = iso_mass_range[loc[0]].tolist()

    temp = pd.DataFrame(columns=['meas.m/z', 'theor.m/z', 'formula'] + 
isoPeaks_mass.columns.tolist())
    temp[['meas.m/z', 'theor.m/z', 'formula']] = massmatch[['meas.m/z', 'theor.m/z', 
'formula']].copy()
    temp.index = temp['formula'].copy()

def homo_cal(formula_data, atommasses, paras, mode=2):
    eles = ['C', 'sumH', 'N', 'O', 'P', 'S', 'elec', 'Na']

    if 'sumH' not in formula_data.columns:
        formula_data['sumH'] = formula_data['H']

    L = []
    for i in formula_data.columns:
        if i in eles:
            L.append(i)
```

```
    if 'Na' in L:
        formula_data['Na'] = 0

    formula_data['HomoMass'] = np.sum(formula_data[L].values * atommasses.loc[L, 'mass'].values.reshape(1, len(L)), axis=1)
    formula_data['NomMass'] = formula_data['HomoMass'].apply(lambda x: round(x, 0))

    if 'Homo_anal' not in formula_data.columns:
        formula_data['Homo_anal'] = 1

    temp = formula_data.loc[:, ['meas.m/z', 'formula', 'HomoMass', 'NomMass', 'Homo_anal']]
    homo_series = np.setdiff1d(['CH2', 'C2H2O', 'C2H4O', 'CO', 'CO2', 'H2O', 'NH', 'NO2'], formula_data.columns)

    for i in homo_series:
        if i not in formula_data.columns:
            E_M = paras.loc['M_' + i, 'value']
            Z_M = paras.loc['Z_M_' + i, 'value']
            temp['KMD/Z'] = (temp['NomMass'] - temp['HomoMass'] * Z_M / E_M) / (temp['NomMass'] % Z_M - Z_M)
            temp.index = temp['KMD/Z'].values

            if mode == 1:
                baseMass = temp[temp['meas.m/z'] <= 500]
            if mode == 2:
                baseMass = temp[temp['Homo_anal'] == 1]
            if mode == 3:
                baseMass = temp[(temp['meas.m/z'] <= 500) | (temp['Homo_anal'] == 1)]

            baseMass = pd.DataFrame(np.unique(baseMass['KMD/Z']), columns=['KMD/Z'])
            baseMass[i] = baseMass['KMD/Z']
            temp = pd.merge(temp, baseMass, on='KMD/Z', how='left')
            temp = temp.drop('KMD/Z', axis=1)

    temp = temp.loc[:, homo_series]
    temp = temp.dropna(how='all', axis=0)
    formula_data = formula_data.loc[temp.index, :]
    formula_data = formula_data.join(temp)

    return formula_data
```

Our full Python code for molecular formula assignments is available upon request.

The van Krevelen diagram was applied to classify the assigned formulae to different types,[24] with compositional spaces clearly delimited as:[11,25]

1. lipid-like region (O/C = 0–0.3, H/C = 1.5–2.2),
2. protein/amino sugar-like region (O/ C = 0.3–0.67, H/C = 1.5–2.2),
3. carbohydrate-like region (O/C = 0.67–1.2, H/ C = 1.5–2.2),
4. unsaturated hydrocarbon-like region (O/C = 0–0.1, H/ C = 0.7–1.5),
5. lignin-like region (O/C = 0.1–0.67, H/C = 0.7–1.5) region,

6. tannin-like region (O/ C = 0.67–1.0, H/C = 0.7–1.5), and

7. condensed aromatic region (O/ C=0–0.67, H/C = 0.33–0.70).

The double-bond equivalents (DBE) and modified aromaticity index (AI_{mod}) were calculated to reflect the unsaturated degree and aromaticity of molecules following:[26,27]

$$\mathrm{DBE} = 1 + 0.5\times(2n_{\mathrm{C}} - n_{\mathrm{H}} + n_{\mathrm{N}} + n_{\mathrm{P}}) \tag{S24}$$

$$\mathrm{AI}_{\mathrm{mod}} = \frac{1 + n_{\mathrm{C}} - 0.5\times n_{\mathrm{O}} - n_{\mathrm{S}} - 0.5\times(n_{\mathrm{N}} + n_{\mathrm{P}} + n_{\mathrm{H}})}{n_{\mathrm{C}} - 0.5\times n_{\mathrm{O}} - n_{\mathrm{N}} - n_{\mathrm{S}} - n_{\mathrm{P}}} \tag{S25}$$

The intensity-weighted molecular parameters (M_w), *i.e.*, intensity-weighted molecular weight (MW_w), intensity-weighted double-bond equivalents (DBE_w), intensity-weighted elemental ratios (H/C_w, O/C_w, and N/C_w), intensity-weighted modified aromaticity index ($AI_{mod,w}$), were calculated following equation:[28]

$$\mathrm{M}_{\mathrm{w}} = \left(\sum_i I_i \times \mathrm{M}_i\right) / \sum_i I_i \tag{S26}$$

where I_i and M_i are the relative abundance and molecular parameter value of peak i, respectively. The relative abundance is calculated as the abundance of the individual peak divided by the maximum abundance in a given FTICR-MS spectrum.

The $\log_{10} C_0$ values of molecules were calculated to reflect volatility following:[1]

$$\log_{10} C_0 = (n_{\mathrm{C}}^0 - n_{\mathrm{C}})\times b_{\mathrm{C}} - n_{\mathrm{O}}\times b_{\mathrm{O}} - 2\times\frac{n_{\mathrm{C}}\times n_{\mathrm{O}}}{n_{\mathrm{C}} + n_{\mathrm{O}}}\times b_{\mathrm{CO}} - n_{\mathrm{N}}\times b_{\mathrm{N}} - n_{\mathrm{S}}\times b_{\mathrm{S}} \tag{S27}$$

where, n_C, n_H, n_O, n_N, n_P, and n_S are the number of carbon, hydrogen, oxygen, nitrogen, and sulfur atoms, respectively. n_C^0 and b_{CO} are the reference carbon number and carbon-oxygen nonideality, respectively. The b_C, b_O, b_N, and b_S are the contributions of each atom to $\log_{10}C_0$ and their values are provided in Table S14.

Note S2. Results and Discussion

Note S2.1 Thermodynamic Characteristics of Food Waste Digestate Pyrolysis

TG/DTG curves of FWD pyrolysis exhibited a notable shift toward higher temperatures with increasing pyrolysis rates (Figure S2a), which is a common trend observed in FWD pyrolysis.[4,29] This shift is attributed to the thermal resistance of FWD, which delays thermal equilibrium because of substantial instantaneous thermal energy. The DTG curves outlined three distinct pyrolysis stages. Initially, evaporation of inherent moisture and light volatiles occurred (< 200 ℃),[30] followed by degradation of hemicellulose, cellulose, lignin, and extractives (200−550 ℃),[4] and ash (i.e. $CaCO_3$) fusion (> 550 ℃).[31] Significant pyrolysis occurred in 200−550 ℃. Analyzing the DTG peak positions at 10 ℃·min^{-1}, it can be fitted by 4 distinct peaks representing decompositions of different components based on previous studies.[4,29,30,32,33] The DTG shoulder around 285 ℃ primarily resulted from the decomposition of carbohydrates and hemicellulose.[32,33] The 325 ℃ DTG peak was attribute to the decomposition of proteins, while the peak at 350 ℃ corresponded to the decomposition of cellulose and lipids.[32,33] Within the 350−550 ℃ range, the even DTG trough may be attributed to decomposition of lignin or the secondary decomposition of other components.[30,34–36]

Gaussian deconvolutions of DTG curves were fitted following previous studies to analyze the contributions of different components (Figures 1a, 1b and S1).[3] Most component peak temperatures increased with higher heating rates, attributed to uneven heating and heat transfer limitations.[37] Higher heating rates significantly reduced the contribution percentage of hemicellulose which had lower temperature stability. This contrasted with the decomposition of sugar cane residue, where components with lower thermal stability showed a larger decomposition contribution percentage at higher heating rates.[3] These findings indicated that the pyrolysis process of hemicellulose was influenced by the coexisting matrix of different biomasses. Additionally, components had maximum weight loss rates observed at 50 °C·min^{-1}, similar to a previous study,[3] suggesting that under high heating conditions, enhanced heat flow slightly strengthened the processes generating volatile compounds during feedstock pyrolysis.

Note S2.2 Identification of Gaseous Products via TG-FTIR

The pyrolytic gases were analyzed using TG-FTIR at a heating rate of 10 ℃· min^{-1}. Figure S3a showed the three-dimensional FTIR spectra of gaseous products evolved at different pyrolysis temperatures. The FTIR spectra of gases were extracted at DTG peak temperatures to identify their compositions (Figure S3b, Table S4). Nitrogen-containing gases included NH_3 (N-H bending, 966 cm^{-1}), HCN (C-H bending, 719 cm^{-1}), and HNCO (C=N stretching, 2282 cm^{-1}), while NO (N-O stretching, 1900 cm^{-1}) was too weak to be clearly counted. Oxygen-rich gases included ethers (C-O/C-C stretching, 1159 cm^{-1}), carboxylic acids/aldehydes (C=O stretching, 1749 cm^{-1}), CO_2 (C=O stretching, 2336 cm^{-1}), CO (C-O stretching, 2181 cm^{-1}) and H_2O/phenols (O-H stretching, 3736 cm^{-1}).[38] The gaseous products without nitrogen and oxygen mainly contained CH_4 (C-H stretching, 2968 cm^{-1}) and aromatic structures (C=C stretching, 1508 cm^{-1}).

Analogous to the TG/DTG results, the gas emissions were categorized into 3 stages. At < 200 ℃, peaks corresponding to H_2O/phenols, C=O, and C=C occurred at 110 ℃, aligning with the DTG peak. It indicated the releases of light compounds like phenols, ketones, aldehydes, and aromatics. The increasing N-H peak until 195 ℃ suggested the decomposition of ammonium salts.[39] In 200−550 ℃, substantial emissions of gases correlated with the intensified decomposition of components as indicated in the DTG curve. CO_2 emission was predominant, highlighting decarboxylation as major contributors. Aliphatic compound emissions were significantly lower than those for other gaseous products, contrasting to other studies,[4,29] probably due to the low lipid content in FWD. At > 550 ℃, the emergence of C=C peaks at 559, 676, and 753 ℃ indicated benzene ring cracking during charring. The elevated release of CO and C=C at 650 ℃ likely resulted from the decarbonylation reaction or the Boudouard reaction involving CO_2 and char.[29] Gas concentrations at different temperatures were calculated using the integral values of their FTIR spectra (Figure S3c–d).[40]

Note S2.3 Identification of Functional Groups in Biochar, DBCs and BCAs

The evolution of functional groups in biochar, DBCs and BCAs as a function of charring temperature was illustrated in Figure 2c–e. The main wavenumber features in the FTIR spectra of biochar, DBCs and BCAs were similar. Key FTIR peaks in 1150–850 cm^{-1} were attributed to C-O stretching in ethers and alcohols in polysaccharides or C-N stretching.[41] These peaks were usually observed in the digestate-derived biochar.[42,43] For DBCs, obvious FTIR absorbances appeared in 1250–1150 cm^{-1}, more pronounced than those in biochar and BCAs. This can be attributed to the enrichments of the compounds containing ester/amine groups. It indicated that ester/amine groups (1250–1150 cm^{-1}) in biochar were more mobile during leaching compared to ether/alcohol groups (1150–950 cm^{-1}) and aromatic structures (950–850 cm^{-1}). This enrichment of the mobile ester/amine groups may result in unobvious negative signals in their hetero-asynchronous 2D-FTIR-COS map (Figure 2f).

The peak above 3600 cm^{-1} corresponds to the vibration of O-H groups in mineral matter, still detectable in biochar and BCAs obtained at temperatures above 550 ℃.[43] These O-H group vibrations above 3600 cm^{-1} were only identified in biochar and BCAs, not in DBCs, suggesting their stability during leaching. The intense peak near 3400 cm^{-1} is attributed to O-H and N-H groups from organic matter such as phenols and heterocyclic nitrogen compounds.[44] Aromatic and heteroaromatic compounds are also confirmed by C-H vibrations near 850 cm^{-1}. Bands at 2941 and 2857 cm^{-1} correspond to aliphatic CH_x asymmetric and symmetric stretching vibrations, respectively. The existence of aromatic C=C and CH_x is confirmed by the C=C and CH_2 scissoring vibrations at 1600 and 1438 cm^{-1}, respectively. Bands around 1650 cm^{-1} are due to C=O stretching in amides.

A detailed list of the assignments of functional groups in biochar, DBCs, and BCAs is presented in Table S9. Overall, the presences of O-H and N-H groups (3400 cm^{-1}), aromatic and heterocyclic structures (3400, 1600, 850 cm^{-1}), aliphatic CH_x groups (2941, 2857 and 1438 cm^{-1}), amide group (1650 cm^{-1}) and alcoholic/ether/ester groups (1300–1000 cm^{-1}) in the FTIR spectra of biochar, BCAs, and DBCs indicated synergetic changes in functional groups among them.

Note S2.4 Precursors Susceptible to Possible Primary Gas-releasing Reactions

Under –ESI, the "precursors" involved in the possible primary releases of NH_3, HNCO, HCN, and H_2O featured higher $AI_{mod,w}$ values compared to those associated with other gases (Table S19), indicating higher aromaticity. Figures S17 to S18 indicated that these primary gas releases were mainly linked to formulae in the lignin-like region, suggesting they might be heterocyclic nitrogen compounds, aromatic proteins and aromatic compounds with acidic groups.[45] In addition to lignin-like precursors, the releases of CO_2 and CO were related to more decompositions of precursors with lower $AI_{mod,w}$ and higher H/C_w values (i.e., protein-like compounds), while precursors involved in dealkylation and dehydrogenation reactions ($-CH_2$, $-CH_4$, and $-H_2$) contained more lignin-like molecules with relatively high O/C_w (Table S19).

Under +ESI, the "precursors" involved in the releases of all gases featured lower $AI_{mod,w}$ but higher MW_w than those identified under –ESI (Figures S17–S18, and Tables S19–S20), indicating a higher inclusion of basic functional groups in labile macromolecules such as polypeptides. Specifically, the precursors involved in the release of CO_2 across all DBCs under +ESI had the lowest $AI_{mod,w}$ but the highest N/C_w (except for DBC550) and O/C_w, suggesting that this gas release involved more decomposition of labile protein-like compounds compared to other gas-releasing reactions.

Note S2.5 Gas-Releasing Reaction Directions in van Krevelen Diagrams

The reaction processing vectors from the initial "precursor" to the final "product" in the van Krevelen diagram can be marked to indicate and predict the reaction directions. For a precursor of "$C_cH_hN_nO_oS_s$", its coordinate in the van Krevelen diagram is expressed as:

$$(x_i, y_i) = \left(\frac{O}{C}, \frac{H}{C}\right) \tag{S28}$$

The coordinate of its "product" (x_f, y_f) based on the loss of specific numbers of *C*, *H*, *N*, and *O* atoms in the primary gas-releasing reactions can be found in Table S20. For instance, in the "–HCN" process, the coordinate of the product can be described by:

$$(x_f, y_f) = \left(\frac{O}{C-1}, \frac{H-1}{C-1}\right) \tag{S29}$$

The differences in the coordinate values between the "precursor" and the "product", denoted as Δx and Δy, determine the reaction vector direction:

$$\Delta x = x_f - x_i \tag{S30}$$

$$\Delta y = y_f - y_i \tag{S31}$$

If $\Delta x > 0$, it means that the gas-releasing process shifts the "product" to a higher *O/C* space than its "precursor". Similarly, if $\Delta y > 0$, the process shifts the "product" to a higher *H/C* space compared to the "precursor".

Taking the "–HCN" process as an example, the Δx and Δy between the "precursor-product pairs" can be expressed as:

$$\Delta x = x_f - x_i = \frac{O}{C-1} - \frac{O}{C} \tag{S32}$$

$$\Delta y = y_f - y_i = \frac{H-1}{C-1} - \frac{H}{C} \tag{S33}$$

Since the *C* number > 1, Δx is always positive, indicating an elevated *O/C* by the "–HCN" process. For $\Delta y > 0$, *if H/C* >1, $\Delta y > 0$, and *if H/C* < 1, $\Delta y < 0$. This means the "–HCN" process shifts the "product" upward for precursors with *H/C* > 1 to a higher H/C space and downward for those with *H/C* < 1.

The slope of coordinate between the "precursor" and the "product", namely $\Delta y/\Delta x$, shows the angle of reaction vectors deviating from the *O/C* coordinate axis in the van Krevelen diagram:

$$\frac{\Delta y}{\Delta x} = \frac{y_f - y_i}{x_f - x_i} \tag{S34}$$

For example, the slope for –CO reaction vector can be expressed as:

$$\frac{\Delta y}{\Delta x} = \frac{H/C}{O/C-1} \tag{S35}$$

while for the –CO_2 process, it is given by:

$$\frac{\Delta y}{\Delta x} = \frac{H/C}{O/C-2} \tag{S36}$$

The slope values depend on the precursor' coordinates (*O/C*, *H/C*). For the same precursor located in the space with *O/C* < 1, the negative slope for the "–CO" processing vector is lower than that of "–CO_2" vector, indicating the processing vector for the "–CO" process deviates

from the *x*-axis more than that for the "$-CO_2$" process.

Based on detailed analysis of reaction vector directions (Δx, Δy) and slops ($\Delta y/\Delta x$), the reaction directions from the "precursor" to the "product" can be marked in the van Krevelen diagrams (Figure S19). Beyond these primary defunctionalization processes, the secondary and even multi defunctionalization processes for DBC molecule production can be traced via iterating the processing vectors multiple times.[23,46]

Note S2.6 Changes of Aromaticity via Gas-Releasing Reactions

Changes in aromaticity were accessed by comparing the modified aromaticity index (AI_{mod}) [26,27] of the initial "precursor" and the final "product" in various possible gas-releasing reactions. The initial AI_{mod} of the precursor ($AI_{mod,i}$) and the final AI_{mod} of the product ($AI_{mid,f}$) were compared. For a precursor with the formula "$C_cH_hN_nO_oS_sP_p$", $AI_{mod,i}$ was given by "x/y":

$$AI_{mod,i} = \frac{x}{y} = \frac{1 + n_C - 0.5\times n_O - n_S - 0.5\times(n_N + n_P + n_H)}{n_C - 0.5\times n_O - n_N - n_S - n_P} \quad (S37)$$

The $AI_{mod,f}$ for products of different gas-releasing reactions could be expressed as a function of x and y, f(x, y).Conditions for an elevated $AI_{mod,f}$ compared to $AI_{mod,i}$ were derived from the inequation: f(x, y) > x/y (Table S22).

For example, in the "–HCN" process, $AI_{mod,f}$ of the "product" was expressed as:

$$AI_{mod,f} = \mathrm{f}\left(x, y\right) = \frac{x - 1 + 0.5\times(1+1)}{y - 1 + 1} = x/y \quad (S38)$$

$AI_{mod,f}$ remains equal to $AI_{mod,i}$, indicating unchanged aromaticity. However, in more complex reactions, such as the "–HCNO" process, if $AI_{mod,i} > 1$, then $AI_{mod,f}$ increases, and if $AI_{mod,i} < 1$, then $AI_{mod,f}$ decreases (Table S22). Since all precursors had the $AI_{mod,i} < 1$ (Figures 4e–f and S21a–b), the aromaticity is reduced via the "–HCNO" process. Additionally, all precursors had O/C < 1 (Figure S21c–f). By analyzing other gas-releasing reactions, conditions for the elevated AI_{mod} are summarized in Table S22. Dehydration, dealkylation, deamination, and dehydrogenation ($-H_2O$, $-CH_2$, $-CH_4$, $-NH_3$ and $-H_2$) elevate the AI_{mod}. Decarboxylation ($-CO_2$) and dehydrocyanation (–HCN) do not change AI_{mod}, while decarbonylation (−CO) and deamidation (−HNCO) reduce AI_{mod}. The summary of changes in AI_{mod} due to possible primary gas-releasing reactions is provided in Figure S19b. Overall, $-H_2O$, $-H_2$, $-CH_2$, $-CH_4$, $-NH_3$ reactions promote aromatization, while –HNCO and –CO reactions facilitate the breakdown of aromatic molecules.

The relative number percentages of the precursor-product pairs for DBC formulae identified under ±ESI based on possible gas-releasing reactions were demonstrated in Figure S20b–d. The top four primary gas-releasing reactions for DBC350 were $-H_2O$, $-CO_2$, $-H_2$, and $-CH_4$, while for DBC450, they were $-H_2O$, $-CH_4$, $-H_2$, $-CO_2$. The dehydration ($-H_2O$) predominantly explained the origin of both DBC350 and DBC450. This can be attributed to the lower energy barriers of dehydration reactions, as shown by their priority temperature responses (Table S8). Besides dehydration, the $-CO_2$ rection was more significant for DBC350 production, while $-CH_4$ was more relevant for DBC450, aligning with the temperature-dependent priority of $-CO_2$ over $-CH_4$ (Table S8). Additionally, more significant CO_2 release than CH_4 was also observed at 350 ℃ compared to 450 ℃ (Figure 1c). This indicated that the intensive gasification reactions at specific pyrolysis temperature provided deep insights into DBC formation.

For DBC550, the top four gas-releasing reactions were $-CO_2$, −HCNO, −CO, and −HCN. The significant reduction of dehydration products in DBC550 can be attributed to their decomposition into gases or condensation into biochar.[29] Notably, the releases of HCNO and CO reduced the aromaticity (AI_{mod}), comprising 18.2%, 16.6% and 26.8% of the total gas-releasing reactions for DBC350, DBC450, and DBC550 formation, respectively. Their preferences in higher pyrolysis temperature (550 ℃) may be related to that the reduction in aromaticity was accompanied by the breakdown of C=C bonds. It required higher energy input than the aromatization processes to destroy C-O or C-C bonds. This aligned with the occurrence of peaks for HCNO and CO at higher 650 ℃ (Figure 1c). These high-energy barrier gas-releasing reactions (i.e., –CO) also exhibited a delayed temperature-dependent gas release sequence (Table S8).

Overall, primary gas-releasing reactions with lower energy barriers or priority temperature-dependent sequences mainly contributed to DBC formation at lower temperatures (350 and 450 ℃). As the temperature rose i.e., increasing to 550 ℃, initial gas-releasing related DBC products decomposed step-by-step, reducing their amounts. High-energy barrier gas-releasing reactions gradually dominated the formation of high-temperature DBC550 substances. Besides temperature-dependent sequences, the intensive gasification reactions of char or bio-oils also provided important references for DBC formation. These observations indicate that the evolution of gases offers valuable insights into DBC formation and prediction.

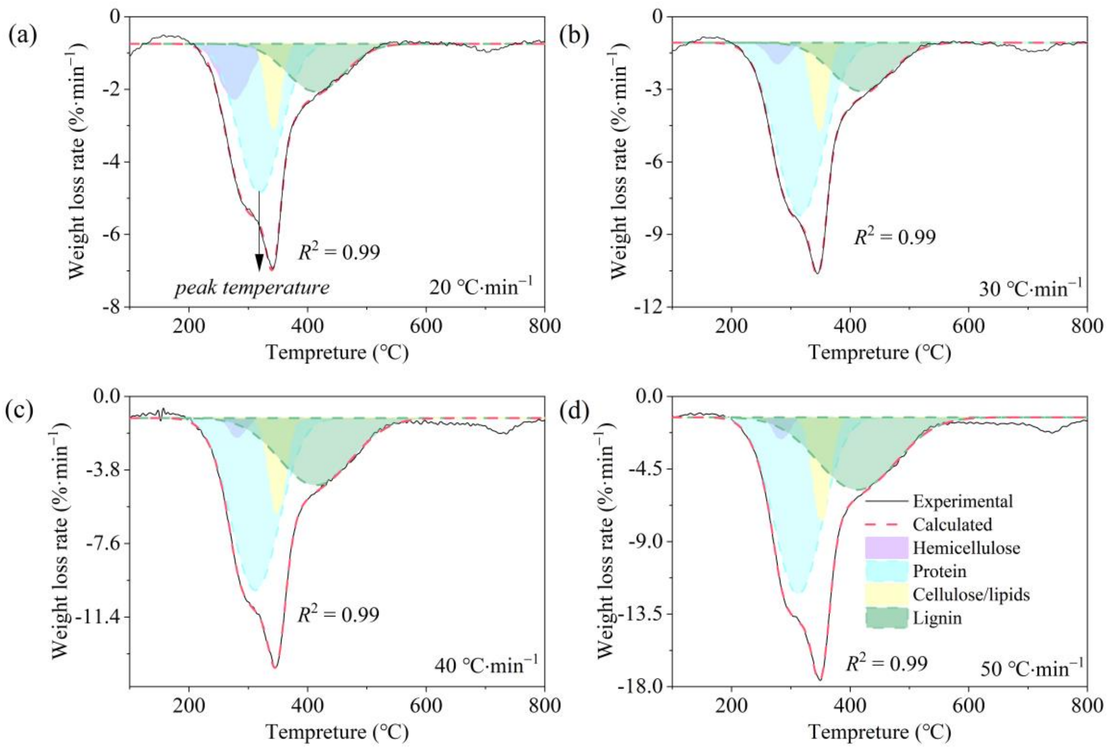

Figure S1. Optimized decomposition processes for components of FWD pyrolysis at four heating rates based on the Gaussian model.

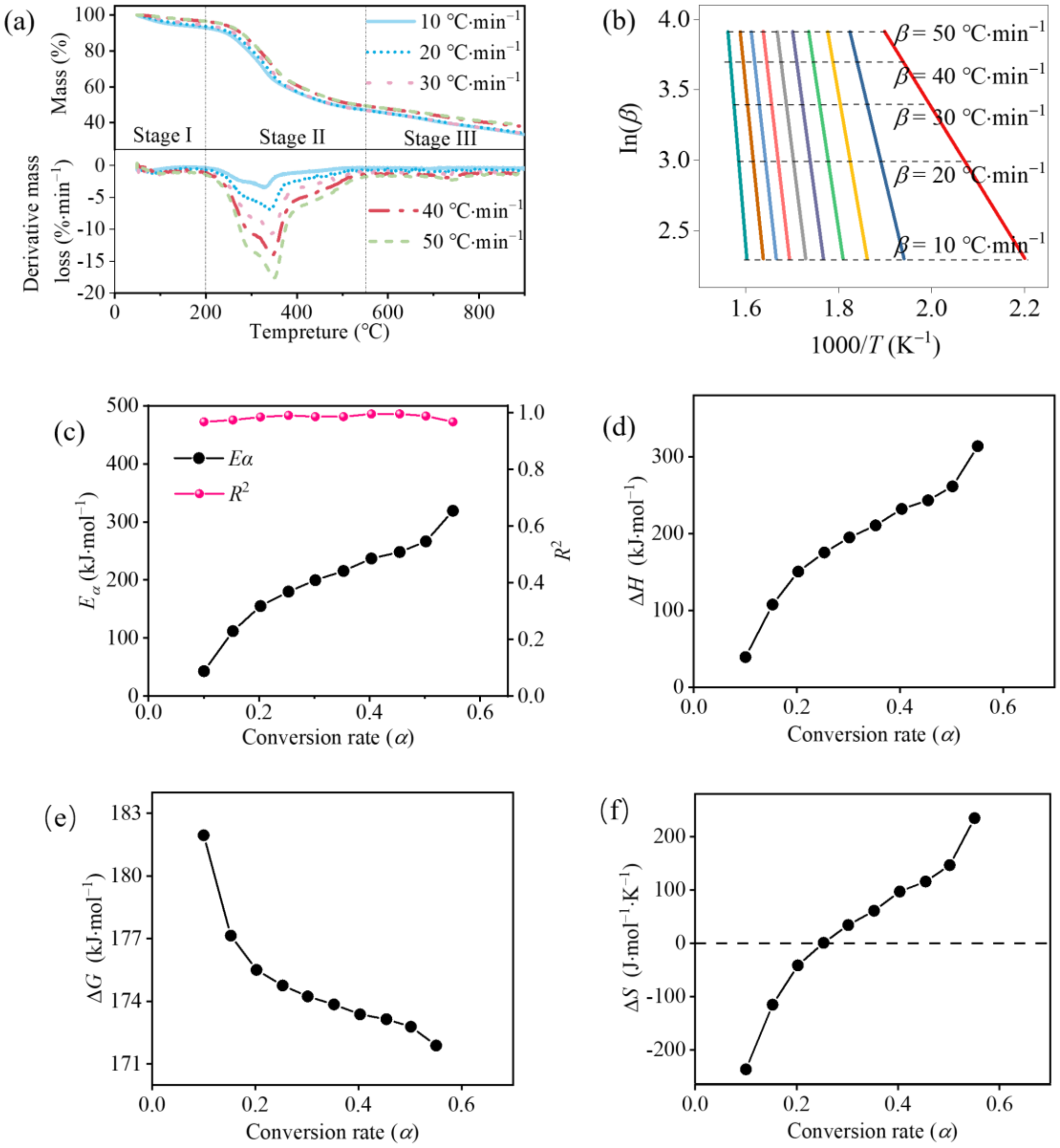

Figure S2. (a) TG and DTG curves and (b) Arrhenius plots at five heating rates of FWD pyrolysis; plots of (c) $E\alpha$ and R^2, (d) ΔH, (e) ΔG and (f) ΔS values as a function of the conversion rate.

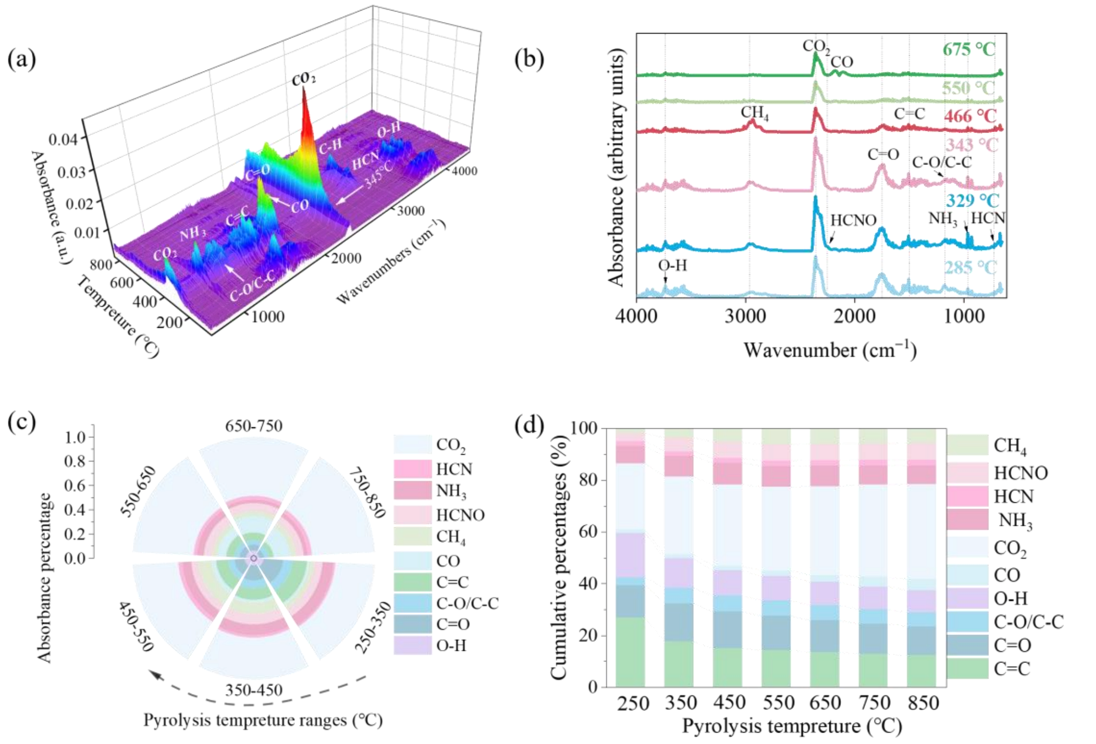

Figure S3. (a) Three-dimensional FTIR, (b) FTIR absorbances at DTG peak temperatures, (c) cumulative FTIR absorbances at different temperature ranges, and (d) cumulative FTIR absorbance at specific temperatures for gaseous products released during FWD pyrolysis.

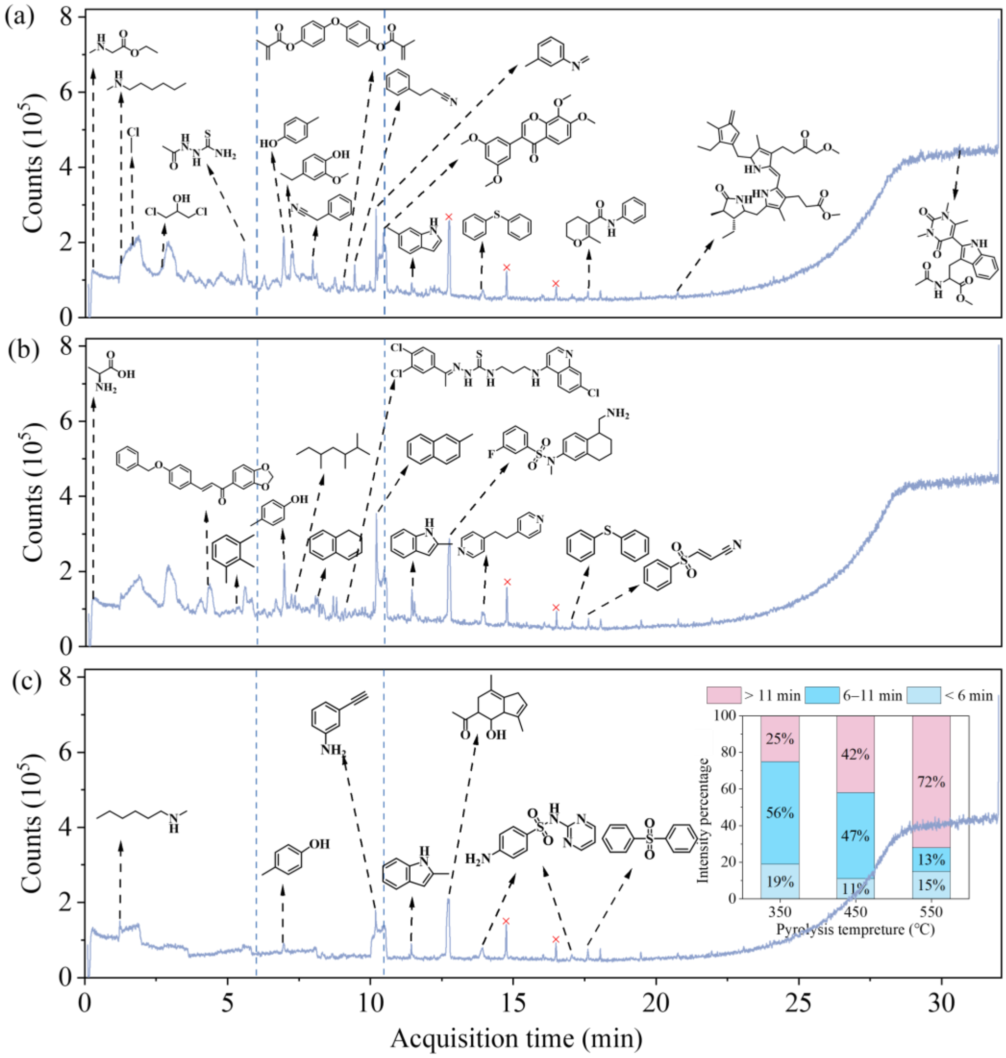

Figure S4. GC-MS total ion chromatograms at pyrolysis temperatures of (a) 350 ℃, (b) 450 ℃, and (c) 550 ℃. Red "×" marks the column bleeding. The illustration depicts the relative abundances of volatile organic products within different GC-MS acquisition time.

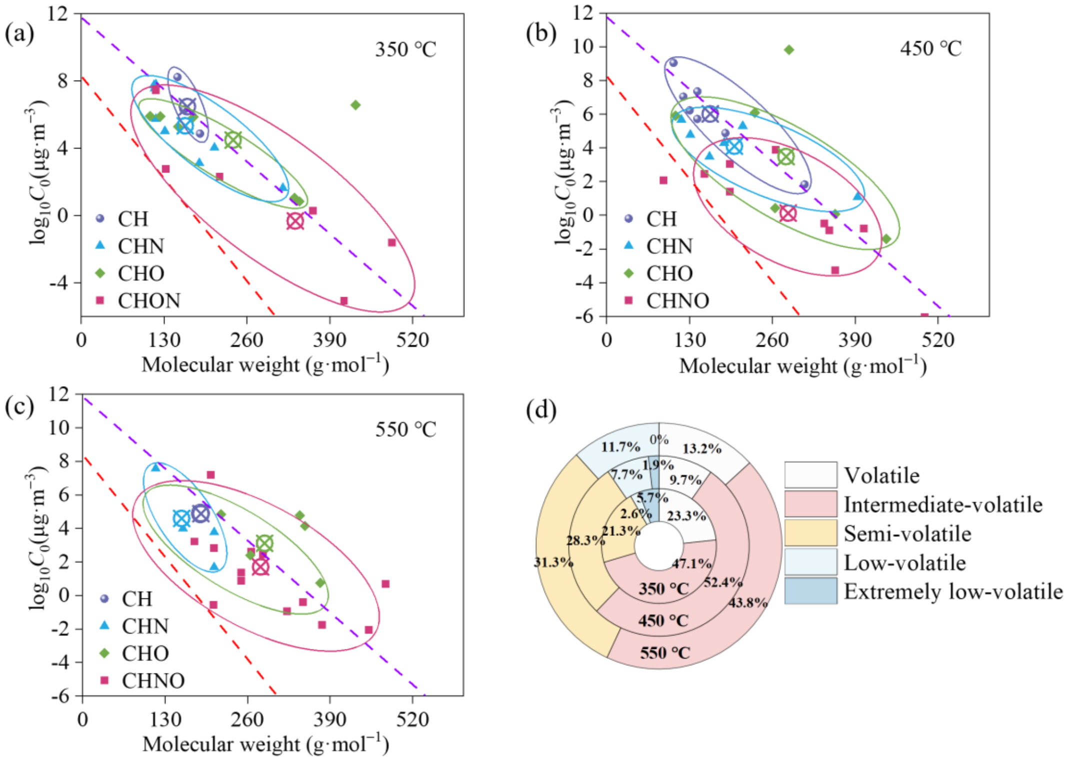

Figure S5. Volatility distribution of volatile organic compounds identified at (a) 350 ℃, (b) 450 ℃, (c) 550 ℃ from FWD pyrolysis and (d) percentages of compounds with varying volatility. Upper (purple) and lower (red) boundaries in $\text{Log}_{10}\, C_0$ vs MW plot represent linear alkanes and sugar alcohols, respectively.[1] The circular crossing markers show the mean values.

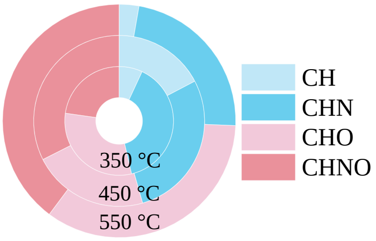

Figure S6. Relative TG-GC-MS intensities of pyrolytic volatile organic “CH”, “CHN”, “CHO” and “CHNO” compounds released at 350 ℃ (Table S5), 450 ℃ (Table S6) and 550 ℃ (Table S7). The classification of volatile organic compounds was based on the presence of nitrogen and oxygen atoms.

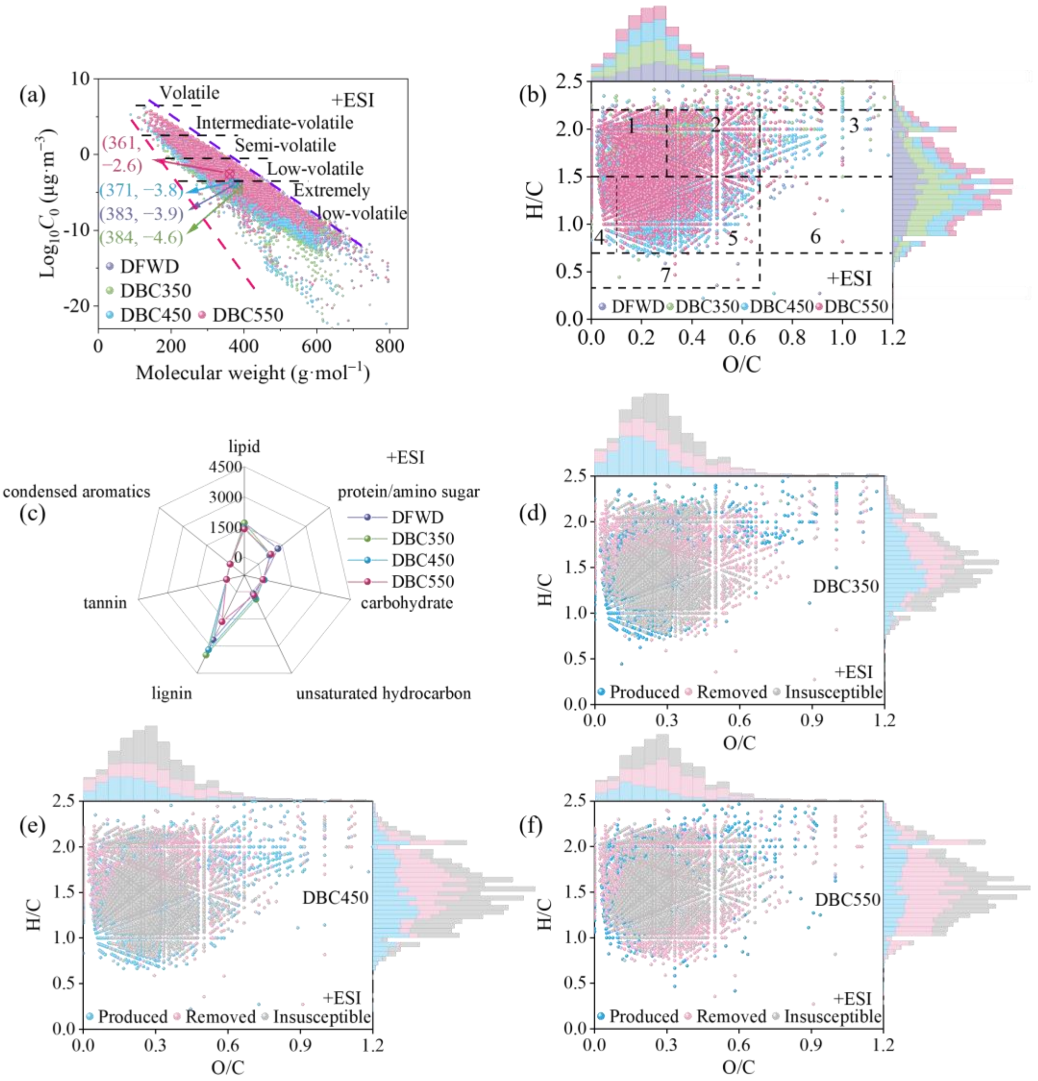

Figure S7. (a) Molecular corridors of Log$_{10}C_0$ vs. molecular weight for DBC molecules identified under +ESI. Circular crossing markers represent the average values of corridors. Dotted lines represent alkanes C_nH_{2n+2} (upper purple line) and sugar alcohols $C_nH_{2n+2}O_n$ (lower pink line); (b) van Krevelen diagrams of DFWD and DBC molecules identified under +ESI and (c) the numbers of molecules in different compound classes; (d)–(f) van Krevelen diagrams of "produced", "removed" and "insusceptible" formulae identified under +ESI. Numbers in different regions of the van Krevelen diagram represent different types of molecules as demonstrated in Note S1.7.

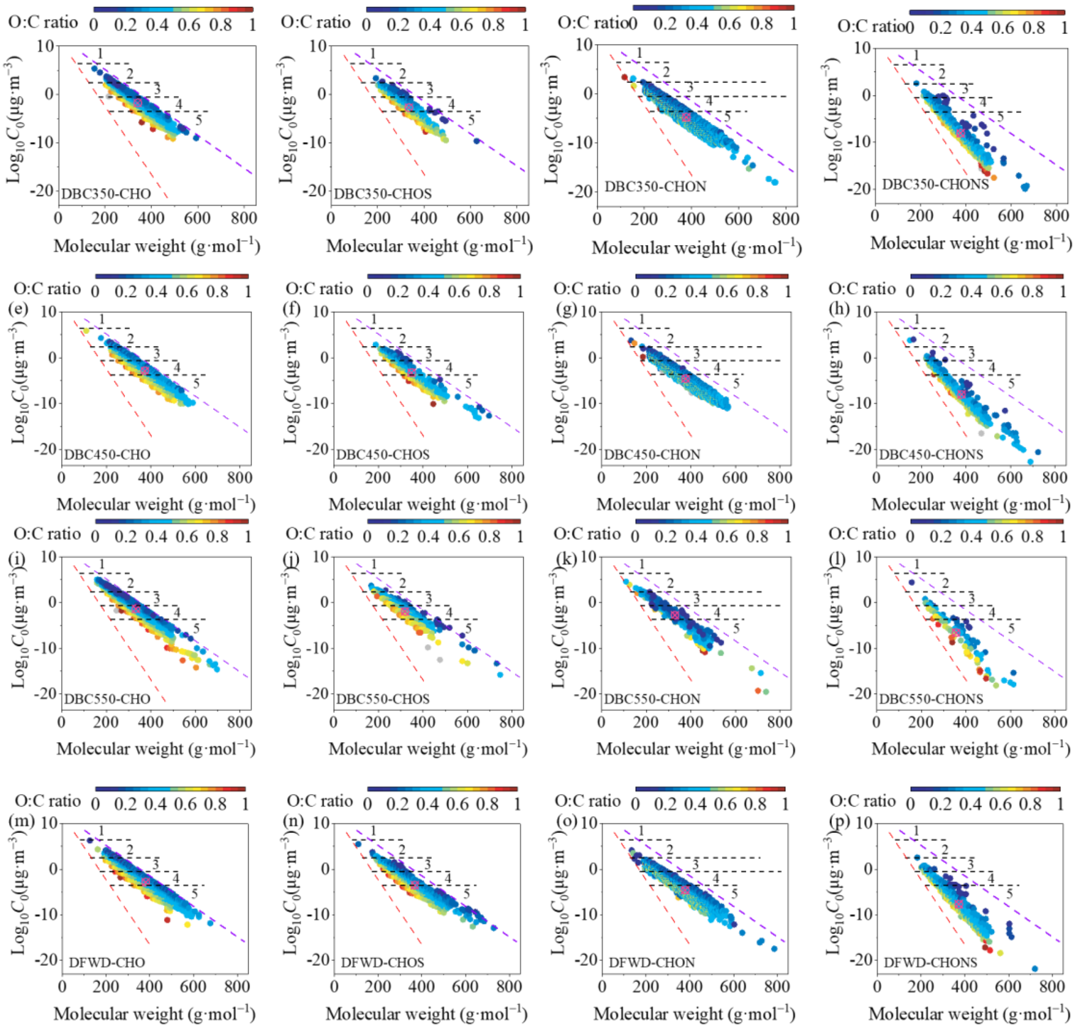

Figure S8. Molecular corridors of Log$_{10}C_0$ vs. molecular weight for DBC formulae identified under −ESI and the signals represent the O/C values for each formula. Circular crossing markers represent the average values of corridors. Dotted lines represent representing alkanes C_nH_{2n+2} (upper purple line) and sugar alcohols $C_nH_{2n+2}O_n$ (lower pink line).[1] Numbers in different regions represent volatility (Note S1.4).

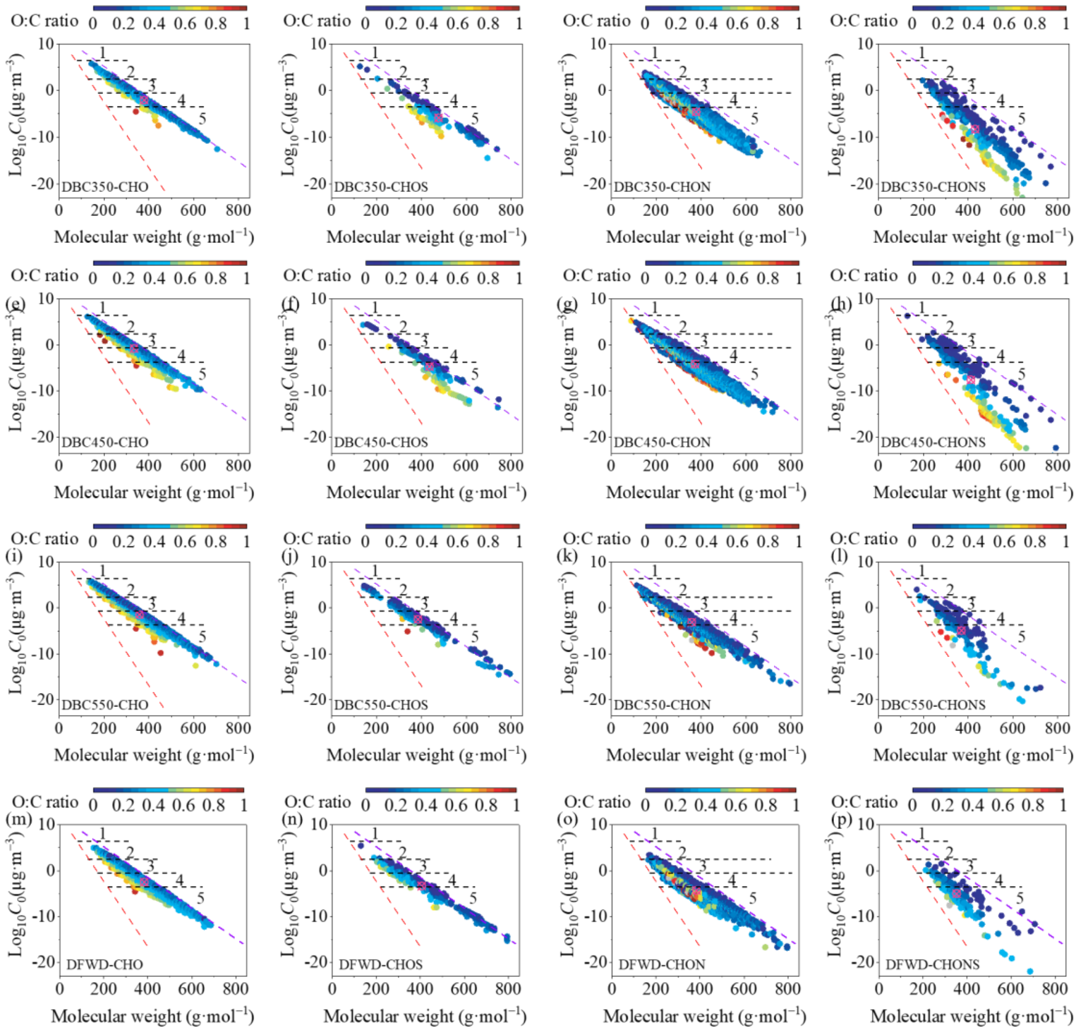

Figure S9. Molecular corridors of Log$_{10}C_0$ vs. molecular weight for DBC formulae identified under +ESI and the signals represent the O/C values for each formula. Circular crossing markers represent the average values of corridors. Dotted lines represent representing alkanes C_nH_{2n+2} (upper purple line) and sugar alcohols $C_nH_{2n+2}O_n$ (lower pink line). Numbers in different regions represent volatility (Note S1.4).

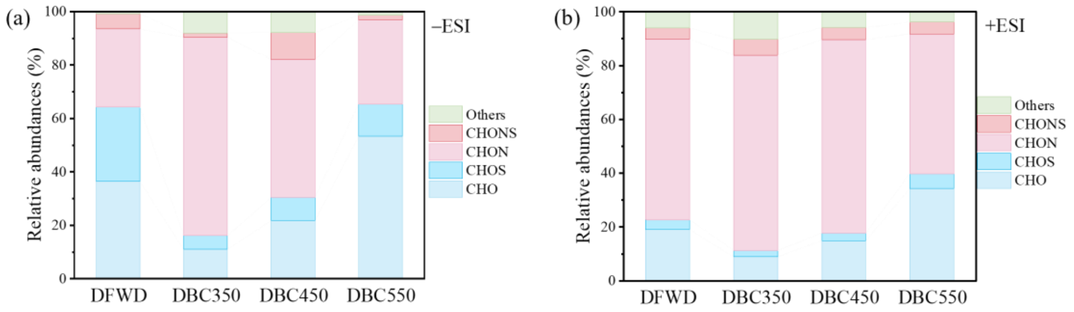

Figure S10. Relative FTICR-MS spectral abundances of CHO, CHOS, CHON, CHONS, and "Others" (mainly P-containing compounds) for DFWD and DBC samples.

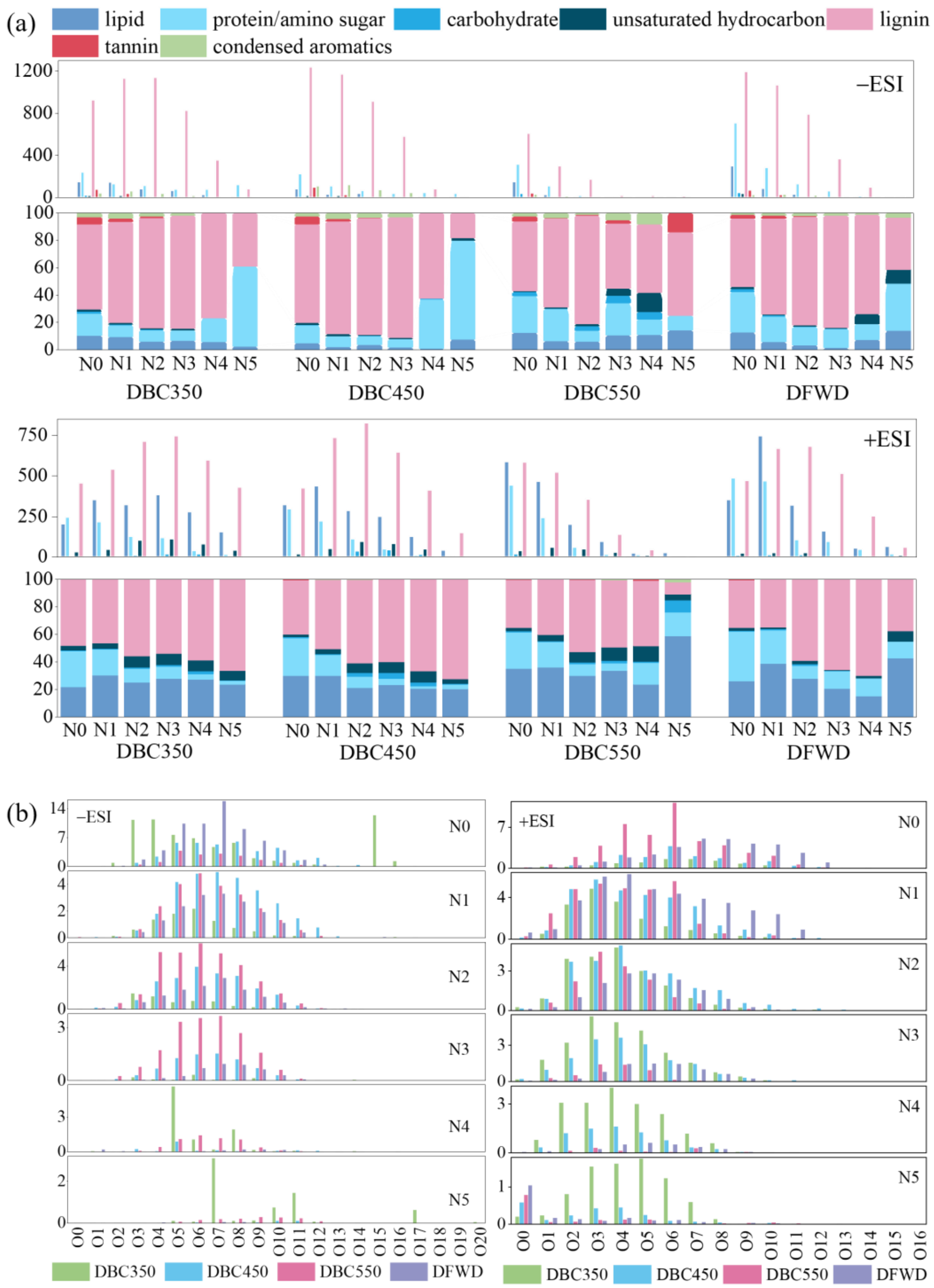

Figure S11. (a) Numbers and relative percentages of different types of molecules classified by van Krevelen diagrams, and (b) the relative spectral abundances of N- and O-formulae identified in DFWD, DBC350, DBC450 and DBC550.

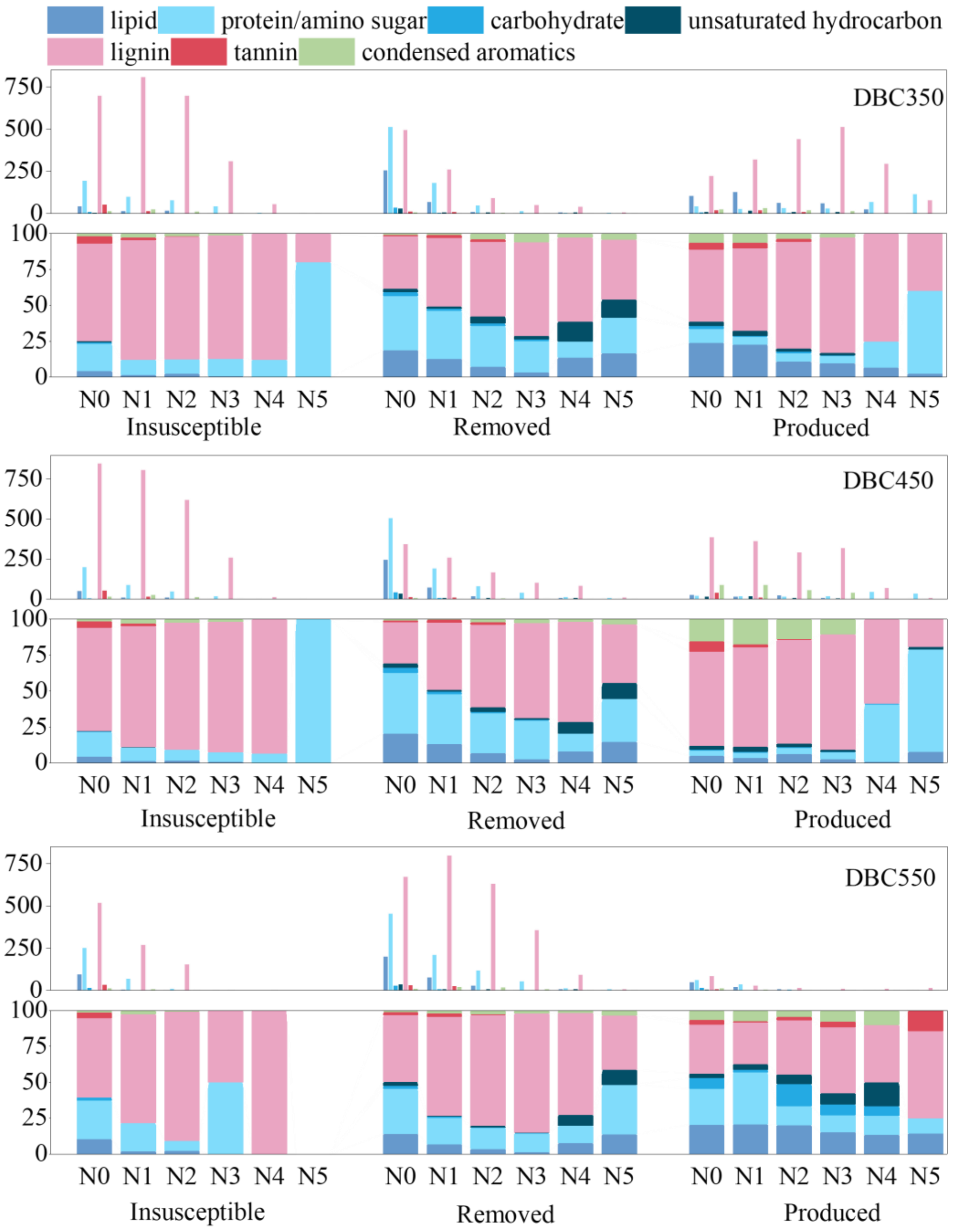

Figure S12. Numbers and percentages of different types of molecules classified via van Krevelen diagram for the "produced", "removed" and "insusceptible" molecules categorized under –ESI.

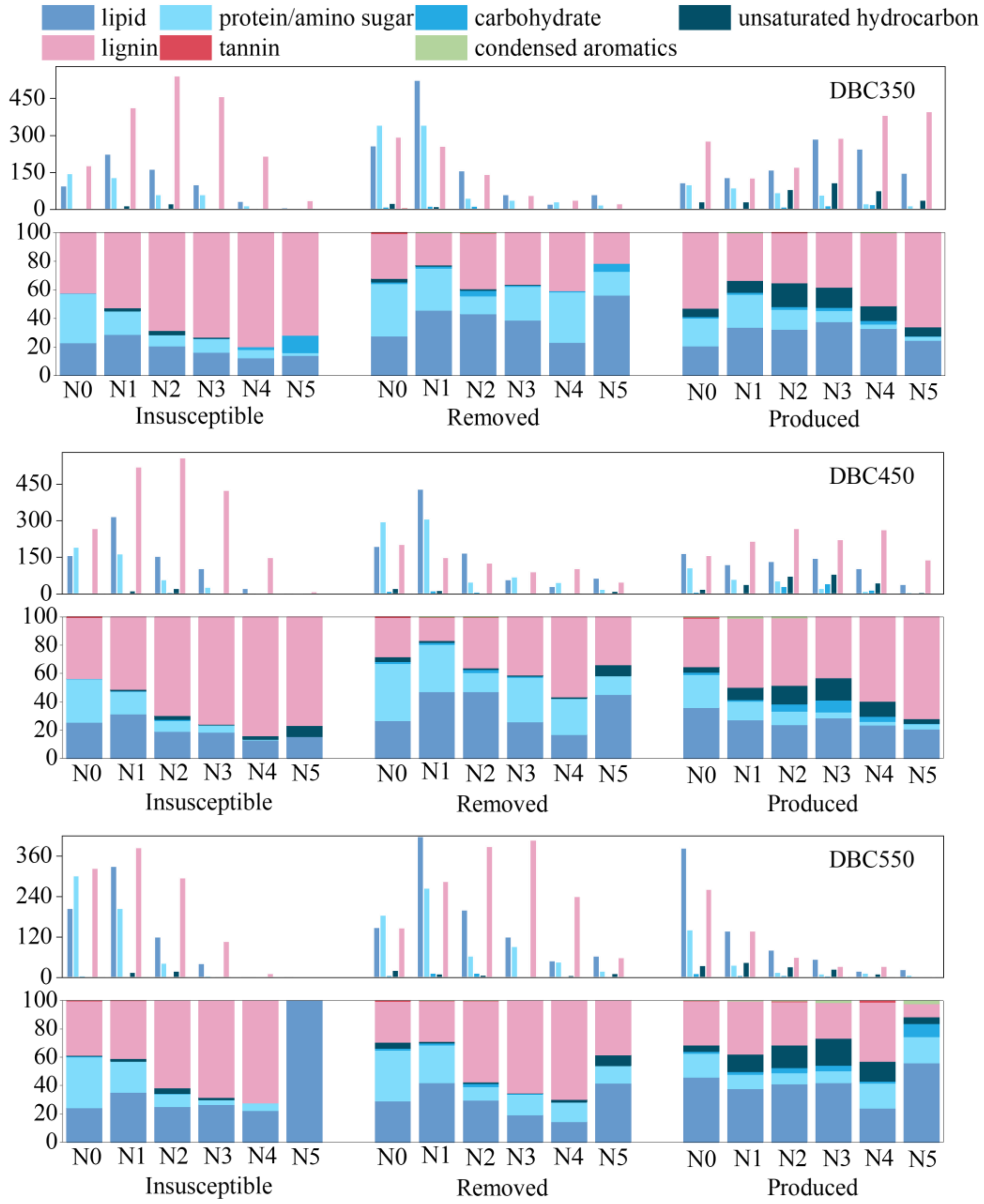

Figure 13. Numbers and percentages of different types of molecules classified via van Krevelen diagram for the “produced”, “removed” and “insusceptible” molecules categorized under +ESI.

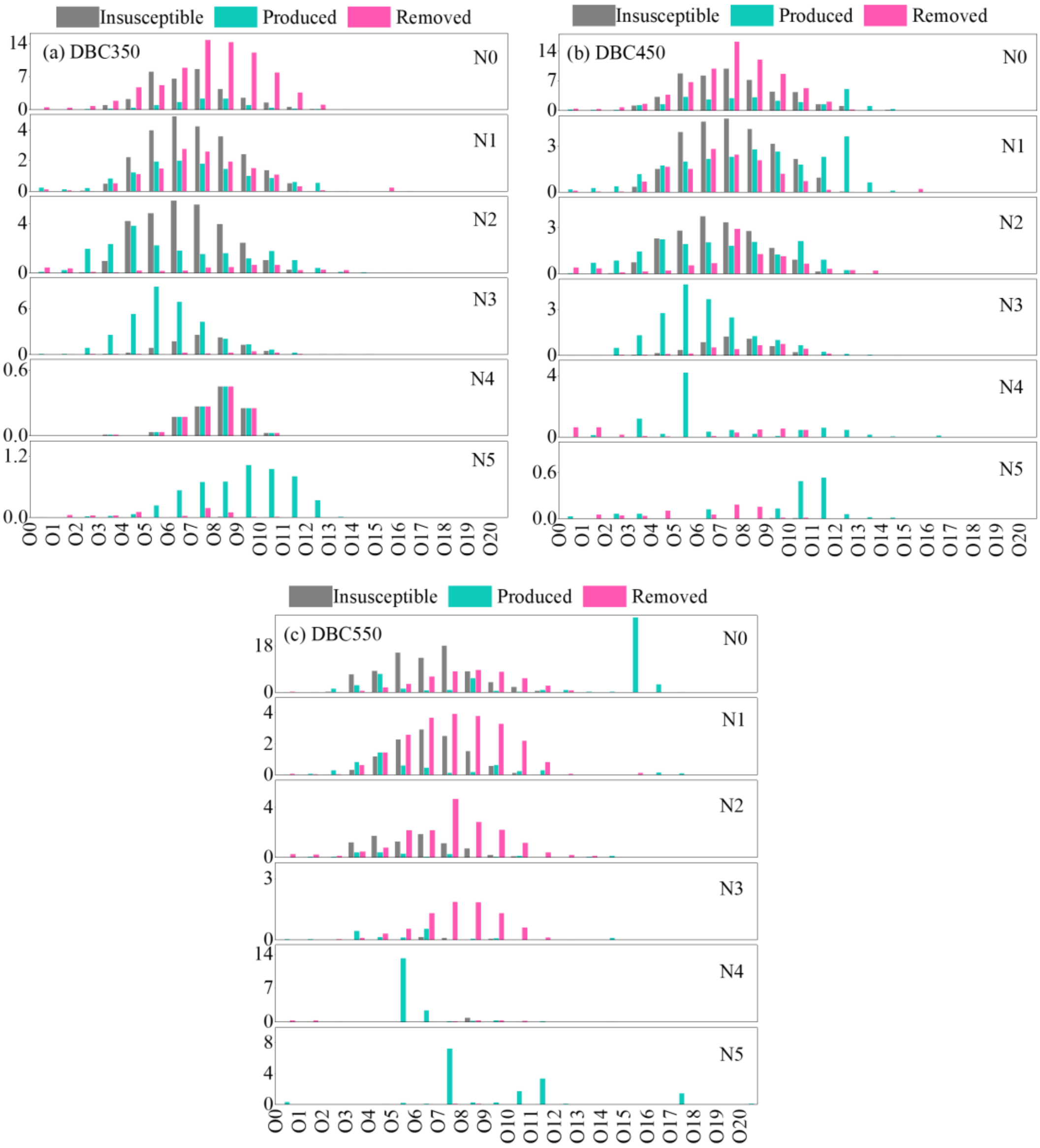

Figure S14. Changes of the relative spectral abundances of N- and O-formulae identified under −ESI during production of (a) DBC350, (b) DBC450, and (d) DBC550.

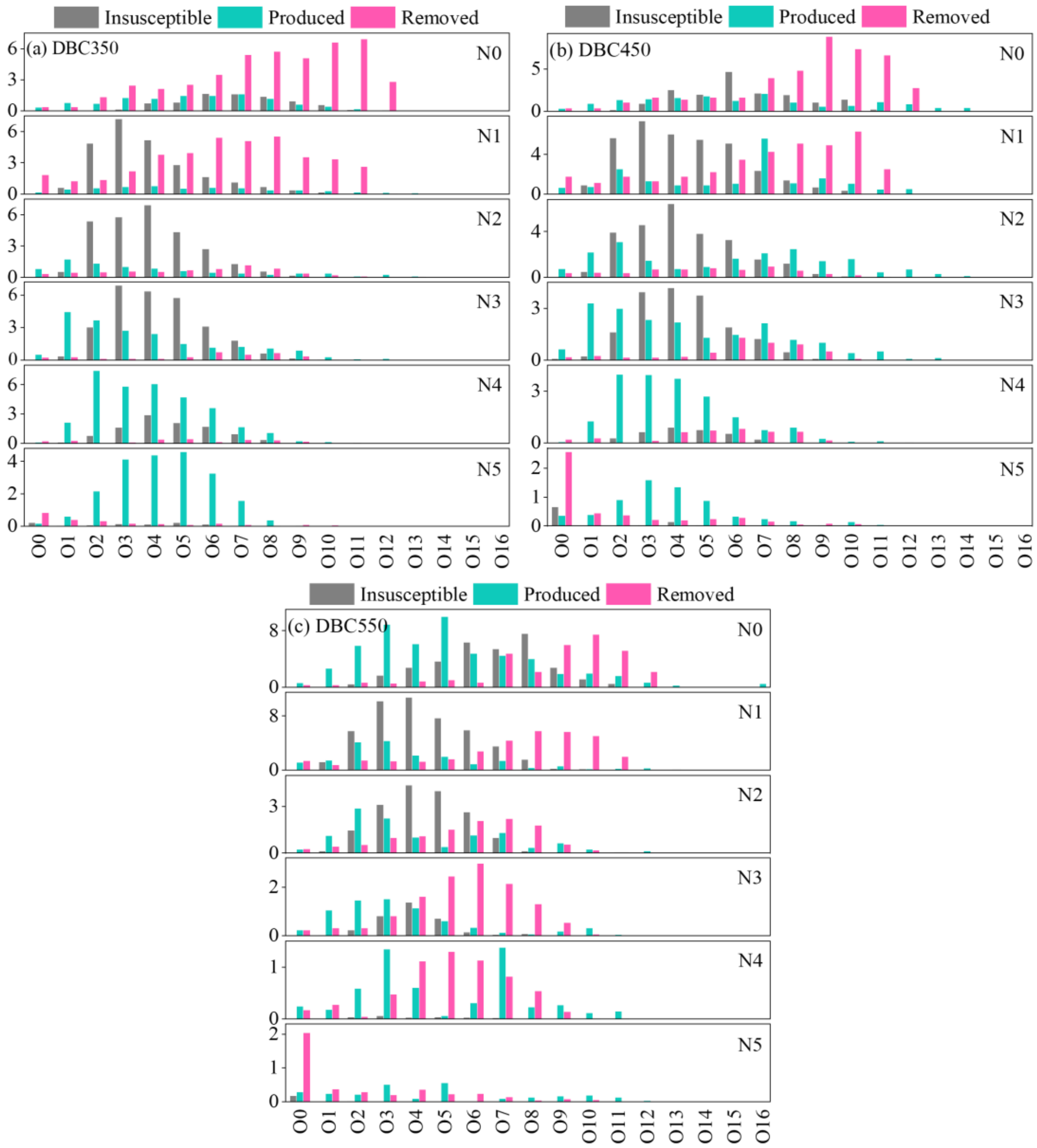

Figure S15. Changes of the relative spectral abundances of N- and O-formulae identified under +ESI during production of (a) DBC350, (b) DBC450, and (d) DBC550.

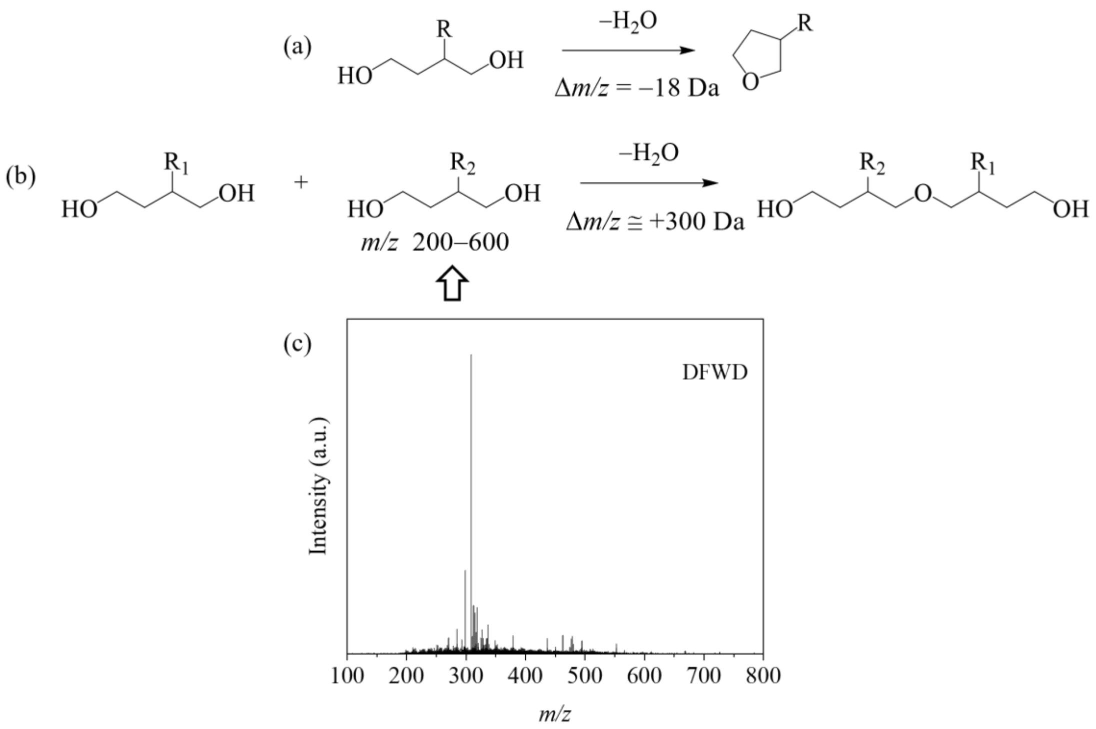

Figure S16. Schemes of possible (a) intramolecular and (b) intermolecular dehydration reactions of DFWD involved in DBC formation; (c) the negative ion mode FTICR-MS spectrum of DFWD.

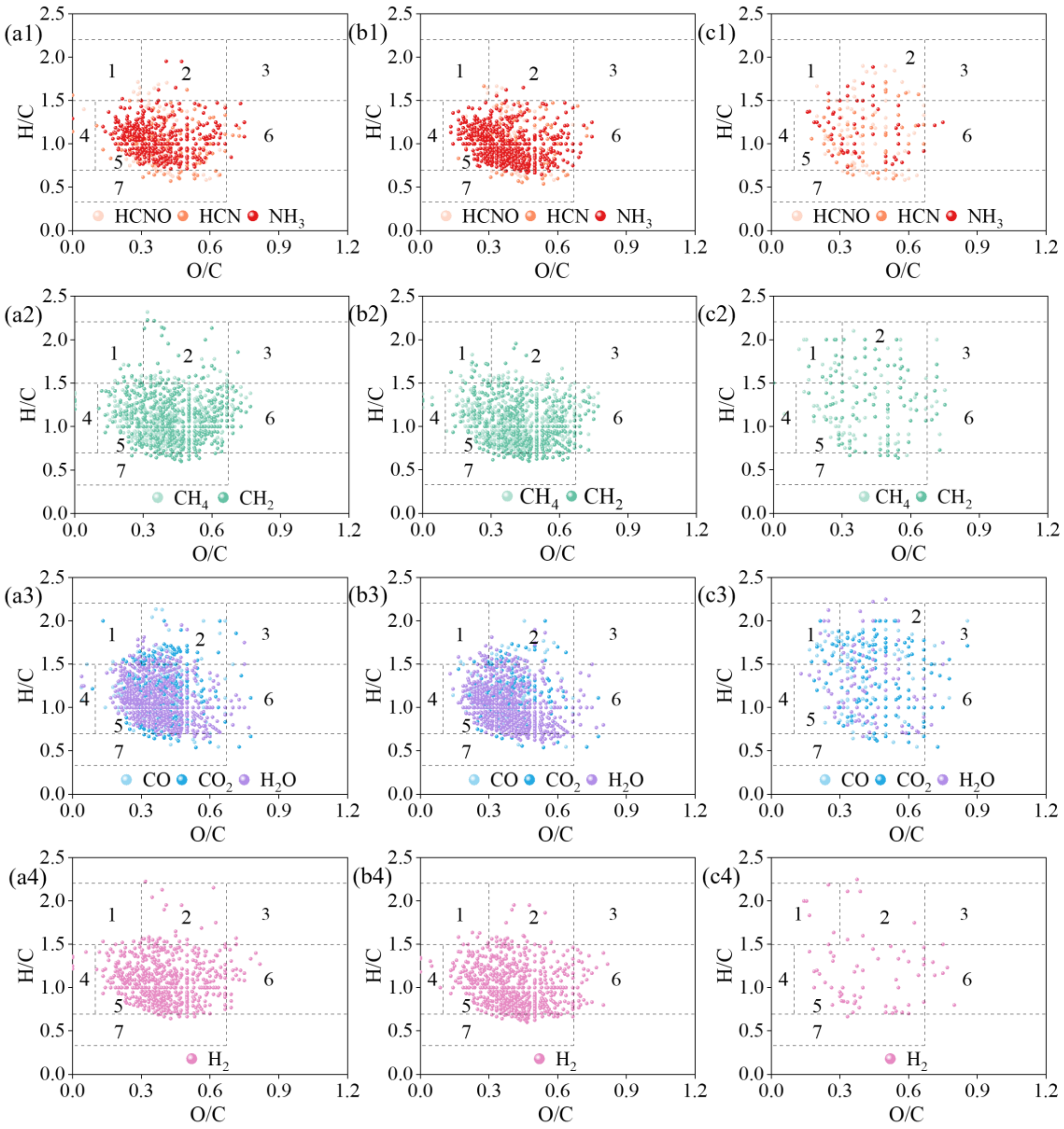

Figure S17. Van Krevelen diagrams showing the distribution of "precursors" related to possible gas-releasing reactions for (a) DBC350, (b) DBC450, and (c) DBC550 identified under –ESI. Numbers in different regions of the van Krevelen diagram represent different types of molecules as demonstrated in Note S1.7.

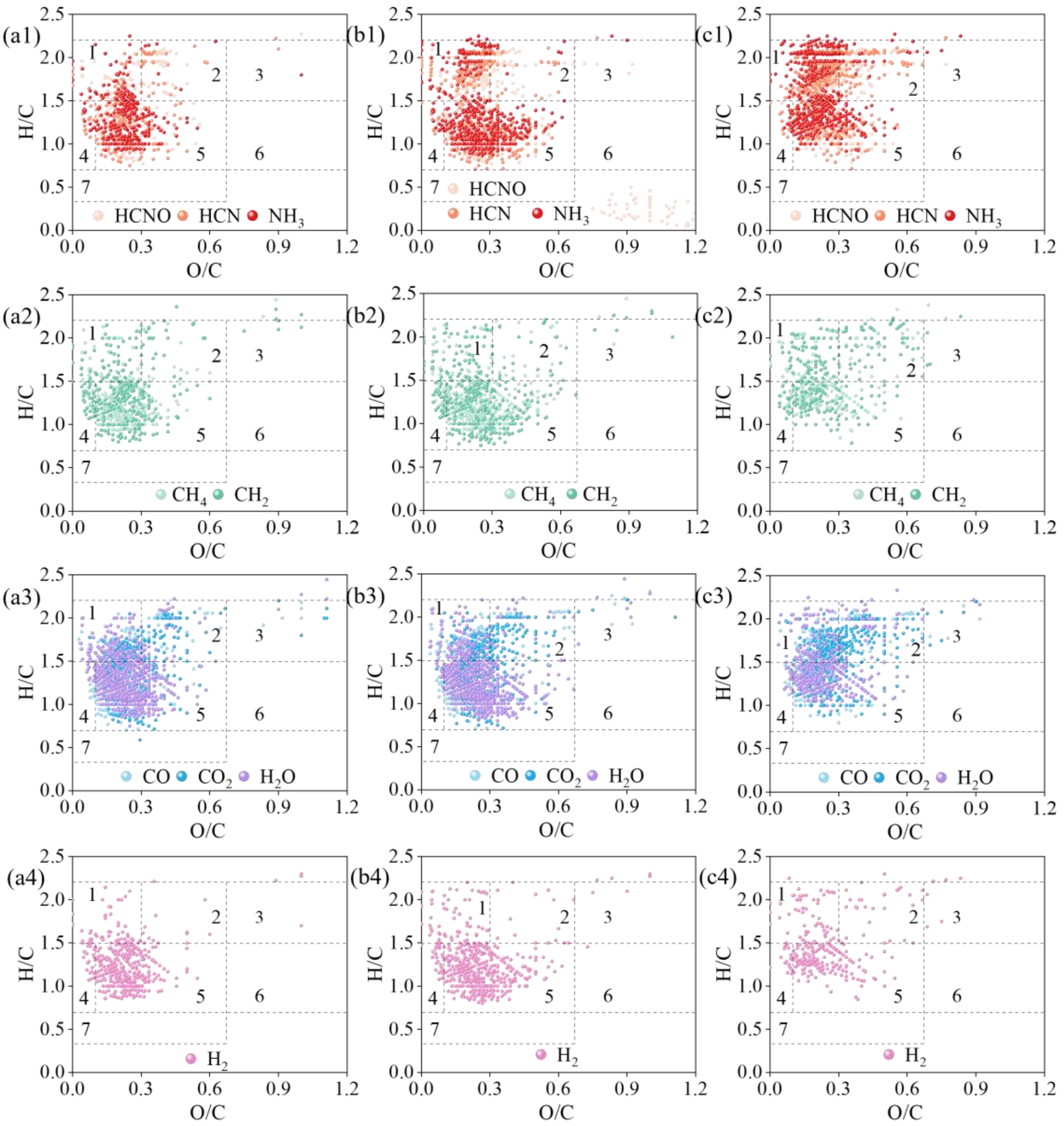

Figure S18. Van Krevelen diagrams showing the distribution of "precursors" related to possible gas-releasing reactions for (a) DBC350, (b) DBC450, and (c) DBC550 identified under +ESI. Numbers in different regions of the van Krevelen diagram represent different types of molecules as demonstrated in Note S1.7.

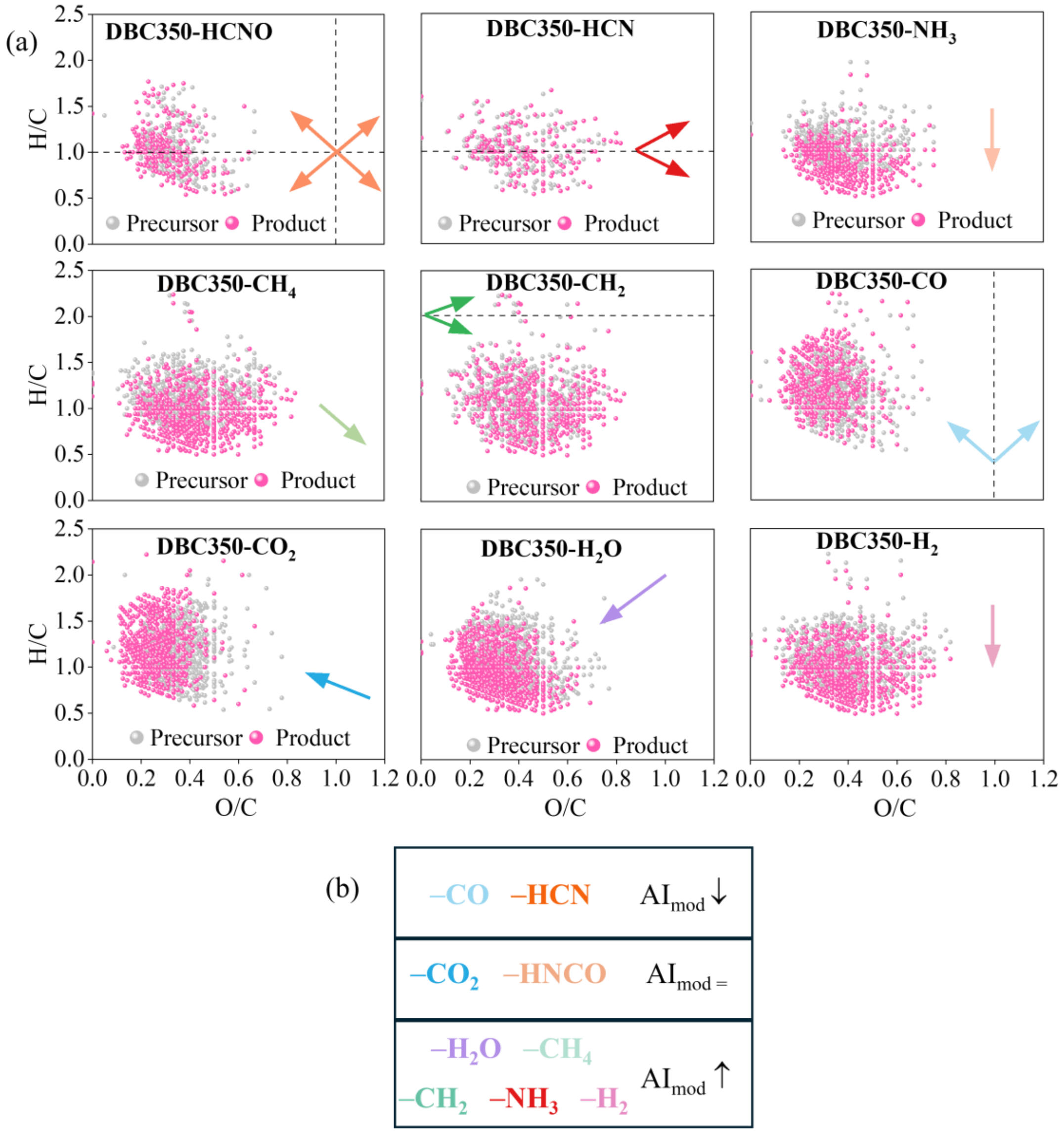

Figure S19. (a) Van Krevelen diagrams showing the distributions and reaction vectors from the "precursor" to the "product" due to possible gas-releasing reactions, and (b) changes of the AI_{mod} from the "precursor" to the "product"; reacion vectors were drawn based on analysis in Note S2.5; changes of AI_{mod} (↓: reduced, = unchanged, ↑: increased) were drawn based on the analysis in Note S2.6.

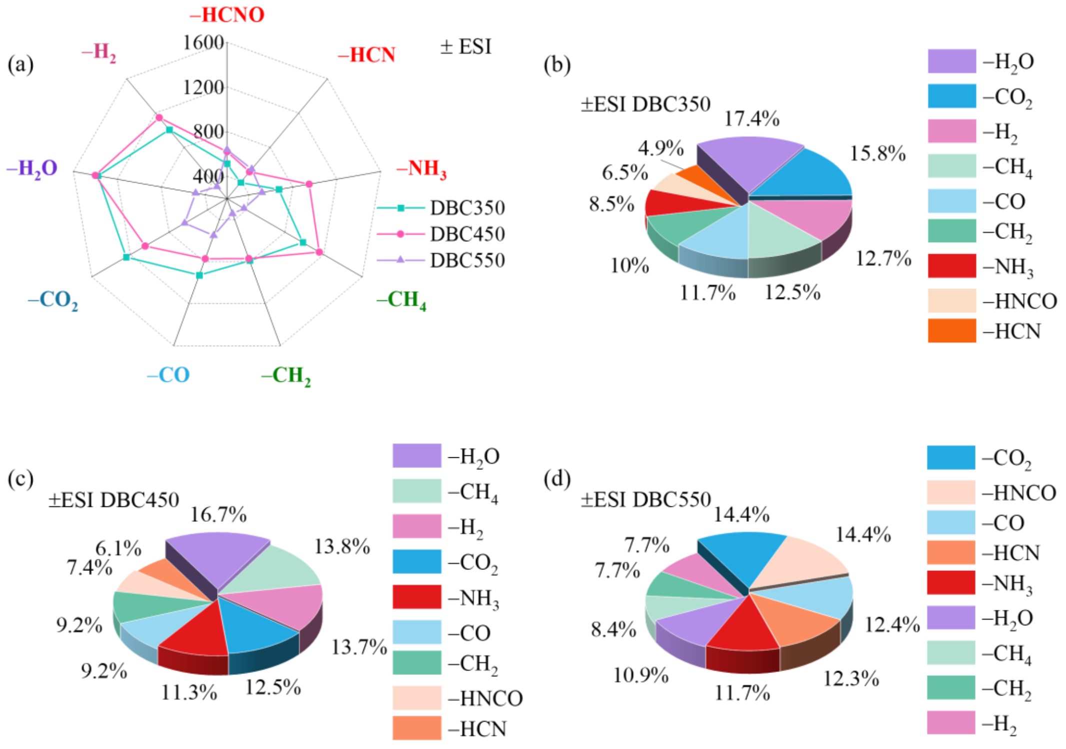

Figure S20. (a) Numbers and (b)–(d) number percentages the precursor-product pairs for DBC formulae identified under ±ESI based on possible gas-releasing reactions.

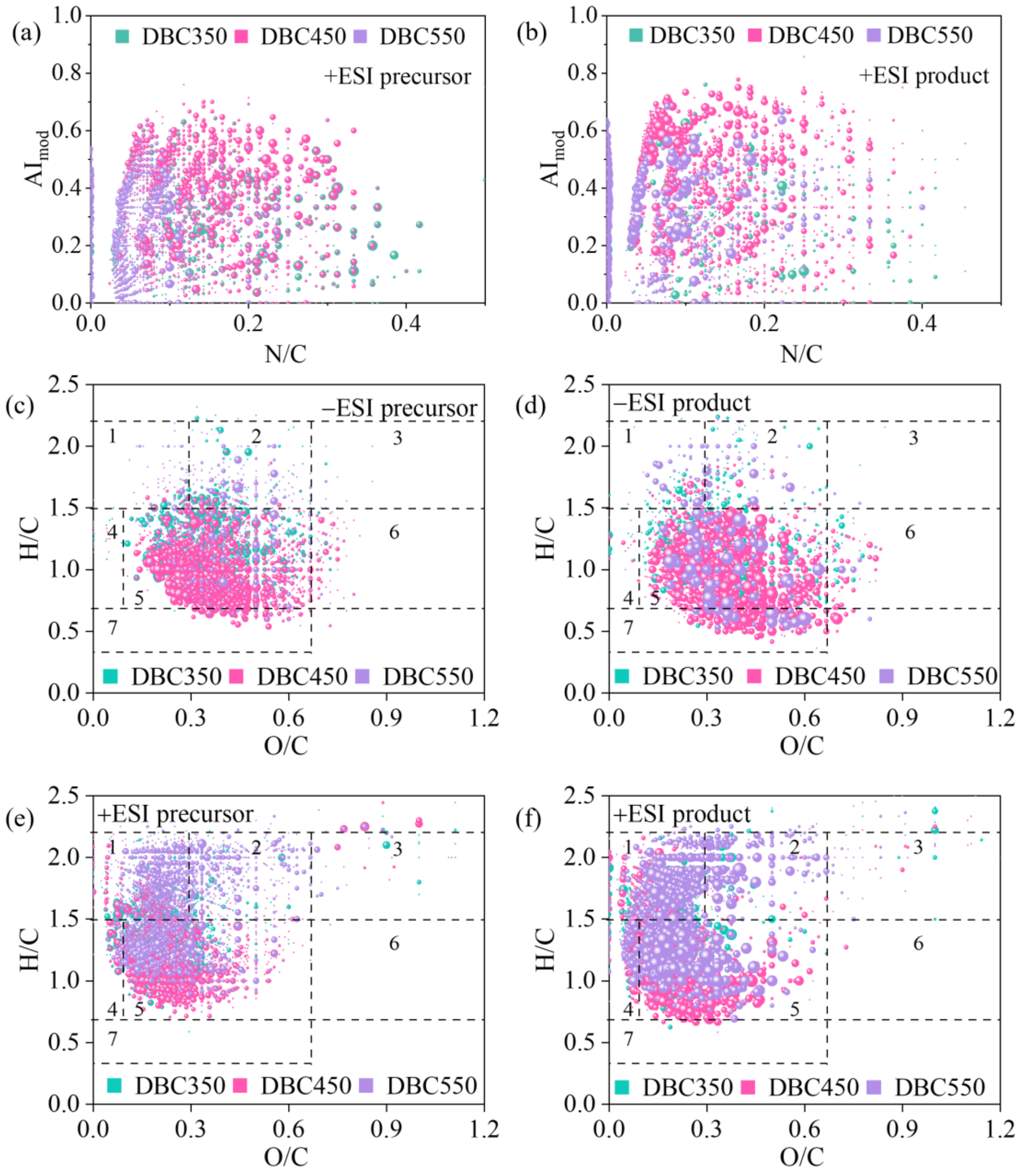

Figure S21. (a)–(b) AI_{mod} vs. N/C diagrams of precursor-product pairs for the production of DBC molecules (+ESI) and (c)–(f) van Krevelen diagrams of the precursor-product pairs for DBC molecules identified under ±ESI based on the possible gas-releasing reactions. The larger dot size represents the larger number of precursors/products involved in the reactions. Regions in the van Krevelen diagram were divided based on the Note S1.7.

Table S1. Compositions and their weight percentages of the FWD

FWD	Extractives	Hemicellulose	Cellulose	Lignin
(wt. %) [a]	67.56 ± 0.43	11.58 ± 0.04	8.82 ± 0.26	12.04 ± 0.71

[a] Calculated on a basis of total organic components without considering ash content.

Table S2. Optimized decomposition parameters of FWD pyrolysis at different heating rates

heating rate (℃· min^{-1})	components	$A_i / \sum A_i$ (%)	peak temperature (℃)	contribution to mass loss	weight loss rate (%· min^{-1})
10	hemicellulose	14.30	265.00	0.13	1.22
	proteins	53.49	310.89	0.47	2.46
	cellulose/lipids	8.76	330.00	0.08	1.44
	lignin	23.45	401.62	0.21	1.06
20	hemicellulose	13.54	276.50	0.12	2.21
	proteins	53.48	318.91	0.48	4.86
	cellulose/lipids	10.07	342.67	0.09	3.08
	lignin	22.91	414.05	0.21	2.04
30	hemicellulose	3.04	278.27	0.03	1.87
	proteins	64.47	315.92	0.59	8.17
	cellulose/lipids	10.12	348.13	0.09	4.71
	lignin	22.38	419.36	0.20	3.08
40	hemicellulose	2.04	281.84	0.02	2.11
	proteins	54.91	312.06	0.52	10.04
	cellulose/lipids	10.43	348.94	0.10	5.91
	lignin	32.62	413.47	0.31	4.56
50	hemicellulose	2.07	283.78	0.02	2.63
	proteins	51.88	312.18	0.49	12.23
	cellulose/lipids	10.62	351.73	0.10	7.76
	lignin	35.42	411.94	0.34	5.81

Table S3. Thermodynamic parameters of the FWD pyrolysis derived from the Flynn-Wall-Ozawa method at different conversion rates under the heating rate of 10 ℃·min^{-1}

α	E_α (kJ·mol^{-1})	A (s^{-1})	ΔH (kJ·mol^{-1})	ΔG (kJ·mol^{-1})	ΔS (J·mol^{-1}·K^{-1})
0.10	42.96	1.22 × 10	39.17	181.95	–236.27
0.15	111.86	2.87×10^{7}	107.57	177.14	–115.12
0.20	155.00	2.13×10^{11}	150.53	175.50	–41.33
0.25	179.96	3.55×10^{13}	175.36	174.75	1.01
0.30	199.66	1.99×10^{15}	194.96	174.23	34.29
0.35	215.50	5.02×10^{16}	210.69	173.85	60.96
0.40	236.92	3.92×10^{18}	232.01	173.37	97.03
0.45	248.16	3.85×10^{19}	243.17	173.14	115.89
0.50	266.45	1.57×10^{21}	261.37	172.78	146.59
0.55	318.97	6.53×10^{25}	313.78	171.88	234.82

Table S4. Main functional groups of gaseous products identified using TG-FTIR

Wavenumber (cm^{-1})	Selected wavenumber (cm^{-1})	Functional groups	Products	References
4000–3400	3736	O-H	H_2O, Phenols, Alcohols	[29,47]
3100–2600	2968	C-H	CH_4	
2400–2240, 730–630	2336/669	COO	CO_2 CO_2	
2060–2240	2181	CO	CO	
1900–1600	1749	C=O	Ketones, Aldehydes, Carboxylic acids, Primary amides	
1600–1450	1508	C=C	Aromatics	
1325–1000	1159	C-O/C-C	Ethers, Alcohols	
966/930	966	N-H	NH_3	[36,48]
712/2805	719	C-H/N-H	HCN	
2250/2280/2350	2280	N-H	HNCO	

Table S5. Main volatile organic compounds identified at pyrolysis temperature of 350 ℃ by GC-MS coupled NIST library (volatile organic compounds shared by 350, 450, and 550 °C, as well as those common to DBC350, DBC450, or DBC550, are highlighted in bold)

Classes	Formula	Match (%)	Time (min)	Compound names	Rel.[a] (%)
CHN	$C_7H_{17}N$	77	1.2	n-Hexylmethylamine	7.2
	$C_6H_{14}N_2$	55	7.5	Acetaldehyde, diethylhydrazone	0.9
	C_9H_9N	54	9.4	Benzenepropanenitrile	5.2
	C_8H_7N	57	8.0	Benzyl nitrile	2.0
	C_8H_7N	72	10.2	Benzene, 1-isocyano-3-methyl-	15.8
	**C_9H_9N**	**73**	**11.4**	**1H-Indole, 6-methyl-**	**3.2**
	$C_{11}H_{11}N_3$	77	17.1	2-Pyrimidinamine, 4-methyl-6-phenyl-	1.0
	$C_3H_4IN_3$	66	27.8	4-Iodo-1H-pyrazol-5-amine	3.9
					Sum: 39.4
CHNO	$C_5H_{11}NO_2$	53	0.2	Sarcosine ethyl ester hydrochloride	5.0
	**$C_{21}H_{24}N_4O_5$ [b, c]**	**62**	**30.5**	**1,3,6-Trimethyl-5-[3-(2-acetylamino-2-methoxycarbonylethyl)-1H-indol-2-yl]-1,3-dihydropyrimidin-2,4-dione**	**4.3**
	**$C_{13}H_{15}NO_2$ [b, c]**	**55**	**17.6**	**Pyracarbolid**	**1.6**
	$C_{35}H_{46}N_4O_6$	52	19.6	21H-Biline-8,12-dipropanoic acid, 3,17-diethyl-1,2,3,4,5,19,23,24-octahydro-2,7,13,18-tetramethyl-1,19-dioxo-, dimethyl ester, (2R,3R,4R)-	0.8
	$C_{34}H_{49}NO$	52	25.9	1'H-Cholest-2-eno[3,2-b]indol-6-one, 1'-methyl-, (5.alpha.)-	2.3
	$C_3H_7N_3OS$	52	5.7	Acetylthiosemicarbazide	0.9
	$C_6H_6ClNO_3S$	58	28.9	2-Chloroaniline-5-sulfonic acid	5.4
	$C_{18}H_{13}F_4N_3O$	52	8.7	Benzamide, 3-fluoro-N-[4-(3-methyl-5-trifluoromethylpyrazol-1-yl)phenyl]-	2.5
					Sum: 22.7
CHO	$C_3H_6Cl_2O$	59	2.7	2-Propanol, 1,3-dichloro-	1.7
	**C_7H_8O**	**56**	**6.9**	**p-Cresol**	**12.0**
	$C_7H_8O_2$	54	7.2	Phenol, 2-methoxy-	8.1
	$C_{20}H_{18}O_2S_3$	56	8.9	S,S'-(Thiodi-p-phenylene) bis(2-methyl-2-propenethioate)	1.0
	$C_{12}H_{16}O$	53	9.1	4-tert-Butoxystyrene	3.1
	$C_9H_{12}O_2$	68	10.0	Phenol, 4-ethyl-2-methoxy-	1.6
	**$C_{19}H_{18}O_6$ [c]**	**59**	**10.4**	**7,8,2',4'-Tetramethoxy-isoflavone**	**3.3**
	$C_{13}H_{14}F_{12}O_2$	57	13.5	Pentanoic acid, 2-methyl-, 2,2,3,3,4,4,5,5,6,6,7,7-dodecafluorohptyl ester	0.2
					Sum: 30.9
CH	CH_3Cl	51	1.6	Chloromethane	2.8
	$C_5H_{11}Br$	84	5.9	Pentane, 2-bromo-	1.1
	$C_{12}H_{18}$	63	11.1	2,5-Octadiyne, 4,4-diethyl-	0.9
	$C_{12}H_{10}S$	62	13.9	Diphenyl sulfide	2.1
					Sum: 7.0

[a] Relative abundances of compounds and compound classes at specific acquisition time.

[b] Molecular features were also detected in DBC350 under +ESI.

[c] Molecular features were also detected in DBC350 under −ESI.

Table S6. Main volatile organic compounds identified at pyrolysis temperature of 450 ℃ by GC-MS coupled NIST library (volatile organic compounds shared by 350, 450, and 550 °C, as well as those common to DBC350, DBC450, or DBC550, are highlighted in bold)

Classes	Formula	Match (%)	Time (min)	Compound names	Rel.[a] (%)
CHN	C_8H_7N	61	10.2	m-Aminophenylacetylene	17.6
	**C_9H_9N**	**56**	**11.4**	**1H-Indole, 2-methyl-**	**4.0**
	C_9H_7NS	53	11.5	2(1H)-Quinolinethione	1.3
	$C_{12}H_{12}N_2$	56	13.9	Pyridine, 4,4'-(1,2-ethanediyl)bis-	1.3
	$C_{14}H_{31}N$	53	14.0	N-Ethyl-5-propyl-5-nonanamine	1.3
	$C_{25}H_{47}NS$	52	27.4	Thiophen-2-methylamine, N,N-didecyl-	2.7
					SUM: 28.3
CHNO	$C_{22}H_{19}N_3O$	52	24.0	(3-Methoxy-phenyl)-(6-methyl-4-phenyl-quinazolin-2-yl)-amine	1.4
	**$C_{22}H_{27}N_3O$** [b]	**52**	**27.3**	**Acetamide, 2-(adamantan-1-yl)-N-(2,3-dimethylquinoxalin-6-yl)-**	**2.2**
	$C_{22}H_{18}N_2O_3$	52	27.8	Benzhydrazide, 2-benzoyl-N2-(4-methylbenzoyl)-	3.0
	**$C_3H_7NO_2$** [b]	**63**	**0.3**	**Alanine**	**7.9**
	$C_{25}H_{41}NO_3$	52	6.3	Glutaric acid, monoamide, N-(2-phenylpropyl)-, undecyl ester	1.6
	**$C_{14}H_{19}NO_4$** [b, c]	**69**	**8.2**	**Glycine, N-benzyloxycarbonyl-N-ethyl-, ethyl ester**	**2.4**
	$C_{32}H_{54}N_2O_2$	55	10.7	N-Behenoyl-5-hydroxytryptamine	1.9
	$C_8H_{11}NO_2$	50	10.9	2-Cyclohexen-3-ol-1-one, 2-[1-iminoethyl]-	1.2
	$C_9H_7NO_2S$	63	17.6	2-Propenenitrile, 3-(phenylsulfonyl)-	2.7
	$C_{24}H_{22}ClNO$	55	24.0	JWH 398 8-chloronaphthyl isomer	0.8
	$C_8H_7N_3OS$	52	31.2	2-Aminobenzothiazol-6-carboxamide	7.3
					SUM: 32.5
CHO	$C_{23}H_{18}O_4$	58	4.2	Propenone, 3-(4-benzyloxyphenyl)-1-(3,4-methylenedioxyphenyl)-	0.8
	**C_7H_8O**	**68**	**7.0**	**p-Cresol**	**11.9**
	$C_{12}H_{15}F_3O$	59	9.1	3,4,5-Trifluorobenzyl alcohol, 2-methylbutyl ether	1.0
	$C_{29}H_{26}O_4$	60	13.7	Phenylacetic acid, 3,5-dibenzyloxy-, benzyl ester	0.8
	$C_5HF_{11}O$	52	28.5	1,1,1,2,2,3,3-Heptafluoro-3-(1,2,2,2-tetrafluoroethoxy)propane	4.7
	$C_{13}H_{10}FO_3P$	60	31.3	10H-Phenoxaphosphine, 2-fluoro-10-hydroxy-8-methyl-, 10-oxide	2.8
					SUM: 22.0
CH	C_2HBr	51	4.9	Bromoacetylene	1.1
	C_9H_{12}	63	5.3	Benzene, 1,2,3-trimethyl-	1.6
	$C_{10}H_{22}$	67	7.3	Heptane, 2,3,5-trimethyl-	2.3
	$C_{10}H_{10}$	53	8.1	Naphthalene, 1,2-dihydro-	4.2
	$C_{11}H_{10}$	57	10.2	Naphthalene, 2-methyl-	2.5
	$C_{12}H_{10}S$	73	17.1	Diphenyl sulfide	1.7
	$C_{24}H_{22}$	51	28.8	1,2,3-Triphenyl-3-isopropyl-cyclopropene	3.9
					SUM: 17.3

[a] Relative abundances of compounds and compound classes at specific acquisition time.

[b] Molecular features were also detected in DBC450 under +ESI.

[c] Molecular features were also detected in DBC450 under –ESI.

Table S7. Main volatile organic compounds identified at pyrolysis temperature of 550 ℃ by GC-MS coupled NIST library (volatile organic compounds shared by 350, 450, and 550 °C, as well as those common to DBC350, DBC450, or DBC550, are highlighted in bold)

Classes	Formula	Match (%)	Time (min)	Compound names	Rel.[a] (%)
CHN	**C_9H_9N**	**64**	**11.4**	**1H-Indole, 6-methyl-**	**3.3**
	C_8H_7N	52	10.2	m-Aminophenylacetylene	6.5
	$C_8H_{13}N_7$	53	4.1	1,2,4-Triazol-3-amine, 5-(1,3,5-trimethyl-4-pyrazolyl) amino-	0.5
	$C_7H_{17}N$	60	1.2	n-Hexylmethylamine	9.8
	$C_{15}H_{13}N$	72	23.8	Benzenamine, N-(3-phenyl-2-propenylidene)-	1.6
	$C_{10}H_{10}N_2$	52	2.9	1H-Imidazole, 1-(phenylmethyl)-	1.1
					SUM: 22.9
CHNO	$C_7H_7NO_2$	62	1.2	3-Hydroxypyridine monoacetate	0.8
	$C_7H_7Br_2NO$	54	5.4	phenol, 4-amino-2,6-dibromo-3-methyl-	0.7
	$C_6H_7IN_2O_2$	51	7.8	Uracil, 1,3-dimethyl-5-iodo-	1.0
	$C_5Cl_2F_3N$	50	26.9	Pyridine, 3,5-dichloro-2,4,6-trifluoro-	3.5
	$C_{24}H_{32}F_5NO_3$	54	30.4	L-Proline, N-(pentafluorobenzoyl)-, dodecyl ester	3.2
	$C_{23}H_{17}NO_9$	60	1.7	4H-Pyrido[3,2,1-jk]carbazole-1,2,3,6-tetracarboxylic acid, 4-oxo-, tetramethyl ester	2.1
	$C_{17}H_{26}F_3N_3O_3$	51	24.5	Cyclohexanepropanamide, N-[1-(2-methylpropyl)-2,5-dioxo-4-(trifluoromethyl)-4-imidazolidinyl]-	0.9
	$C_{16}H_{20}F_3NO_4$	59	22.3	1H-Indole-2,4-dione, 3-hydroxy-6,6-dimethyl-1-(tetrahydrofuran-2-ylmethyl)-3-trifluoromethyl-3,5,6,7-tetrahydro-	0.8
	$C_{16}H_{14}N_2O$	51	24.2	2,3-Dihydro-7-methyl-4-phenyl-1H-1,5-benzodiazepin-2-one	1.1
	$C_{16}H_{10}N_4O_2S$	53	30.8	2,6-Diphenyl-1,3,4-thiadiazolo[3,2-a](1,3,5)triazine-5,7-dione	5.4
	$C_{16}H_{10}ClNO_2$	55	31.9	3-Anilino-2-chloro-1,4-naphthoquinone	3.8
	$C_{14}H_{24}N_2O_2S$	56	31.0	Piperazine, 1-(bicyclo[2.2.1]hept-5-en-2-ylmethyl)-4-ethanesulfonyl-	2.9
	$C_{13}H_7F_2NO_4$	52	10.2	2,6-Difluorobenzoic acid, 4-nitrophenyl ester	1.1
	**$C_{12}H_{17}NO_2$ [b]**	**69**	**8.3**	**Acetamide, 2-(4-methoxyphenyl)-N-propyl-**	**0.7**
	**$C_{10}H_{12}N_2O$ [b]**	**51**	**29.3**	**Pyridine-3-carbonitrile, 1-ethyl-4,6-dimethyl-2-oxo-1,2-dihydro-**	**6.0**
	$C_{10}H_{10}N_4O_2S$	67	13.9	Sulfadiazine	2.5
	$C_{10}H_{10}N_4O_2S$	56	17.1	Sulfadiazine	1.4
	$C_{10}H_{10}N_2OS$	50	24.7	3-Imidazoline-2-thione, 5-methyl-4-phenyl-, 3-oxide	2.0
					SUM: 39.8
CHO	**C_7H_8O**	**59**	**7.0**	**p-Cresol**	**3.2**
	$C_{22}H_{18}O_4$	55	9.1	1,3-Benzenediol, O,O'-di(2-methylbenzoyl)-	1.5
	$C_{13}H_{11}F_5O_5$	54	12.7	Benzeneacetic acid, 3-methoxy-4-(2,2,3,3,3-pentafluoro-1-oxopropoxy)-, methyl ester	3.2
	$C_{12}H_{10}O_2S$	56	17.6	Diphenyl sulfone	2.8
	$C_{16}H_9F_3O_2$	51	28.8	9-phenanthrol, trifluoroacetate ester	4.9
	$C_{16}H_{18}F_4O_4$	52	29.5	Succinic acid, 2-fluoro-6-(trifluoromethyl)benzyl isobutyl ester	4.6
	$C_{24}H_{22}O_4$	51	29.9	Terephthalic acid, bis-(4-ethyl-phenyl ester	8.9
	**$C_{17}H_{12}O_3$ [b]**	**51**	**30.3**	**2-Furoic acid, 4-biphenyl ester**	**5.6**
					SUM: 34.6
CH	$C_{12}H_{10}S$	64	13.9	Diphenyl sulfide	2.7
					SUM: 2.7

[a] Relative abundances of compounds and compound classes at specific acquisition time.

[b] Molecular features were also detected in DBC550 under +ESI.

Table S8. Generalized 2D-TG-FTIR-COS characteristics and the sequential temperature responses of gaseous products during FWD pyrolysis

*v*2 (cm^{-1}) Assig.	Position *v*1 (cm^{-1}) and signals									Sequential temperature responses
	3736 O-H	2336 CO_2	1749 C=O	1159 C-O	2110 CO	2968 CH_4	1508 C=C	966 NH_3	719 HCN	
3736		+/–	+/–							H_2O, phenols > carboxylic acids/ketones, CO_2
2336	+/+		+/+	+/+		+/–	+/+	+/–	+/–	Carboxylic acids/ketones, ethers, aromatics > CO_2 > HCN, NH_3, CH_4
1749	+/+	+/–		+/–		+/–	+/–	+/–	+/–	Carboxylic acids/ketones >ethers, aromatics
1159	+/+	+/–	+/+			+/–		+/–	+/–	Ethers > CO_2, CH_4, NH_3, HCN
2110	–/–	–/–	–/–	–/–		–/–	–/–	–/–		All gases > CO
2968	+/+	+/+	+/+	+/+	–/+	+/+	+/+	+/+		HCN, NH_3 > CH_4 > CO
1508		+/–		+/+				+/–	+/–	Ethers > aromatics > CO_2, HCN, NH_3
933	+/+	+/+	+/+	+/+		+/–				NH_3 > CH_4
719						+/–				HCN > CH_4

Note: "+" and "–" represent the positive (red colors) and negative (blue colors) correlations, respectively, in the synchronous and asynchronous maps of the generalized 2D-FTIR-COS for gases (Figure 2a).

Table S9. Main functional groups identified in FTIR spectra of biochar, DBCs, and BCAs

Wavenumber (cm^{-1})	Functional groups	Vibration	Possible products	References
4000–3700	O-H	stretching	Minerals	[29,44,47,49,50]
3700–3300	O-H	stretching	Phenols, alcohols	
3000-3300	N-H	stretching	Heterocyclic nitrogen compounds	
3000–2800	C-H	stretching	Aliphatic structures	
2800–2000	-C≡C/-C=C=O/ C≡N/C=C=N	stretching	Alkyne/Ketene/Nitrile/heterocyclic nitrogen compounds	
1900–1680	C=O	stretching	Ketones, aldehydes, acids	
1790–1760*	C=O	Stretching	Acids	
1760–1730*	C=O	Stretching	Aldehydes	
1730–1680*	C=O	Stretching	Ketones	
1680–1600	C=O/N-C=O	stretching	Amides	
1600–1450	C=C	stretching	Aromatics	
1450–1350	C−H	bending	Hydrocarbons/Alkane	
1350–1000	C-O/C-N	stretching	Ethers, alcohols, amines	
1350–1250*	C-N/C-O-C	stretching	Aryl amines, aryl ethers	
1250–1130*	C-N	stretching	Esters and amines	
1130-1085*	C-O	stretching	Aliphatic ethers	
1085-1050*	C-O/C-N	stretching	Primary alcohols, amines	
1050–1000*	C-O-C/	stretching	Acid derivatives, ethers, phosphates	
1000–650	C-H	bending	Benzene derivatives/olefins	

Table S10. Generalized 2D-FTIR-COS characteristics and the sequential temperature responses of biochar functional groups

Sample	Cross-region range ($v1/v2$, cm^{-1})	Signals (Synchronous/ Asynchronous)	Primary sequential temperature responses of wavenumbers (cm^{-1})	Primary sequential temperature responses of functional groups
Biochar	1100–1680/2200–3600	（+/+）	1680–1750 → 1600–1680 → 1050–1600 → 2800–3600 → 2000–2800	Carbonyl in aldehydes/ketones/carboxylic acids → Amide groups → Alcoholic/ether/amine groups; hydrocarbon and aromatic structures → Aliphatic -CH_2-, heterocyclic nitrogen structures, phenolic -OH→ Unsaturated structures in carbon and nitrogen compounds
	1100–1680/1680–1750	（+/–）		
	1600–1680/1050–1600	（+/+）		
	2000–2800/2800–3600	（+/–）		
	850–936/2800–3600	(–/–)		

Note: "+" and "–" represent the positive (red colors) and negative (blue colors) correlations, respectively, in the synchronous and asynchronous maps of the generalized 2D-FTIR-COS for biochar (Figure 2a).

Table S11. Hetero 2D-TG-FTIR/FTIR-COS characteristics and the relationships between the sequential temperature responses of gases and biochar functional groups

Sample	Cross-region range (*v*1/*v*2, cm^{-1})	Signals (Synchronous/ Asynchronous)	Primary sequential temperature responses of wavenumbers (cm^{-1})	Primary sequential temperature responses of gases and BC functional groups
Gases vs. biochar	960–1110/ (650–1800, 2336, 3335, 3736)	(–/–)	960–1110 (biochar) → (650–1800, 2336, 3335, 3736) (gases); (1650–1780, 1829–2739) (biochar) → (650–1800, 2336, 2968, 3335, 3736) (gases) → (1230–1500, 1520–1600, 2850–3560) (biochar); 2968 (gases) → 2200–2900 (biochar) → 2100 (gases)	Alcoholic/aliphatic ether/amine groups (biochar) → All gases expect CH_4 and CO; Carbonyl in ketones/ aldehydes/carboxylic acids, unsaturated structures in carbon and nitrogen compounds (biochar) → All gases except CO → Aromatic structures, -CH_3/-CH_2-/-CH groups, heterocyclic structures, phenolic -OH, aromatic amine groups (biochar); Gaseous CH_4 → Unsaturated structures in carbon and nitrogen compounds (biochar) → Gaseous CO
	1230–1500/ (650–1800, 2336, 2968, 3335, 3736)	(+/–)		
	1520–1600/ (650–1800, 2336, 2968, 3335, 3736)	(+/–)		
	1650–1780/ (650–1800, 2336, 2968, 3335, 3736)	(+/+)		
	1829–2739/ (650–1800, 2336, 3335, 3736)	(–/–)		
	2850–3560/ (650–1800, 2336, 2968, 3335, 3736)	(+/–)		
	2200–2900/ 2968	(+/–)		
	(2150–3600)/ (2181, 2100)	(+/–)		

Note: "+" and "–" represent the positive (red colors) and negative (blue colors) correlations, respectively, in the heterosynchronous and heteroasynchronous maps of the Hetero 2D-FTIR-COS for Gases vs. biochar (Figure 2b).

Table S12. Hetero 2D-TG-FTIR/FTIR-COS characteristics and the relationships between the sequential temperature responses of functional groups in BCAs and DBCs

Sample	Cross-regions ($v1/v2$, cm^{-1})	Signals (Synchronous/ Asynchronous)	Primary sequential temperature responses of wavenumbers (cm^{-1})	Primary sequential temperature responses of functional groups
BCAs vs. DBCs	(650–800, 1050–1220, 1350–1550, 1610–1680, 3110–3660)/ (650–750, 980–1750, 2800–3660)	(–/+)	(650–750, 980–1750, 2800–3660) (BCAs) → (650–800, 1050–1220, 1350–1550, 1610–1680, 3110–3660) (DBCs)	Aromatic structures, alcoholic/ aliphatic ether/ ester/ amine/ aliphatic hydrocarbon/ amide/ketone/ aldehydes/ carboxylic/ alkane/ heterocyclic structures/ phenolic groups (BCAs) → Aromatic structures/ alcoholic/ aliphatic ether/ ester/ amine/ hydrocarbon/ amide/ heterocyclic structures/ phenolic groups (DBCs)

Note: "+" and "–" represent the positive (red colors) and negative (blue colors) correlations, respectively, in the heterosynchronous and heteroasynchronous maps of the Hetero 2D-FTIR-COS for BCAs vs. DBCs (Figure 2f).

Table S13. Generalized 2D-FTIR-COS characteristics and the sequential temperature responses of DBC functional groups

Sample	Cross-region range ($v1/v2$, cm^{-1})	Signals (Synchronous/ Asynchronous)	Primary sequential temperature responses of wavenumbers (cm^{-1})	Primary sequential temperature responses of functional groups
DBCs	1030–1240/3150–3650	(+/–)	1645 → 3400–3700 → 3000–3350 → 1000–1250; 1067 → 1123	Amide groups → Phenolic groups → Heterocyclic structures→ Amine/ acidic/ alcoholic groups → Aliphatic ether groups
	1067/1123	(+/+)		
	3100–3600/1645	(+/–)		
	1000–1230/1645	(+/–)		
	3060–3332/3420–3700	(+/–)		
	3200–3600/1000–1231	(+/+)		

Note: "+" and "–" represent the positive (red colors) and negative (blue colors) correlations, respectively, in the synchronous and asynchronous maps of the generalized 2D-FTIR-COS for DBCs (Figure 2f)

Table S14. n_C^0 and b values of saturation mass concentration for different compound classes

Classes	n_C^0	b_C	b_O	b_{CO}	b_N	b_S
CH	23.80	0.4861				
CHO	22.66	0.4481	1.656	–0.7790		
CHN	24.59	0.4066			0.9619	
CHON	24.13	0.3667	0.7732	–0.0779	1.114	
CHOS	24.06	0.3637	1.327	–0.3988		0.7579
CHONS	28.50	0.3848	1.011	0.2921	1.053	1.316

Note: Parameters were obtained by least-squares optimization using the database of NCI.[1]

Table S15. Intensity-weighted molecular parameters of DFWD and DBC samples

	O/C_w	H/C_w	N/C_w	DBE_w	$AI_{mod,w}$
			−ESI		
DFWD	0.41	1.43	0.04	6.32	0.23
DBC350	0.38	1.21	0.10	8.76	0.31
DBC450	0.42	1.15	0.06	8.90	0.34
DBC550	0.39	1.43	0.05	6.86	0.24
			+ESI		
DFWD	0.30	1.66	0.06	5.00	0.21
DBC350	0.23	1.46	0.14	7.60	0.29
DBC450	0.26	1.47	0.10	7.10	0.29
DBC550	0.26	1.57	0.05	5.86	0.25

Table S16. Intensity-weighted molecular parameters of the “produced”, “removed” and “insusceptible” molecules

	O/C_w	H/C_w	N/C_w	DBE_w	$AI_{mod,w}$
			DBC350 in –ESI		
Produced	0.32	1.26	0.13	9.70	0.31
Removed	0.38	1.55	0.02	5.97	0.16
Insusceptible	0.41	1.19	0.09	8.40	0.31
			DBC450 in –ESI		
Produced	0.37	1.11	0.08	10.75	0.40
Removed	0.39	1.56	0.04	5.59	0.16
Insusceptible	0.43	1.16	0.05	8.43	0.32
			DBC550 in –ESI		
Produced	0.42	1.45	0.08	8.38	0.24
Removed	0.40	1.47	0.05	6.63	0.27
Insusceptible	0.37	1.41	0.03	5.73	0.24
			DBC350 in +ESI		
Produced	0.19	1.41	0.16	9.31	0.29
Removed	0.31	1.70	0.04	5.07	0.16
Insusceptible	0.24	1.48	0.12	6.66	0.29
			DBC450 in +ESI		
Produced	0.24	1.43	0.11	8.70	0.31
Removed	0.32	1.76	0.05	4.61	0.15
Insusceptible	0.26	1.48	0.09	6.54	0.28
			DBC550 in +ESI		
Produced	0.23	1.60	0.05	6.48	0.30
Removed	0.32	1.69	0.07	5.14	0.21
Insusceptible	0.28	1.64	0.05	4.89	0.22

Table S17. Ultimate and organic carbon analysis performed on bulk FWD (wt.% dry basis)

C [%]	H [%]	N [%]	S [%]	TOC [%]	WEOC [%]	WEOC/TOC
35.32	2.29	3.38	1.58	34.64	24.35	70.3%

Table S18. Summary of the transformation reactions related to possible primary gas releases

Reactions	Formula difference	Example reactions from references	References
	$-H_2O$	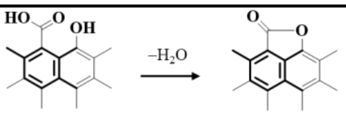	51
	$-H_2O$	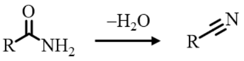	52
	$-H_2O$	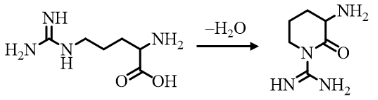	53
Dehydration	$-H_2O$	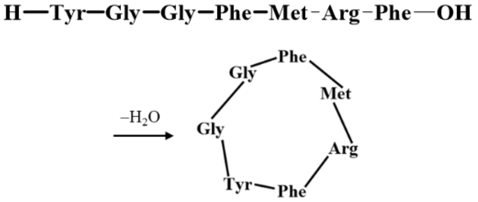	54
	$-H_2O$	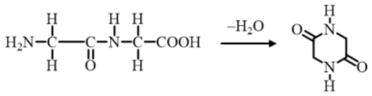	55
	$-H_2O$	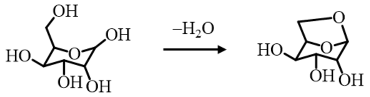	56
	$-CO_2$	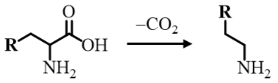	57
Decarboxylation	$-CO_2$	Stearic acid $\xrightarrow{-CO_2}$ unsaturated fatty acids	58
	$-CO_2$	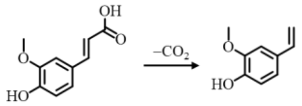	59
Deamidization	–HNCO	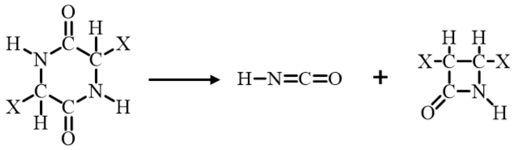	48
Dehydrocyanide	–HCN	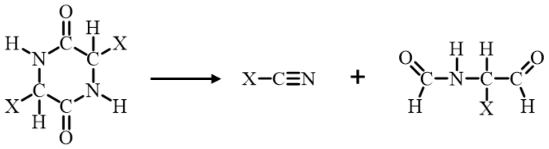	48
Deamination	$-NH_3$	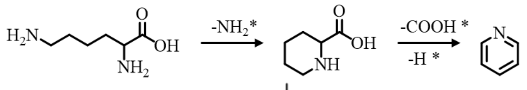	52
Demethylation	$-CH_2/CH_4$	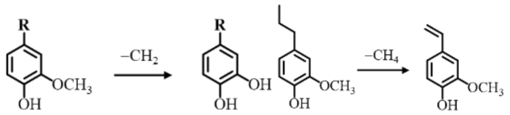	59,60
Dehydrogenation	$-H_2$	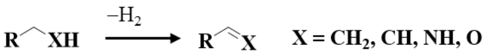	61
Decarbonylation	–CO	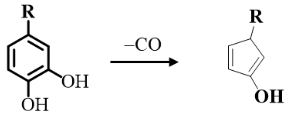	62

Table S19. Intensity-weighted molecular parameters of "precursors" involved in possible gas-releasing reactions during DBC production (–ESI)

Reactions	O/C_w	H/C_w	N/C_w	DBE_w	$AI_{mod,w}$	MW_w
			DBC350			
–HNCO	0.35	1.05	0.11	10.79	0.38	377.88
–HCN	0.40	1.04	0.12	10.58	0.40	383.58
$-NH_3$	0.38	1.05	0.11	10.52	0.41	379.67
$-CH_4$	0.40	1.15	0.09	9.22	0.34	368.52
$-CH_2$	0.43	1.14	0.11	9.38	0.34	381.25
–CO	0.33	1.26	0.12	8.69	0.30	364.05
$-CO_2$	0.36	1.26	0.10	8.31	0.28	356.65
$-H_2O$	0.37	1.12	0.09	9.70	0.37	367.57
$-H_2$	0.39	1.12	0.09	9.57	0.36	371.93
			DBC450			
–HNCO	0.36	1.02	0.11	11.16	0.43	384.05
–HCN	0.41	1.02	0.11	10.78	0.40	394.76
$-NH_3$	0.38	1.01	0.09	10.86	0.44	379.62
$-CH_4$	0.41	1.08	0.07	9.75	0.38	371.69
$-CH_2$	0.41	1.25	0.10	8.48	0.25	390.71
–CO	0.36	1.17	0.11	9.51	0.35	367.67
$-CO_2$	0.35	1.23	0.08	8.45	0.30	351.16
$-H_2O$	0.38	1.06	0.07	10.13	0.40	369.99
$-H_2$	0.41	1.05	0.07	10.19	0.40	381.06
			DBC550			
–HNCO	0.43	1.18	0.11	6.68	0.36	260.14
–HCN	0.41	1.18	0.11	6.52	0.38	250.50
$-NH_3$	0.40	1.15	0.10	7.41	0.36	279.87
$-CH_4$	0.41	1.35	0.02	5.49	0.29	262.39
$-CH_2$	0.44	1.35	0.03	5.21	0.30	252.31
–CO	0.42	1.46	0.03	4.44	0.24	254.88
$-CO_2$	0.42	1.46	0.01	4.63	0.22	276.72
$-H_2O$	0.43	1.34	0.03	5.70	0.28	268.87
$-H_2$	0.43	1.25	0.03	6.51	0.34	295.92

Table S20. Intensity-weighted molecular parameters of "precursors" involved in possible gas-releasing reactions during DBC production (+ESI)

Reactions	O/C_w	H/C_w	N/C_w	DBE_w	$AI_{mod,w}$	MW_w
–HNCO	0.26	1.46	0.11	7.06	0.32	382.07
–HCN	0.25	1.47	0.09	7.35	0.30	390.74
–NH_3	0.23	1.42	0.09	7.95	0.28	399.82
–CH_4	0.22	1.36	0.11	8.46	0.30	374.91
–CH_2	0.22	1.60	0.10	5.85	0.31	364.88
–CO	0.29	1.61	0.10	5.39	0.28	368.42
–CO_2	0.32	1.65	0.09	4.83	0.28	399.28
–H_2O	0.24	1.37	0.12	8.13	0.32	367.33
–H_2	0.22	1.30	0.12	9.19	0.33	385.83
			DBC450			
–HNCO	0.27	1.49	0.10	6.29	0.32	347.74
–HCN	0.25	1.54	0.08	6.02	0.31	350.83
–NH_3	0.26	1.46	0.09	6.93	0.34	356.07
–CH_4	0.26	1.41	0.09	7.69	0.32	372.17
–CH_2	0.23	1.48	0.09	6.86	0.32	363.33
–CO	0.26	1.50	0.10	6.36	0.28	343.35
–CO_2	0.30	1.58	0.09	5.41	0.27	356.55
–H_2O	0.25	1.37	0.11	7.94	0.33	358.57
–H_2	0.25	1.34	0.10	8.45	0.34	376.90
			DBC550			
–HNCO	0.28	1.68	0.06	4.82	0.22	382.43
–HCN	0.27	1.74	0.06	4.35	0.22	378.18
–NH_3	0.20	1.82	0.06	3.54	0.26	360.11
–CH_4	0.32	1.72	0.02	4.32	0.25	405.85
–CH_2	0.30	1.72	0.03	4.51	0.25	381.99
–CO	0.33	1.74	0.03	3.77	0.22	354.60
–CO_2	0.36	1.78	0.03	3.31	0.22	381.48
–H_2O	0.31	1.68	0.03	4.74	0.28	410.88
–H_2	0.32	1.70	0.03	4.69	0.26	413.98

Table S21. Reaction vectors from the "precursor" to the "product" in the van Krevelen diagram based on the possible gas-releasing reactions

Process	(x_f, y_f)	$(\Delta x, \Delta y)$	Reaction vectors
$-HNCO$	$\left(\frac{O-1}{C-1},\frac{H-1}{C-1}\right)$	$(\frac{O/C-1}{C-1},\frac{H/C-1}{C-1})$	$if\ H/C<1, O/C<1$ $(\Delta y<0, \Delta x<0)$<br>$if\ H/C>1, O/C>1$ $(\Delta y>0, \Delta x>0)$<br>$if\ H/C>1, O/C<1$ $(\Delta y>0, \Delta x<0)$<br>$if\ H/C<1, O/C>1$ $(\Delta y<0, \Delta x>0)$
$-HCN$	$\left(\frac{O}{C-1},\frac{H-1}{C-1}\right)$	$(\frac{O/C}{C-1},\frac{H/C-1}{C-1})$	$if\ H/C>1\ (\Delta y>0,\ \Delta x>0)$<br>$if\ H/C<1\ (\Delta y<0,\ \Delta x>0)$
$-NH_3$	$\left(\frac{O}{C},\frac{H-3}{C}\right)$	$(0, \frac{-3}{C})$	$\Delta y<0,\ \Delta x=0$
$-CH_4$	$\left(\frac{O}{C-1},\frac{H-4}{C-1}\right)$	$(\frac{O/C}{C-1},\frac{H/C-4}{C-1})$	$if\ H/C>4\ (\Delta y>0,\ \Delta x>0)$<br>$if\ H/C<4\ (\Delta y<0,\ \Delta x>0)$
$-CH_2$	$\left(\frac{O}{C-1},\frac{H-2}{C-1}\right)$	$(\frac{O/C}{C-1},\ \frac{H/C-2}{C-1})$	$if\ H/C>2\ (\Delta y>0,\ \Delta x>0)$<br>$if\ H/C<2\ (\Delta y<0,\ \Delta x>0)$
$-CO$	$\left(\frac{O-1}{C-1},\frac{H}{C-1}\right)$	$(\frac{O/C-1}{C-1},\ \frac{H/C}{C-1})$	$if\ O/C>1\ (\Delta y>0,\ \Delta x>0)$<br>$if\ O/C<1\ (\Delta y>0,\ \Delta x<0)$
$-CO_2$	$\left(\frac{O-2}{C-1},\frac{H}{C-1}\right)$	$(\frac{O/C-2}{C-1},\ \frac{H/C}{C-1})$	$if\ O/C>2\ (\Delta y>0,\ \Delta x>0)$<br>$if\ O/C<2\ (\Delta y>0,\ \Delta x<0)$
$-H_2O$	$\left(\frac{O-1}{C},\frac{H-2}{C}\right)$	$(\frac{-1}{C},\frac{-2}{C})$	$\Delta y/\Delta x=2,\ \Delta y<0,\ \Delta x<0$
$-H_2$	$\left(\frac{O}{C},\frac{H-2}{C}\right)$	$(0, \frac{-2}{C})$	$\Delta y<0,\ \Delta x=0$

Table S22. Conditions for an elevated modified aromaticity index (AI_{mod}) from the initial "precursor" to the final "product" based on the possible gas-releasing reactions

		AI_{mod} expressions	conditions for $AI_{mod,f}$ >$AI_{mod,i}$
$AI_{mod,i}$		x/y	/
	–HNCO	$(x+0.5)/(y+0.5)$	higher if $x/y > 1$
	–HCN	x/y	always equal
	$–NH_3$	$(x+2)/(y+1)$	higher if $x/y < 2$
	$–CH_4$	$(x+1)/(y–1)$	always higher
$AI_{mod,f}$	$–CH_2$	$x/(y–1)$	always higher
	–CO	$(x–0.5)/(y–0.5)$	higher if $x/y > 1$
	$–CO_2$	x/y	always equal
	$–H_2O$	$(x+1.5)/(y+0.5)$	higher if $x/y < 2$
	$–H_2$	$(x+1)/y$	always higher

Rreferences

(1) Li, Y.; Pöschl, U.; Shiraiwa, M. Molecular Corridors and Parameterizations of Volatility in the Chemical Evolution of Organic Aerosols. *Atmos. Chem. Phys.* **2016**, *16* (5), 3327–3344.

(2) Goering, H. K.; Soest, P. J. V. *Forage Fiber Analyses (Apparatus, Reagents, Procedures, and Some Applications)*; U.S. Agricultural Research Service, 1970.

(3) Song, F.; Li, T.; Zhang, J.; Wang, X.; Bai, Y.; Giesy, J. P.; Xing, B.; Wu, F. Novel Insights into the Kinetics, Evolved Gases, and Mechanisms for Biomass (Sugar Cane Residue) Pyrolysis. *Environ. Sci. Technol.* **2019**, *53* (22), 13495–13505.

(4) Liu, J.; Huang, S.; Chen, K.; Wang, T.; Mei, M.; Li, J. Preparation of Biochar from Food Waste Digestate: Pyrolysis Behavior and Product Properties. *Bioresour. Technol.* **2020**, *302*, 122841.

(5) Opfermann, J.; Kaisersberger, E. An Advantageous Variant of the Ozawa-Flynn-Wall Analysis. *Thermochim. Acta* **1992**, *203*, 167–175.

(6) Mallick, D.; Poddar, M. K.; Mahanta, P.; Moholkar, V. S. Discernment of Synergism in Pyrolysis of Biomass Blends Using Thermogravimetric Analysis. *Bioresour. Technol.* **2018**, *261*, 294–305.

(7) Patuzzi, F.; Roveda, D.; Mimmo, T.; Karl, J.; Baratieri, M. A Comparison between On-Line and off-Line Tar Analysis Methods Applied to Common Reed Pyrolysis. *Fuel* **2013**, *111*, 689–695.

(8) Noda, I. Two-Dimensional Infrared Spectroscopy. *J. Am. Chem. Soc.* **1989**, *111* (21), 8116–8118.

(9) Peng, S.; Wang, F.; Wei, D.; Wang, C.; Ma, H.; Du, Y. Application of FTIR Two-Dimensional Correlation Spectroscopy (2D-COS) Analysis in Characterizing Environmental Behaviors of Microplastics: A Systematic Review. *J. Environ. Sci.* **2025**, *147*, 200–216.

(10) Noda, I.; Ozaki, Y. *Two-Dimensional Correlation Spectroscopy: Applications in Vibrational and Optical Spectroscopy*; John Wiley & Sons, 2005.

(11) Li, T.; Ruan, M.; Cao, Y.; Feng, W.; Song, F.; Bai, Y.; Zhao, X.; Wu, F. Molecular-Level Insights into the Temperature-Dependent Formation Dynamics and Mechanism of Water-Soluble Dissolved Organic Carbon Derived from Biomass Pyrolysis Smoke. *Water Res.* **2024**, *252*, 121176.

(12) Abdulla, H. A. N.; Sleighter, R. L.; Hatcher, P. G. Two Dimensional Correlation Analysis of Fourier Transform Ion Cyclotron Resonance Mass Spectra of Dissolved Organic Matter: A New Graphical Analysis of Trends. *Anal. Chem.* **2013**, *85* (8), 3895–3902.

(13) Dittmar, T.; Koch, B.; Hertkorn, N.; Kattner, G. A Simple and Efficient Method for the Solid-Phase Extraction of Dissolved Organic Matter (SPE-DOM) from Seawater. *Limnol. Oceanogr. Methods* **2008**, *6* (6), 230–235.

(14) Swenson, M. M.; Oyler, A. R.; Minor, E. C. Rapid Solid Phase Extraction of Dissolved Organic Matter. *Limnol. Oceanogr.: Methods* **2014**, *12* (10), 713–728.

(15) Li, M.; Li, Z.; Fu, L.; Deng, L.; Wu, C. Molecular-Level Insights into Dissolved Organic Matter and Its Variations of the Full-Scale Processes in a Typical Petrochemical

Wastewater Treatment Plant. *Water Res.* **2024**, *261*, 121990.
(16) Osterholz, H.; Niggemann, J.; Giebel, H.-A.; Simon, M.; Dittmar, T. Inefficient Microbial Production of Refractory Dissolved Organic Matter in the Ocean. *Nat Commun* **2015**, *6* (1), 7422.
(17) Tolić, N.; Liu, Y.; Liyu, A.; Shen, Y.; Tfaily, M. M.; Kujawinski, E. B.; Longnecker, K.; Kuo, L.-J.; Robinson, E. W.; Paša-Tolić, L.; Hess, N. J. Formularity: Software for Automated Formula Assignment of Natural and Other Organic Matter from Ultrahigh-Resolution Mass Spectra. *Anal. Chem.* **2017**, *89* (23), 12659–12665.
(18) Rockwood, A. L.; Palmblad, M. Isotopic Distributions. In *Mass Spectrometry Data Analysis in Proteomics*; Matthiesen, R., Ed.; Springer: New York, NY, 2020; pp 79–114.
(19) Koch, B. P.; Dittmar, T.; Witt, M.; Kattner, G. Fundamentals of Molecular Formula Assignment to Ultrahigh Resolution Mass Data of Natural Organic Matter. *Anal. Chem.* **2007**, *79* (4), 1758–1763.
(20) Ijaz, A.; Kew, W.; China, S.; Schum, S. K.; Mazzoleni, L. R. Molecular Characterization of Organophosphorus Compounds in Wildfire Smoke Using 21-T Fourier Transform-Ion Cyclotron Resonance Mass Spectrometry. *Anal. Chem.* **2022**, *94* (42), 14537–14545.
(21) Kujawinski, E. B.; Longnecker, K.; Blough, N. V.; Vecchio, R. D.; Finlay, L.; Kitner, J. B.; Giovannoni, S. J. Identification of Possible Source Markers in Marine Dissolved Organic Matter Using Ultrahigh Resolution Mass Spectrometry. *Geochim. Cosmochim. Acta* **2009**, *73* (15), 4384–4399.
(22) Ohno, T.; He, Z.; Sleighter, R. L.; Honeycutt, C. W.; Hatcher, P. G. Ultrahigh Resolution Mass Spectrometry and Indicator Species Analysis to Identify Marker Components of Soil- and Plant Biomass-Derived Organic Matter Fractions. *Environ. Sci. Technol.* **2010**, *44* (22), 8594–8600.
(23) Zhang, X.; Kang, J.; Chu, W.; Zhao, S.; Shen, J.; Chen, Z. Spectral and Mass Spectrometric Characteristics of Different Molecular Weight Fractions of Dissolved Organic Matter. *Sep. Purif. Technol.* **2020**, *253*, 117390.
(24) Van Krevelen, D. W. Graphical-Statistical Method for the Study of Structure and Reaction Processes of Coal. *Fuel* **1950**, *29*, 269–284.
(25) Kim, S.; Kramer, R. W.; Hatcher, P. G. Graphical Method for Analysis of Ultrahigh-Resolution Broadband Mass Spectra of Natural Organic Matter, the van Krevelen Diagram. *Anal. Chem.* **2003**, *75* (20), 5336–5344.
(26) Koch, B. P.; Dittmar, T. From Mass to Structure: An Aromaticity Index for High-Resolution Mass Data of Natural Organic Matter. *Rapid Commun. Mass Spectrom.* **2006**, *20* (5), 926–932.
(27) Koch, B. P.; Dittmar, T. From Mass to Structure: An Aromaticity Index for High-Resolution Mass Data of Natural Organic Matter. *Rapid Commun. Mass Spectrom.* **2016**, *30* (1), 250–250.
(28) Lv, J.; Zhang, S.; Wang, S.; Luo, L.; Cao, D.; Christie, P. Molecular-Scale Investigation with ESI-FT-ICR-MS on Fractionation of Dissolved Organic Matter Induced by Adsorption on Iron Oxyhydroxides. *Environ. Sci. Technol.* **2016**, *50* (5), 2328–2336.
(29) Zhao, J.; Wang, Z.; Li, J.; Yan, B.; Chen, G. Pyrolysis of Food Waste and Food Waste

Solid Digestate: A Comparative Investigation. *Bioresour. Technol.* **2022**, *354*, 127191.
(30) Opatokun, S. A.; Kan, T.; Al Shoaibi, A.; Srinivasakannan, C.; Strezov, V. Characterization of Food Waste and Its Digestate as Feedstock for Thermochemical Processing. *Energy Fuels* **2016**, *30* (3), 1589–1597.
(31) Ren, P.; Ling, T.-C.; Mo, K. H. CO2 Pretreatment of Municipal Solid Waste Incineration Fly Ash and Its Feasible Use as Supplementary Cementitious Material. *J. Hazard. Mater.* **2022**, *424*, 127457.
(32) Wang, X.; Sheng, L.; Yang, X. Pyrolysis Characteristics and Pathways of Protein, Lipid and Carbohydrate Isolated from Microalgae Nannochloropsis Sp. *Bioresour. Technol.* **2017**, *229*, 119–125.
(33) Yang, H.; Yan, R.; Chen, H.; Lee, D. H.; Zheng, C. Characteristics of Hemicellulose, Cellulose and Lignin Pyrolysis. *Fuel* **2007**, *86* (12), 1781–1788.
(34) Qiao, Y.; Wang, B.; Zong, P.; Tian, Y.; Xu, F.; Li, D.; Li, F.; Tian, Y. Thermal Behavior, Kinetics and Fast Pyrolysis Characteristics of Palm Oil: Analytical TG-FTIR and Py-GC/MS Study. *Energy Convers. Manag.* **2019**, *199*, 111964.
(35) Rodante, F.; Marrosu, G.; Catalani, G. Thermal Analysis of Some α-Amino Acids with Similar Structures. *Thermochim. Acta* **1992**, *194* (C), 197–213.
(36) Wang, J.; Ma, X.; Yu, Z.; Peng, X.; Lin, Y. Studies on Thermal Decomposition Behaviors of Demineralized Low-Lipid Microalgae by TG-FTIR. *Thermochim. Acta* **2018**, *660*, 101–109.
(37) Peng, W.; Zhang, H.; Lü, F.; Shao, L.; He, P. From Food Waste and Its Digestate to Nitrogen Self-Doped Char and Methane-Rich Syngas: Evolution of Pyrolysis Products during Autogenic Pressure Carbonization. *J. Hazard. Mater.* **2022**, *424*, 127249.
(38) Yao, Q.; Sun, M.; Gao, J.; Wang, R.; Zhang, Y.; Xu, L.; Ma, X. Organic Sulfur Compositions and Distributions of Tars from the Pyrolysis of Solvent Pretreatment Vitrinite of High Sulfur Coal. *J. Anal. Appl. Pyrolysis* **2019**, *139*, 291–300.
(39) Tian, Y.; Zhang, J.; Zuo, W.; Chen, L.; Cui, Y.; Tan, T. Nitrogen Conversion in Relation to NH_3 and HCN during Microwave Pyrolysis of Sewage Sludge. *Environ. Sci. Technol.* **2013**, *47* (7), 3498–3505.
(40) Zou, L.; Song, L.; Li, M.; Wang, X.; Huang, X.; Zhang, Y.; Dong, B.; Zhou, J.; Li, X. Differential Effect of Anaerobic Digestion on Gaseous Products from Sequential Pyrolysis of Three Organic Solid Wastes. *ACS Omega* **2021**, *6* (34), 22103–22113.
(41) Grube, M.; Lin, J. G.; Lee, P. H.; Kokorevicha, S. Evaluation of Sewage Sludge-Based Compost by FT-IR Spectroscopy. *Geoderma* **2006**, *130* (3), 324–333.
(42) Zielińska, A.; Oleszczuk, P.; Charmas, B.; Skubiszewska-Zięba, J.; Pasieczna-Patkowska, S. Effect of Sewage Sludge Properties on the Biochar Characteristic. *J. Anal. Appl. Pyrolysis.* **2015**, *112*, 201–213.
(43) Jin, J.; Li, Y.; Zhang, J.; Wu, S.; Cao, Y.; Liang, P.; Zhang, J.; Wong, M. H.; Wang, M.; Shan, S.; Christie, P. Influence of Pyrolysis Temperature on Properties and Environmental Safety of Heavy Metals in Biochars Derived from Municipal Sewage Sludge. *J. Hazard. Mater.* **2016**, *320*, 417–426.
(44) Keiluweit, M.; Nico, P. S.; Johnson, M. G.; Kleber, M. Dynamic Molecular Structure of

Plant Biomass-Derived Black Carbon (Biochar). *Environ. Sci. Technol.* **2010**, *44* (4), 1247–1253.

(45) Tian, K.; Liu, W.-J.; Qian, T.-T.; Jiang, H.; Yu, H.-Q. Investigation on the Evolution of N-Containing Organic Compounds during Pyrolysis of Sewage Sludge. *Environ. Sci. Technol.* **2014**, *48* (18), 10888–10896.

(46) Lozano, D. C. P.; E. Jones, H.; Reina, T. R.; Volpe, R.; P. Barrow, M. Unlocking the Potential of Biofuels via Reaction Pathways in van Krevelen Diagrams. *Green Chem.* **2021**, *23* (22), 8949–8963.

(47) Song, F.; Li, T.; Wu, F.; Leung, K. M. Y.; Bai, Y.; Zhao, X. Dynamic Evolution and Covariant Response Mechanism of Volatile Organic Compounds and Residual Functional Groups during the Online Pyrolysis of Coal and Biomass Fuels. *Environ. Sci. Technol.* **2022**, *56* (9), 5409–5420.

(48) Hansson, K.-M.; Åmand, L.-E.; Habermann, A.; Winter, F. Pyrolysis of Poly-l-Leucine under Combustion-like Conditions☆. Fuel 2003, 82 (6), 653–660.

(49) Bakshi, S.; Banik, C.; Laird, D. A.; Smith, R.; Brown, R. C. Enhancing Biochar as Scaffolding for Slow Release of Nitrogen Fertilizer. *ACS Sustainable Chem. Eng.* **2021**, *9* (24), 8222–8231.

(50) Song, F.; Li, T.; Wu, F.; Leung, K. M. Y.; Hur, J.; Zhou, L.; Bai, Y.; Zhao, X.; He, W.; Ruan, M. Temperature-Dependent Molecular Evolution of Biochar-Derived Dissolved Black Carbon and Its Interaction Mechanism with Polyvinyl Chloride Microplastics. *Environ. Sci. Technol.* **2023**, *57* (18), 7285–7297.

(51) Chernyak, S. A.; Ivanov, A. S.; Strokova, N. E.; Maslakov, K. I.; Savilov, S. V.; Lunin, V. V. Mechanism of Thermal Defunctionalization of Oxidized Carbon Nanotubes. *J. Phys. Chem. C* **2016**, *120* (31), 17465–17474.

(52) Chen, W.; Yang, H.; Chen, Y.; Xia, M.; Chen, X.; Chen, H. Transformation of Nitrogen and Evolution of N-Containing Species during Algae Pyrolysis. *Environ. Sci. Technol.* **2017**, *51* (11), 6570–6579.

(53) Gallois, N.; Templier, J.; Derenne, S. Pyrolysis-Gas Chromatography–Mass Spectrometry of the 20 Protein Amino Acids in the Presence of TMAH. *J. Anal. Appl. Pyrolysis.* **2007**, *80* (1), 216–230.

(54) Meetani, M. A.; Zahid, O. K.; Michael Conlon, J. Investigation of the Pyrolysis Products of methionine- enkephalin- Arg- Gly- Leu Using Liquid Chromatography–Tandem Mass Spectrometry. *J. Mass Spectrom.* **2010**, *45* (11), 1320–1331.

(55) Hao, J.; Guo, J.; Ding, L.; Xie, F.; Xia, Q.; Xie, J. TG-FTIR, Py-Two-Dimensional GC–MS with Heart-Cutting and LC–MS/MS to Reveal Hydrocyanic Acid Formation Mechanisms during Glycine Pyrolysis. *J Therm Anal Calorim* **2014**, *115* (1), 667–673.

(56) Huang, J.; Liu, C.; Wei, S.; Huang, X.; Li, H. Density Functional Theory Studies on Pyrolysis Mechanism of β-d-Glucopyranose. *J. Mol. Struct. THEOCHEM.* **2010**, *958* (1), 64–70.

(57) Chen, P.; Gong, M.; Chen, Y.; Zhou, Z.; Liu, M.; Fang, Y.; Chen, W.; Yang, H.; Chen, H. Thermal Decomposition Pathways of Phenylalanine and Glutamic Acid and the Interaction Mechanism between the Two Amino Acids and Glucose. *Fuel* **2022**, *324*, 124345.

(58) Maher, K. D.; Kirkwood, K. M.; Gray, M. R.; Bressler, D. C. Pyrolytic Decarboxylation and Cracking of Stearic Acid. *Ind. Eng. Chem. Res.* **2008**, *47* (15), 5328–5336.
(59) Jiang, G.; Nowakowski, D. J.; Bridgwater, A. V. Effect of the Temperature on the Composition of Lignin Pyrolysis Products. *Energy Fuels* **2010**, *24* (8), 4470–4475.
(60) Wang, L.; Liang, M.; Fang, Y.; Yin, J.; Jiang, J.; Zhang, Y.; Yang, H. A New Insight of Lignin Pyrolysis Characteristics Based on Dehydrogenation Polymers (DHPs). *Fuel Process. Technol.* **2022**, *236*, 107397.
(61) Gunanathan, C.; Milstein, D. Applications of Acceptorless Dehydrogenation and Related Transformations in Chemical Synthesis. *Science* **2013**, *341* (6143), 1229712.
(62) Pujro, R.; García, J. R.; Bertero, M.; Falco, M.; Sedran, U. Review on Reaction Pathways in the Catalytic Upgrading of Biomass Pyrolysis Liquids. *Energy Fuels* **2021**, *35* (21), 16943–16964.
